# Fast, greener and scalable direct coupling of organolithium compounds with no additional solvents

**DOI:** 10.1038/ncomms11698

**Published:** 2016-06-02

**Authors:** Erik B. Pinxterhuis, Massimo Giannerini, Valentín Hornillos, Ben L. Feringa

**Affiliations:** 1 Faculty of Mathematics and Natural Sciences, Stratingh Institute for Chemistry, University of Groningen, Nijenborgh 4, 9747AG Groningen, The Netherlands

## Abstract

Although the use of catalytic rather than stoichiometric amounts of metal mediator in cross-coupling reactions between organic halides and organometallic counterparts improves significantly the atom economy and waste production, the use of solvents and stoichiometric generation of main-group byproducts (B, Sn and Zn) hamper the ‘greenness' and industrial efficiency of these processes. Here we present a highly selective and green Pd-catalysed cross-coupling between organic halides and organolithium reagents proceeding without additional solvents and with short reaction times (10 min). This method bypasses a number of challenges previously encountered in Pd-catalysed cross-coupling with organolithium compounds such as strict exclusion of moisture, dilution and slow addition. Operational ease of this protocol combines the use of industrially viable catalysts loadings (down to 0.1 mol%), scalability of the process (tested up to 120 mmol) and exceptionally favourable environmental impact (E factors in several cases as low as 1).

## Abstract (web summary)

Cross-coupling reactions typically are carried out under dilute conditions of ethereal solvents. Here, the authors report a method for palladium catalysed cross-coupling of organolithium reagents that avoids the use of any added solvent with reaction times of only minutes.

## Graphical abstract

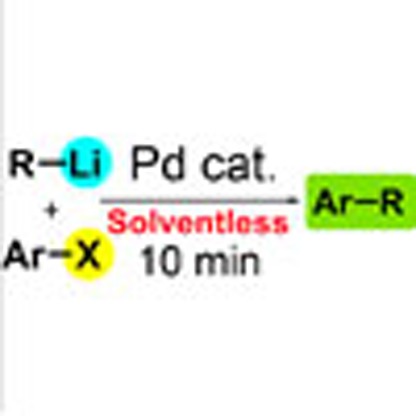

The development of greener, more efficient and simple reaction methodologies sets a priority for the synthetic chemistry community both in industry and in academia as well^1^. Solvents are mainly responsible for the environmental impact of synthetic procedures, being, in general, the largest contributors to the magnitude of the E factor (E=organic waste (kg)/product (kg)), a value introduced by Sheldon *et al*.^2^ to measure the ‘greenness' of a chemical process^3^. Thus, reduction or elimination of solvents from organic reactions is of major concern in chemical process development^4^,^5^,^6^,^7^. Higher energy needs, toxicity, safety hazards and massive waste treatment are direct implications of the use of large volumes of solvents that negatively affect both costs and environment. Inspired by the 12 principles of Green Chemistry^8^, the development of sustainable products is committed to reduce or, possibly, prevent the use of traditional solvents that still, as today, represent the major share of chemical waste production (up to 80%).

An ideal solution to the above-mentioned issues is to completely exclude the solvent from the reaction medium. These so-called solvent-free conditions often lead to additional improvements in other critical parameters as well, such as the catalytic loading (generally lower), the speed of the reaction (generally higher) and the volume/output ratio^9^.

A particular challenging class of transformations in this respect are the widely used transition metal-catalysed reactions. Despite the central role played by Pd-catalysed cross-coupling reactions of organometallic compounds with organo-(pseudo)halides, both in industrial^10^,^11^ and in academic laboratories^12^,^13^,^14^,^15^, the corresponding solvent-free variants have been scarcely reported. Although boron compounds have been engaged in solvent-free cross-coupling reactions, thus far the use of microwaves^16^,^17^, ball mill^18^,^19^ and/or high temperatures are required (Fig. 1a).

Our group has recently described methods for the palladium-catalysed direct cross-coupling of highly reactive organolithium reagents^20^,^21^ (among the most versatile and widely used reagents in organic synthesis) with organic halides under mild conditions^22^,^23^,^24^,^25^,^26^,^27^,^28^,^29^. The extreme reactivity of organometallic reagents such as organolithium compounds commonly dictates highly delicate conditions such as low temperatures, dilution, slow addition and so on, to achieve high conversion and selectivity in their chemical transformations. In the case of Pd-catalysed cross-coupling reactions directly applying organolithium compounds, the use of toluene as a solvent and slow addition of a previously diluted solution of organolithium reagent are key factors to obtain high selectivity and good yields, while avoiding the notorious lithium–halogen exchange and homocoupling side reactions (Fig. 1b)^30^,^31^,^32^.

Despite the formidable challenge presented by the quest to control selectivity when mixing organolithium reagents with neat organohalides, owing to the possibility for numerous competing reactions, we show here the development of the first Pd-catalysed solvent-free cross-coupling of highly polar organometallic compounds that, through a concise and simple procedure, affords the desired coupled product with excellent selectivities within 10 min and in many cases with E factors as low as 1 (Fig. 1c).

## Results

### Preliminary observations

Inspired by the report of García-Álvarez and colleagues^33^ on the use of deep eutectic solvents (DES, mostly obtained by mixing a quaternary ammonium salt as choline chloride with a hydrogen-bond donor such as glycerol or water) for the 1,2-addition of Grignard and organolithium reagents to ketones, we set out to explore the Pd-catalysed cross-coupling reaction of organolithium compounds and organic halides employing these green solvents. Despite the high reactivity of organolithium compounds towards protic solvents, we were delighted to find that the reaction between an excess of PhLi (2–10 eq) and 1-bromonaphthalene using 10 mol% of Pd catalyst in a type III DES proceeded with good selectivity, although in low yield (28–53% conversion, see Supplementary Table 1). We hypothesized that probably small droplets of substrate containing high concentration of catalyst were formed, and that the reaction was taking place directly in the organic phase rather than in the DES phase. However, owing to quenching of the organolithium reagent by the solvent, the conversions obtained were low. We questioned whether the innate reactivity of organolithium compounds could be turned into an inherent advantage offering the possibility to develop a solvent-free Pd-catalysed cross-coupling protocol, which proceeds within minutes, without the support of any additional device (microwave, ball mills and so on), with low catalytic loading and at ambient temperature without the use of strictly inert conditions.

### Reaction conditions optimization

We set out to investigate the reaction between 4-methoxybromobenzene **1a**, a reluctant aryl bromide in coupling reactions^34^, and commercially available phenyllithium, as successful conditions for the coupling of these two substrates would most probably apply to a wide variety of other coupling partners as well (Table 1). All the reactions were carried out by adding the organolithium compound (without prior dilution) to a neat mixture of catalyst and organic halide over 10 min at room temperature (RT). Moreover, we employed a 1-mmol scale to illustrate the synthetic utility of the method. Reactions using the *in situ*-prepared palladium complex Pd/XPhos (generated by mixing Pd_2_(dba)_3_ with XPhos)^35^, previously reported to be effective for other Pd-catalysed cross-coupling reactions with aryllithium reagents^25^,^26^, afforded the cross-coupling product **2a** (*R*=Ph) within 10 min, although in the presence of significant amounts of the undesired homocoupling product (Table 1, entry 1). By employing Pd-PEPPSI-IPent catalyst^36^, the selectivity was raised to 90% at the expense of the homocoupling product (Table 1, entry 2). Nonetheless, we were delighted to find that the commercially available air- and temperature-stable Pd-PEPPSI-IPr catalyst, which is seven times cheaper than Pd-PEPPSI-IPent^37^, afforded full conversion and nearly perfect selectivity (>95%,) towards the coupled product **2a** (*R*=Ph) at RT in <10 min, avoiding the formation of dehalogenation or homocoupling side products **3** and **4** (Table 1, entry 3). Importantly, the high selectivity was maintained while lowering the catalyst loading to 1.5 mol% (Table 1, entry 4, 84% isolated yield). With an efficient catalyst for C*sp*^2^–C*sp*^*2*^ cross-coupling in hands, we then turn our attention to the challenging C*sp*^3^–C*sp*^*2*^ solvent-free cross-coupling with alkyllithium compounds. The direct use of commercially available *n*-BuLi, one of the most reactive organometallic reagents, in combination with Pd-PEPPSI-IPr led to the desired product **2v** (*R*=*n*-Bu), although with slightly diminished selectivity (Table 1, entry 5). Further screening of catalysts showed that the use of commercially available Pd[P(*t*Bu)_3_]_2_ catalyst^38^ restored the selectivity (>95%) towards the coupled product **2v** (*R*=*n*-Bu) with excellent (82%) isolated yield (Table 1, entry 6). Importantly, when this reaction was performed using an extremely low catalyst loading (0.1 mol %), product **2v** (*R*=*n*-Bu) was still obtained in high conversion and selectivity (Entry 7).

### Scope and applicability

To our delight, the optimized conditions proved to be general and could be applied successfully to the solvent-free cross-coupling of a variety of aryllithium (**2a**–**2u**) and the even more reactive alkyllithium reagents (**2v**–**2af**), in all cases affording the products with high selectivity within minutes (Table 2). The remarkably fast cross-coupling methodology gave excellent results in combination with non-commercially available aryllithium reagents obtained through common preparative procedure, such as lithium/halogen exchange (**2f**–**2h**) and *ortho*-directed lithiation. Illustrative is the case of the highly hindered bis-*ortho*-substituted 2,6-dimethoxy-phenyllithium, used in the synthesis of compounds **2i**–**2k**, which was prepared by direct metalation of 1,3-dimethoxybenzene. In all cases, the organolithium reagents were prepared using the minimal amount of ethereal solvent to maintain them soluble (see Supplementary Methods). Despite the higher reactivity and basicity of alkyllithium reagents when compared with (hetero)aryllithium compounds, we were delighted to find high selectivities and yields also for a variety of C*sp*^3^–C*sp*^*2*^ cross-coupling products. This includes the use of different alkyllithium compounds as *n*-BuLi, *n*-HexLi and the smallest MeLi with electron-rich and electron-poor arylbromides (Table 2, **2v**–**2ad**). The bifunctional C(*sp*^3^)-(trimethylsilyl)methyllithium reagent^28^ also couples with excellent selectivity, providing synthetically versatile benzylsilanes **2ae** and **2af**. The lack of dehalogenated side products from these C*sp*^3^–C*sp*^*2*^ cross-coupling reactions demonstrates that no competing β-hydride elimination/olefin dissociation^24^ occurs (which is another competing pathway besides the formation of dehalogenated and isomerized products). A limitation so far for this protocol employing the Pd[P(*t*-Bu)_3_]_2_-based catalytic system is that secondary alkyllithium reagents such as *i*-PrLi and *s*-BuLi led to the formation of dehalogenation products.

Despite the conditions of highly concentrated reaction partners, various observations highlight how the reaction proceeds exclusively under catalyst control. Thus, the reaction of 1-bromonaphthalene **1b** resulted, with both aryl- and alkyllithium, in the corresponding coupled products (**2b**, **2f**, **2k**, **2x** and **2aa**) without the formation of regioisomers, indicating that benzyne intermediates via 1,2-elimination are not formed. Apart from liquid substrates, even solid bromofluorene **1af** was successfully employed, despite the acidity of the benzylic protons (p*K*_a_=22). The reaction of *n*-BuLi and MeLi with *p*-chloro-bromobenzene occurs selectively with no detectable chloride displacement (Table 2, compounds **2w** and **2ab**).

Sterically hindered bromides **1c**–**1e**, known for being more reluctant substrates in the synthesis of biaryls^23^, were also successfully coupled at RT in 10 min, indicating that the transmetallation step takes place rapidly, under these conditions, inducing a fast coupling process.

The dramatic effect of the solvent-free conditions in enhancing the reaction rate was demonstrated in the coupling of commercially available 2-thienyllithium, which, according to our previous observations, required the addition of stoichiometric amounts of tetramethylethylenediamine as the activating agent and elevated temperatures (40 °C) to react^22^. Under solvent-free conditions, 2-thienyllithium reacted smoothly at RT within 10 min, in high selectivity and yield, without the use of any additive (see compounds **2l**–**2r**). We have recently showed that the cross-coupling of 2-alkoxy-substituted arylbromides with organolithium is plagued by fast bromine–lithium exchange induced by the ortho-directing alkoxy unit. The use of the corresponding aryl chlorides, inherently less prone to halogen/lithium exchange, is thus mandatory, to afford selectively the product and prevent side products formation^27^. However, to our surprise, under our solvent-free protocol, aryl bromides **1n**, **1o** and 3,3′-dibromo-BINOL **1p**, could all be coupled successfully with 2-thienyllithium in high selectivity (>95%) and with excellent yield, avoiding the notorious bromine–halogen exchange (Table 2). To emphasize the versatility of the new method, it has to be noted that the only previous reported synthesis of BINOL derivate **2p** required the preparation of the corresponding bis-trifluoroboronate BINOL derivative and further reaction with 2-bromothiophene under microwave conditions^39^. The use of acetal-protected aldehyde **1q** was also tolerated without the cleavage of the protecting group. As in the case of alkyllithium compounds, 1-bromo-3-chlorobenzene **1r** reacted with 2-thienyllithium using Pd-PEPPSI-IPr catalyst selectively, leaving the chloride untouched. Nevertheless, the electron-poor aryl chloride **1l** reacted readily with 2-thienyllithium and electron-rich chlorides **1t** and **1u** were also easily coupled under the optimized conditions using more reactive PhLi at RT (Table 2).

A major issue often associated with solvent-free reactions is the homogeneity of the reaction medium, in particular with solid starting materials. However, the methodology presented here provides high selectivity and yields when solid substrates such as **1h**, **1p**, **1q**, **1ae** and **1af** were used in combination with aryllithium and TMSCH_2_Li compounds. Unfortunately, the presence of dehalogenated product was observed for the use of *n*-HexLi and *n*-BuLi in combination with solid substrates (for preliminary results using Grignard reagents in similar conditions, see Supplementary Fig. 2).

### Scalability of the protocol

In organic chemistry, problems in the scaling up of batch reactions have been known to arise from various issues including inefficient mixing and lack of heat transfer. To test whether this novel method is suitable to be performed on a larger scale, the cross-coupling between *n*-BuLi and 1-bromonaphthalene **1b** was tested on multigram scale with catalyst loading as low as 0.1 mol%. We were pleased to find that the scale of the reaction had little effect on the selectivity, although the presence of a small amount of dehalogenated side product was observed (Table 3). It is noteworthy that the cross-coupling was found to maintain its effectiveness even at 120 mmol scale employing 0.4 mol% of catalyst, providing exceptional E factors as low as 0.8 (Table 3, entry 3). It should be emphasized that typical E factors in the range of 5–100 are seen in transformations producing fine chemicals and pharmaceuticals^2^. Importantly, after the addition of the organolithium compound, the crude product was quenched, washed with water and dried, giving the desired product in reagent-grade quality within 60 min, including all the operations.

### Potential of the methodology in synthetic application

**Figure 2:**
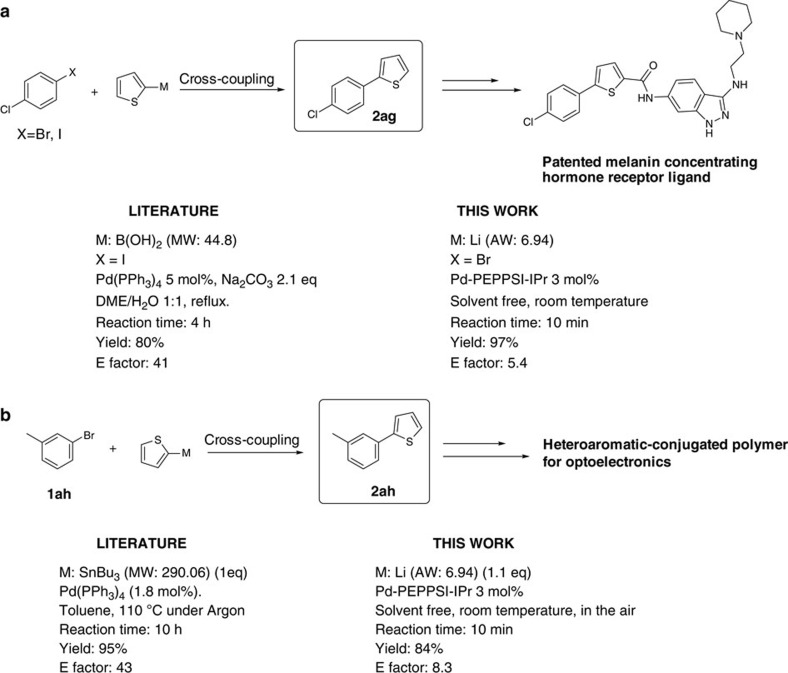
Synthetic applicability. Comparison between established methods and the present cross-coupling protocol with organolithium reagents in the synthesis of key intermediates for a melanin-concentrating hormone receptor ligand (**a**) and conjugated polymer for optoelectronic devices (**b**).

To demonstrate the advantages of the new method, we have compared it with some established cross-coupling methodologies currently used in the production of two typical building blocks for pharmaceuticals and conjugated polymers for light-emitting devices. The first example deals with the preparation of a key intermediate (**2ag**) for the synthesis of a patented melanin concentrating hormone receptor ligand (Fig. 2a)^40^ involved in the treatment of eating disorders, weight gain, obesity, depression and anxiety. The reaction between 1-bromo-4-chlorobenzene **1ag** and 2-thienyllithium under the optimized reaction conditions provided the cross-coupling product **2ag** in high yield and selectivity within 10 min at RT (E factor: 5.4). In sharp contrast, the reported procedure (E factor: 41) involves the corresponding thienylboronic acid, needs a mixture of DME/H_2_O heated at reflux for 4 h, 2 eq of base and requires the corresponding highly reactive aryl iodide. As noted by Lipschutz *et al*.^3^, aqueous workup may also be taken into account in the calculation of the E-factor. In this case, the advantages of using low amounts of solvents become even more evident: although the preparation of **2ag** through conventional Suzuki cross-coupling has an E-factor as high as 84, taking into account the extraction workup, thanks to the reduced amounts of extraction volumes needed, the E-factor obtained for our protocol is 15.4, marking a dramatic difference of 68.6 units between the two protocols. Notably, LiBr is the major byproduct of the reaction.

The second example illustrates the synthesis of a heteroaromatic monomer employed in the preparation of polymeric materials for optoelectronic devices (Fig. 2b) (ref. 41). Coupling between 2-thienyllithium and 3-methyl-1-bromobenzene **1ah** gives access to the desired compound **2ah**, at RT, in very good yield within 10 min and with an E factor five times lower than that reported in synthetic methodology (110 °C, 10 h in toluene using an organotin compound with a molecular weight four times higher than that of 2-thienyllithium). In the last example, in addition to the remarkable differences in reaction time (10 versus 600 min) and temperature (20 °C versus 110 °C), the use of solvent-free cross-coupling of 2-thienyllithium also avoids potentially toxic tin wastes and their often difficult removal, and prevents the use of strictly inert atmosphere required for the coupling of the corresponding tin reagent.

## Discussion

We have discovered that, in sharp contrast to all common reaction protocols using highly reactive organometallics such as organolithium compounds, the Pd-catalysed cross-coupling of arylbromides and alkyl- or aryllithium reagents under neat conditions proceeds exceptionally fast and is selective. Based on this finding we have developed a general solvent-free methodology for the Pd-catalysed direct cross-coupling of organolithium compounds with organic halides, under ambient conditions, providing high yields and excellent selectivities. Fast reaction times (10 min), lower catalyst loading (down to 0.1 mol%), high scalability, operational simplicity (see Methods) and the possibility to avoid the use of a strictly inert atmosphere, of syringe pumps and of additives such as tetramethylethylenediamine are key feature of this methodology. Compared with reported Pd-catalysed cross-coupling, this methodology is particularly attractive, owing to the strongly reduced environmental impact, that is, outstanding volume:time:output ratio, limited amount and low toxicity of the waste and five- to tenfold reduction in E-factor. The use of stable and commercially available catalysts, commercial or readily available and inexpensive organolithium reagents and the applicability to a wide variety of organic bromides are additional factors that contribute to the prospect of these C–C bond formations in the art of synthesis. For supporting videos illustrating the ease of operation, see Supplementary Movies 1, 2.

## Methods

The corresponding organolithium reagent (1.2 eq) was added over a mixture of substrate (1.0 eq) and catalyst (1.5–3 mol%) at RT for 10 min. After the addition was completed, a saturated solution of aqueous NH_4_Cl was added and the mixture was extracted with AcOEt or Et_2_O. The organic phases were combined and dried with anhydrous Na_2_SO_4_. Evaporation of the solvent under reduced pressure afforded the crude product that was then filtered over a silica gel plug to afford the pure product. For NMR spectra of the compounds in this article, see Supplementary Figs 3–73.

### Data availability

The authors declare that the data supporting the findings of this study are available within the article and its Supplementary Information files.

## Additional information

**How to cite this article:** Pinxterhuis, E. B. *et al*. Fast, greener and scalable direct coupling of organolithium compounds with no additional solvents. *Nat. Commun.* 7:11698 doi: 10.1038/ncomms11698 (2016).

## Supplementary Material

Supplementary Information Supplementary Figures 1-73, Supplementary Table 1, Supplementary Notes 1-6, Supplementary Methods and Supplementary References

Supplementary Movie 1 The movie illustrates the cross-coupling between TMSCH2Li and the solid substrate 2-bromo-9H-fluorene (1af) with no additional solvents

Supplementary Movie 2 Cross-coupling at 120 mmol scale employing 0.4 mol % of catalyst

## Figures and Tables

**Figure 1:**
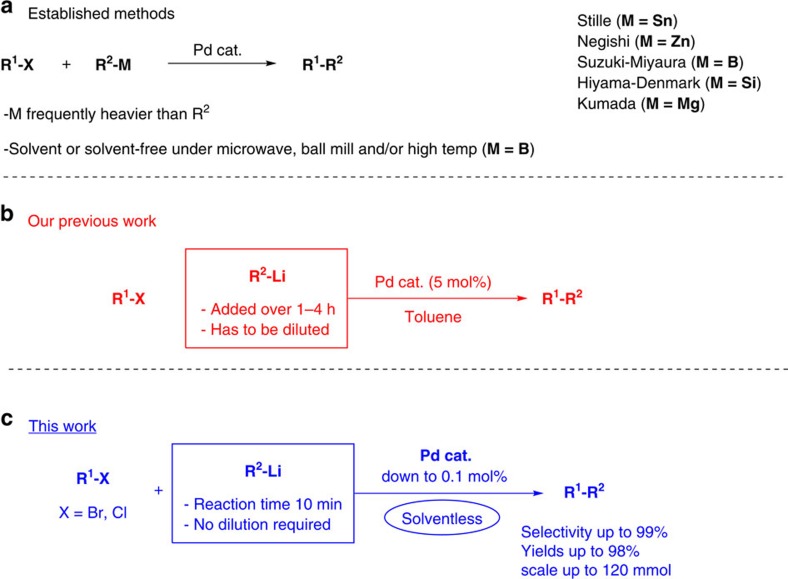
State-of-the-art overview. (**a**) Established methods for Pd-catalysed cross-coupling reactions. (**b**) Catalytic cross-coupling with organolithium compounds. (**c**) A fast, highly scalable and solvent-free direct cross-coupling of organolithium compounds.

**Table 1:**
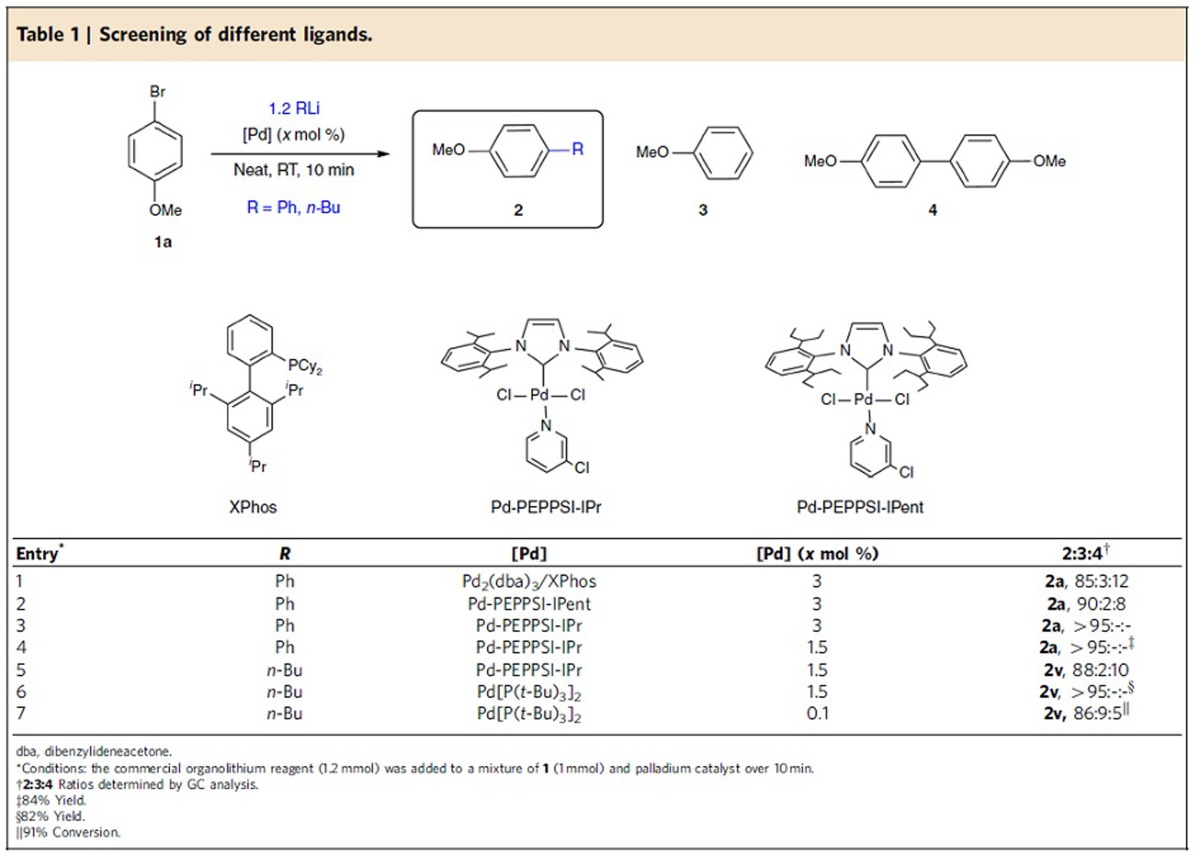
Screening of different ligands.

**Table 2:**
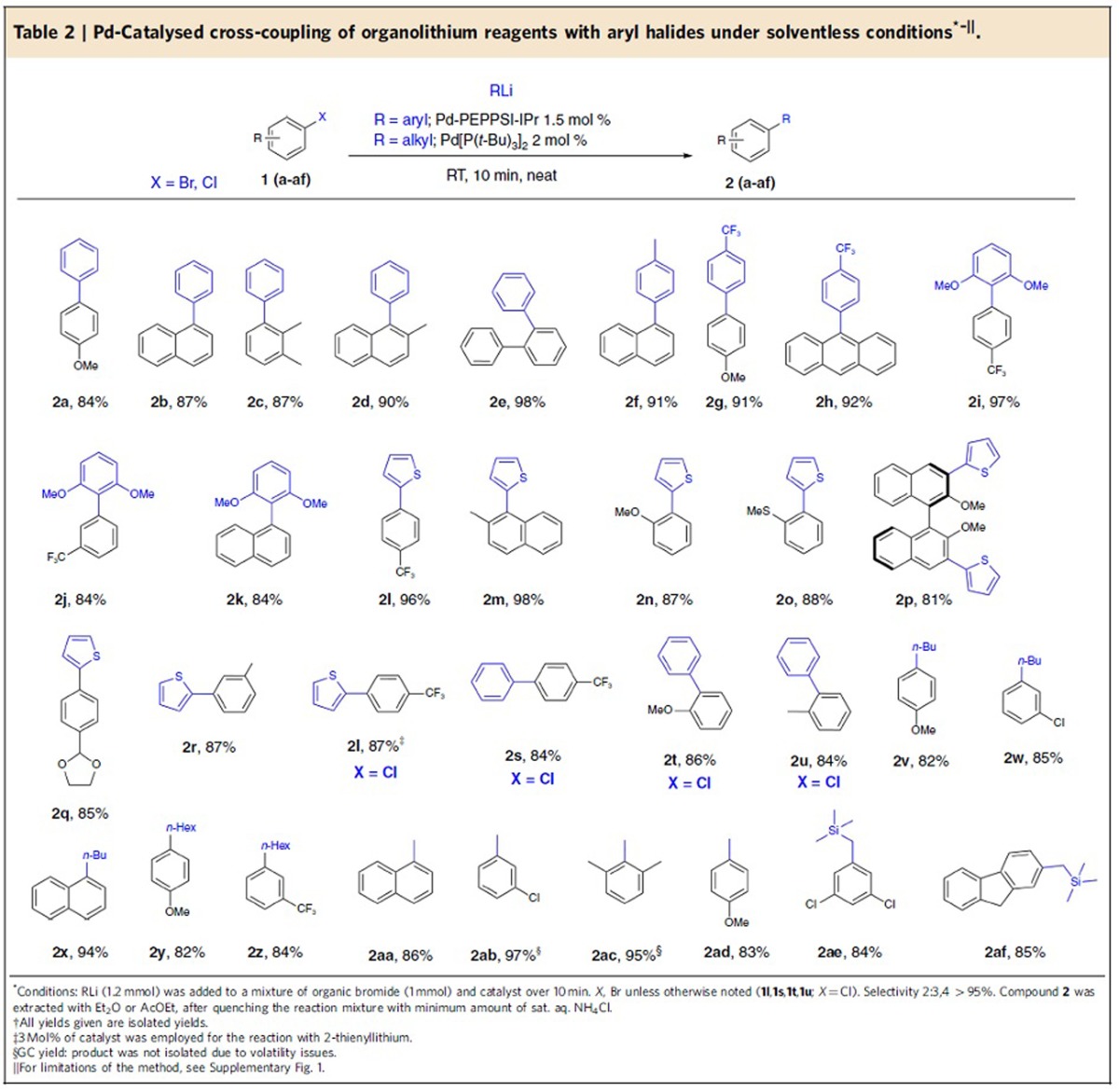
Pd-Catalysed cross-coupling of organolithium reagents with aryl halides under solventless conditions^*–||^.

**Table 3:**
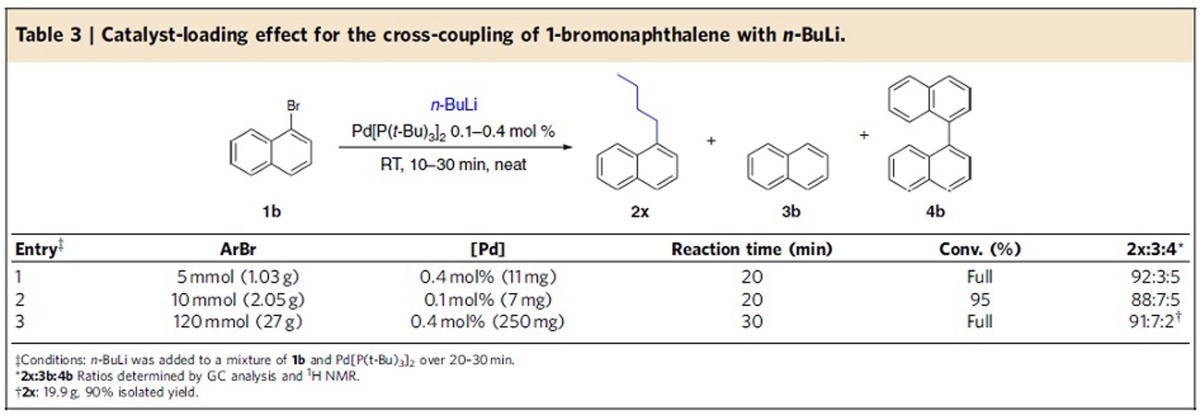
Catalyst-loading effect for the cross-coupling of 1-bromonaphthalene with *n*-BuLi.
