# Supplementary material for: Fast, greener and scalable direct coupling of organolithium compounds with no additional solvents
Source: Nat Commun. 2016 Jun 2;7:11698. doi: 10.1038/ncomms11698 (PMC4895725; doi:10.1038/ncomms11698)
Supplement: Supplementary Information — Supplementary Figures 1-73, Supplementary Table 1, Supplementary Notes 1-6, Supplementary Methods and Supplementary References [file ncomms11698-s1.pdf]

Supplementary Figures

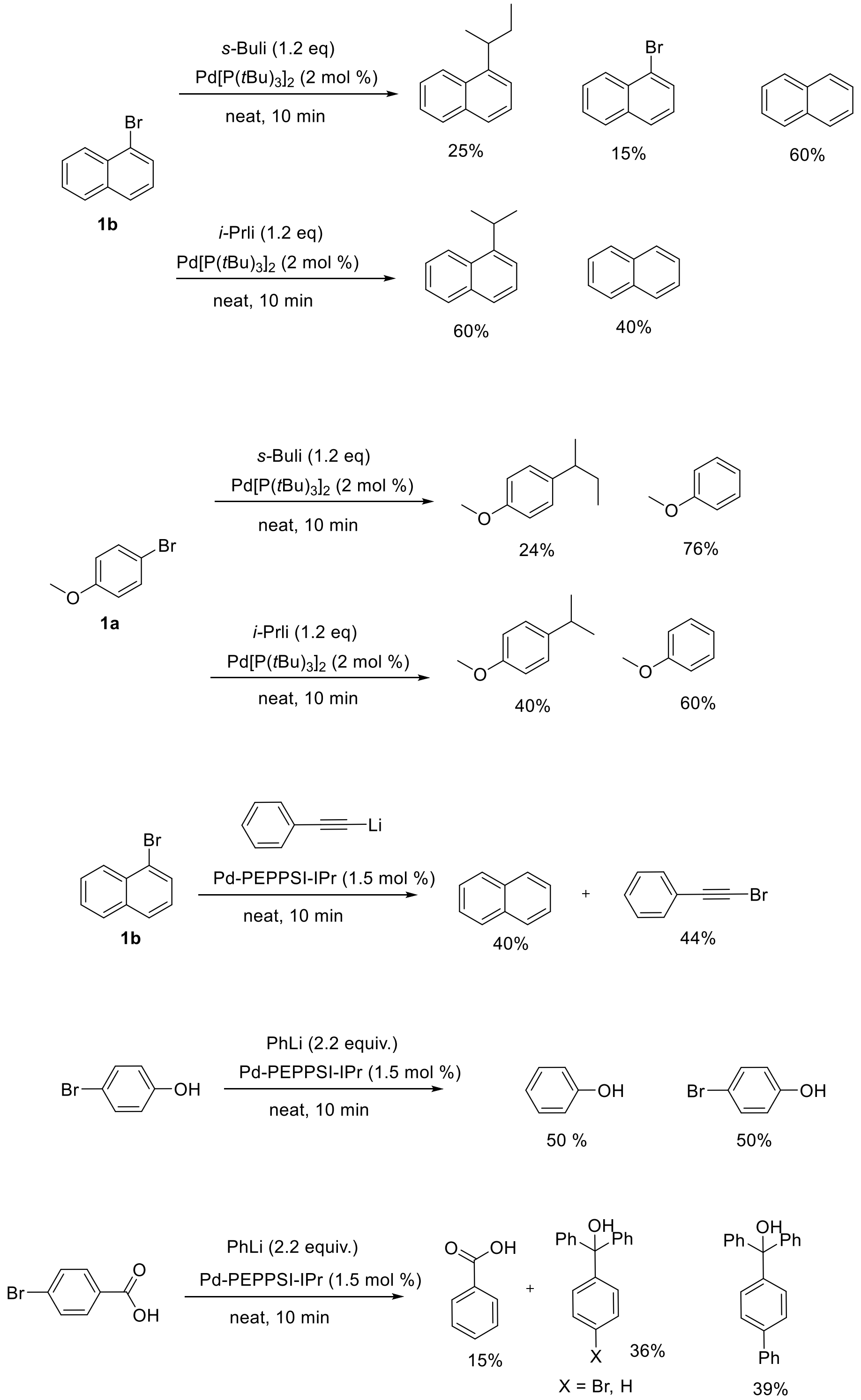

Supplementary Figure 1. Limitations of the method

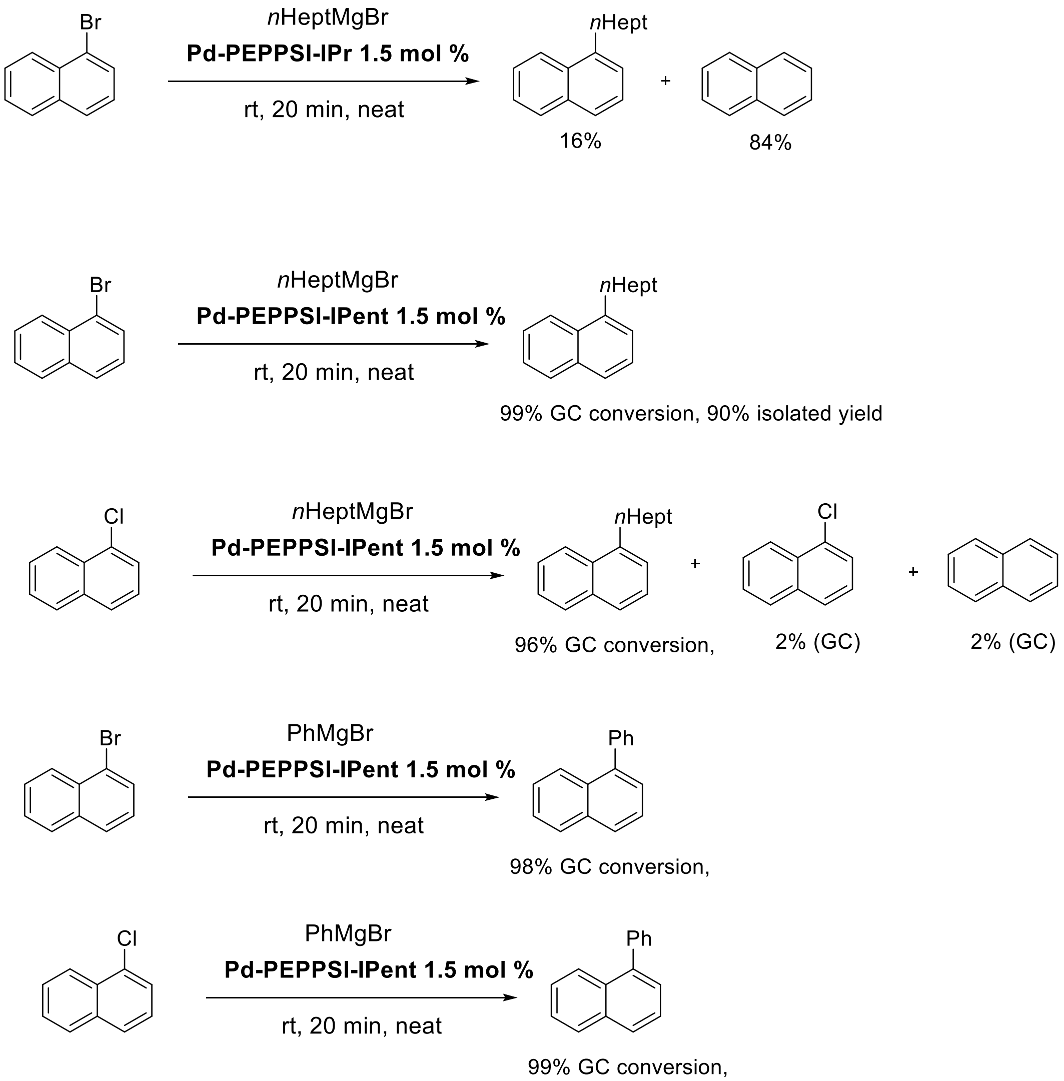

Supplementary Figure 2. Pd-catalysed cross-coupling of *n*-heptMgBr and PhMgBr with 1-chloro- and 1-bromonaphthalene under neat conditions. Conditions: The corresponding commercially available Grignard reagent (1,2 eq) was added over a mixture of substrate (1 mmol) and Pd-PEPPSI-*i*Pent (1.5 mol %, 12.5 mg) at room temperature for 10 min. After the addition was completed, the mixture was stirred for 10 min at room temperature followed by subsequent quenching with a saturated solution of aqueous NH_4Cl (1 mL). The mixture was extracted with Et_2O and the organic phases were combined and dried with anhydrous Na_2SO_4. Evaporation of the solvent under reduced pressure afforded the crude product that was then filtered over a silica gel plug.

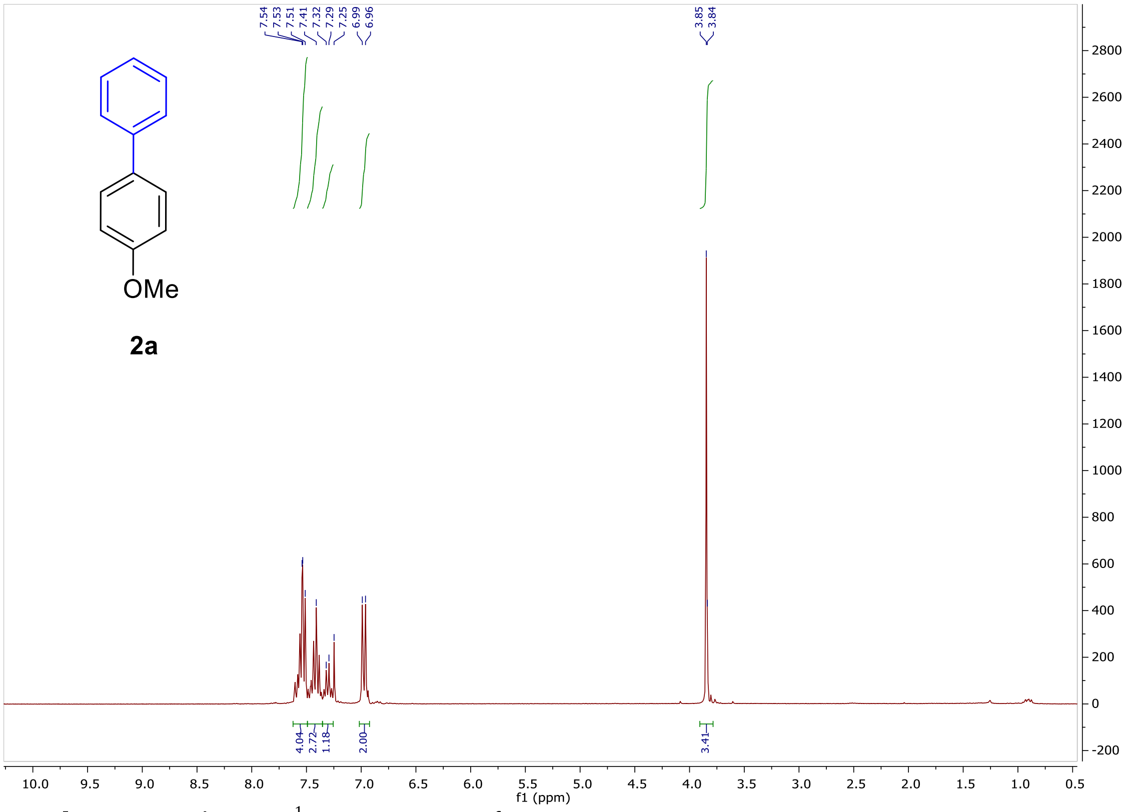

Supplementary Figure 3. ^{1}H NMR spectra of **2a**

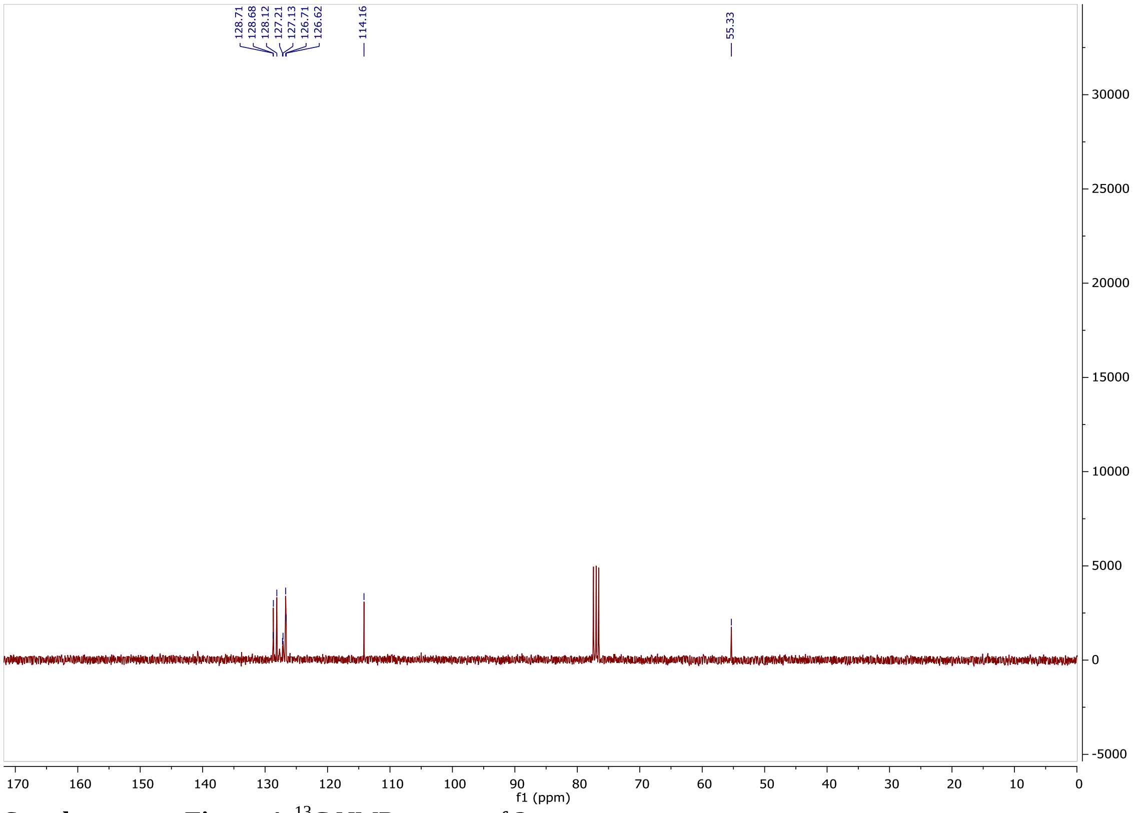

Supplementary Figure 4. ^{13}C NMR spectra of **2a**

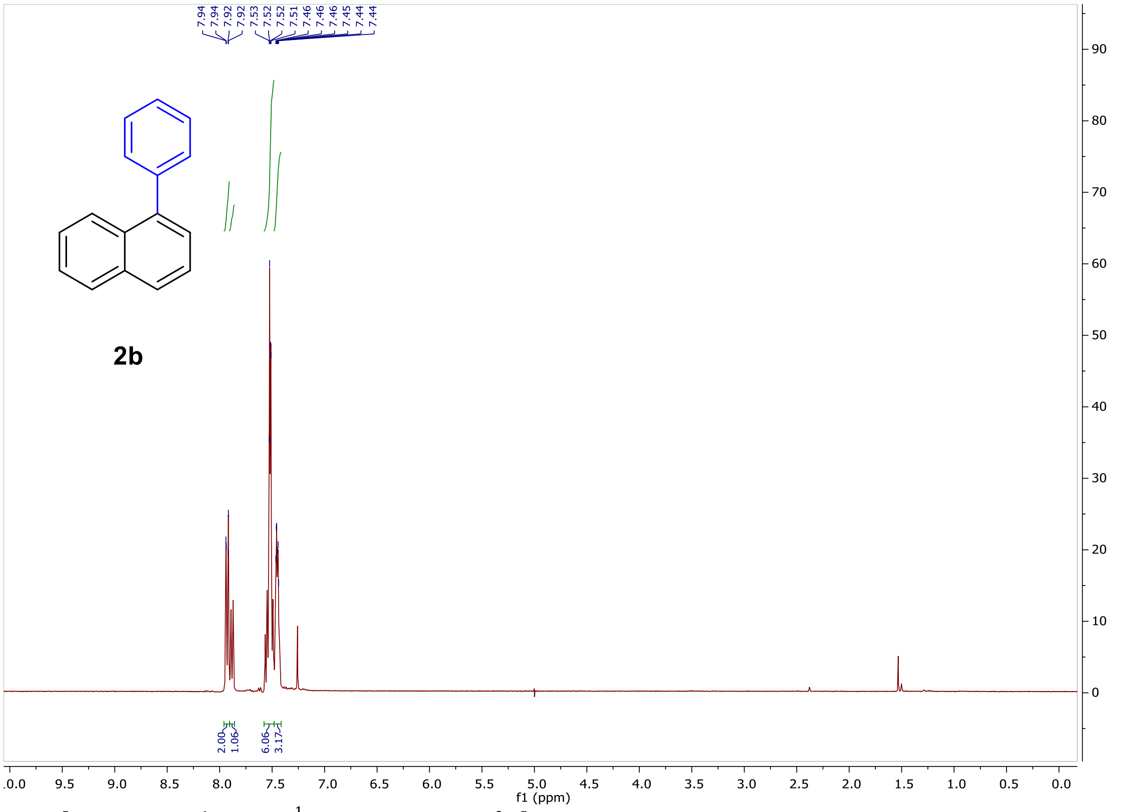

Supplementary Figure 5. ^{1}H NMR spectra of **2b**

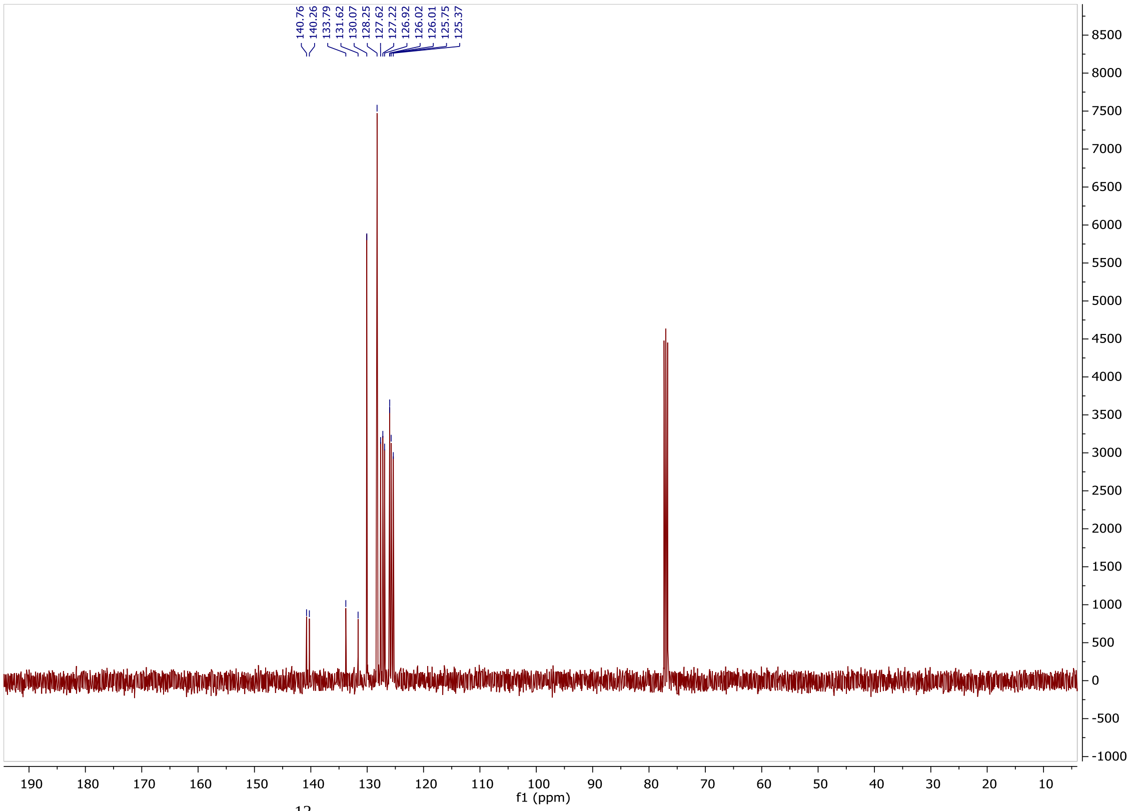

Supplementary Figure 6. ^{13}C NMR spectra of **2b**

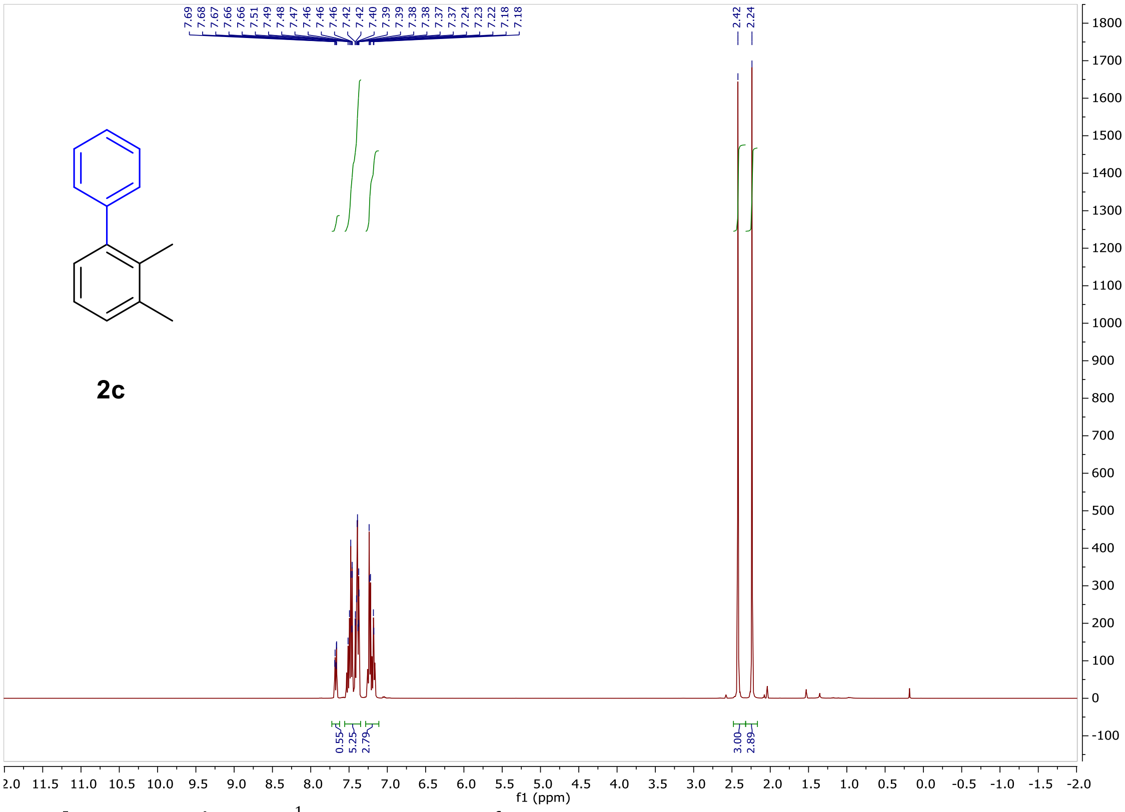

Supplementary Figure 7. ^{1}H NMR spectra of **2c**

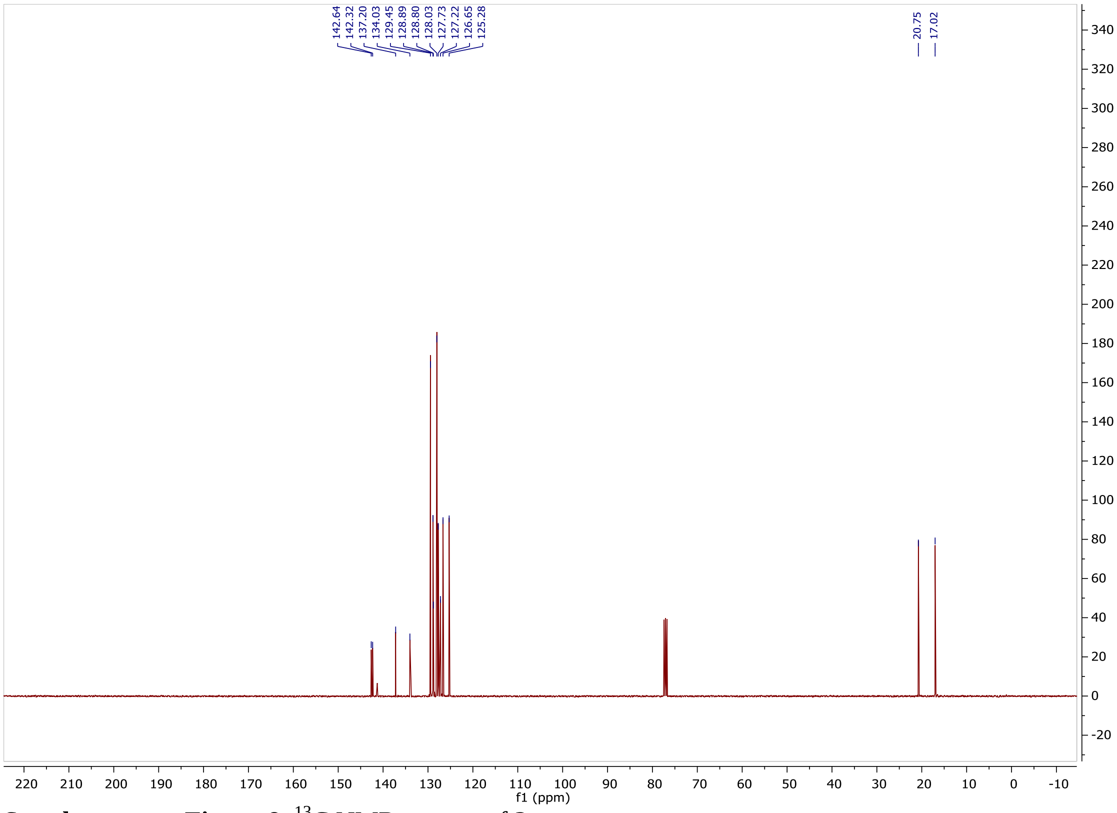

Supplementary Figure 8. ^{13}C NMR spectra of **2c**

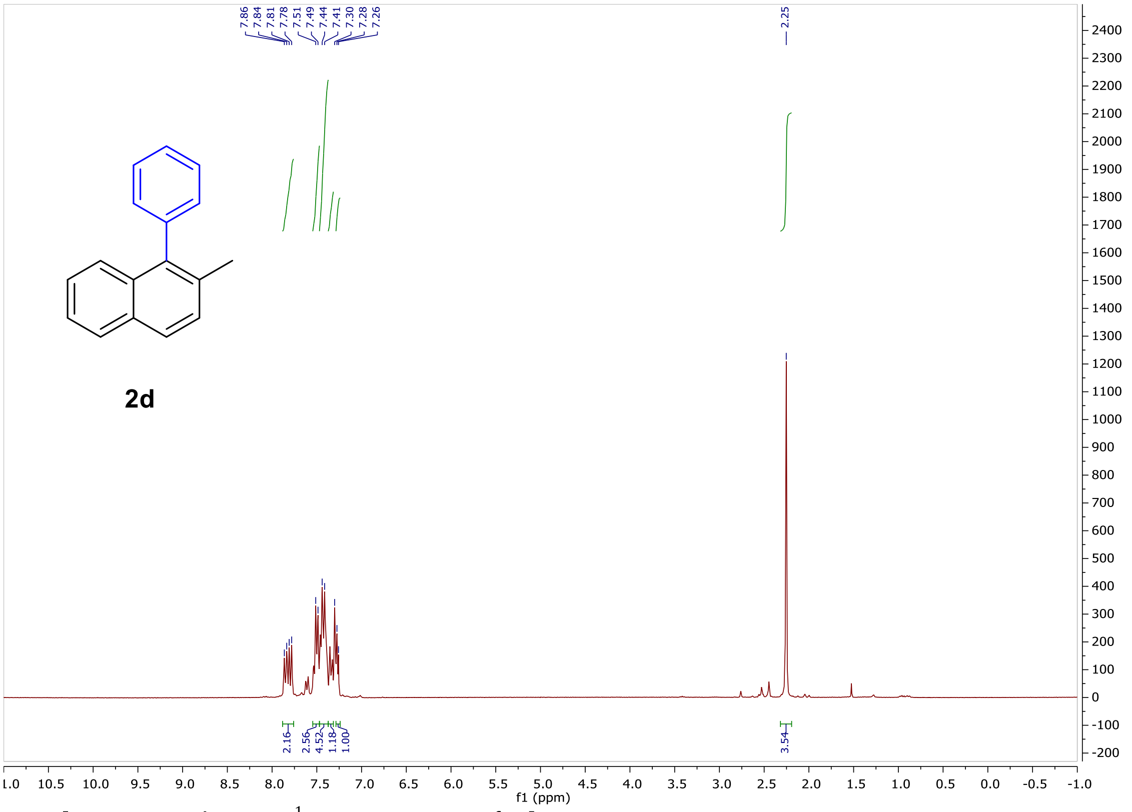

Supplementary Figure 9. ^{1}H NMR spectra of **2d**

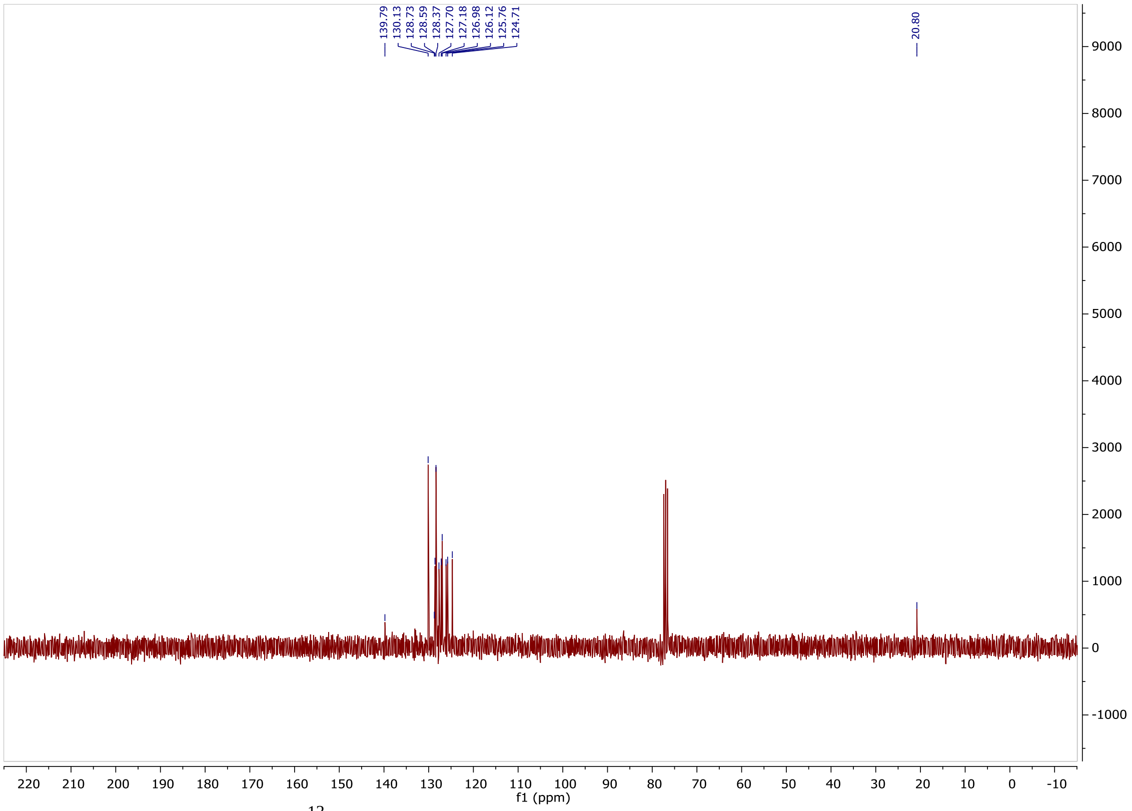

Supplementary Figure 10. ^{13}C NMR spectra of **2d**

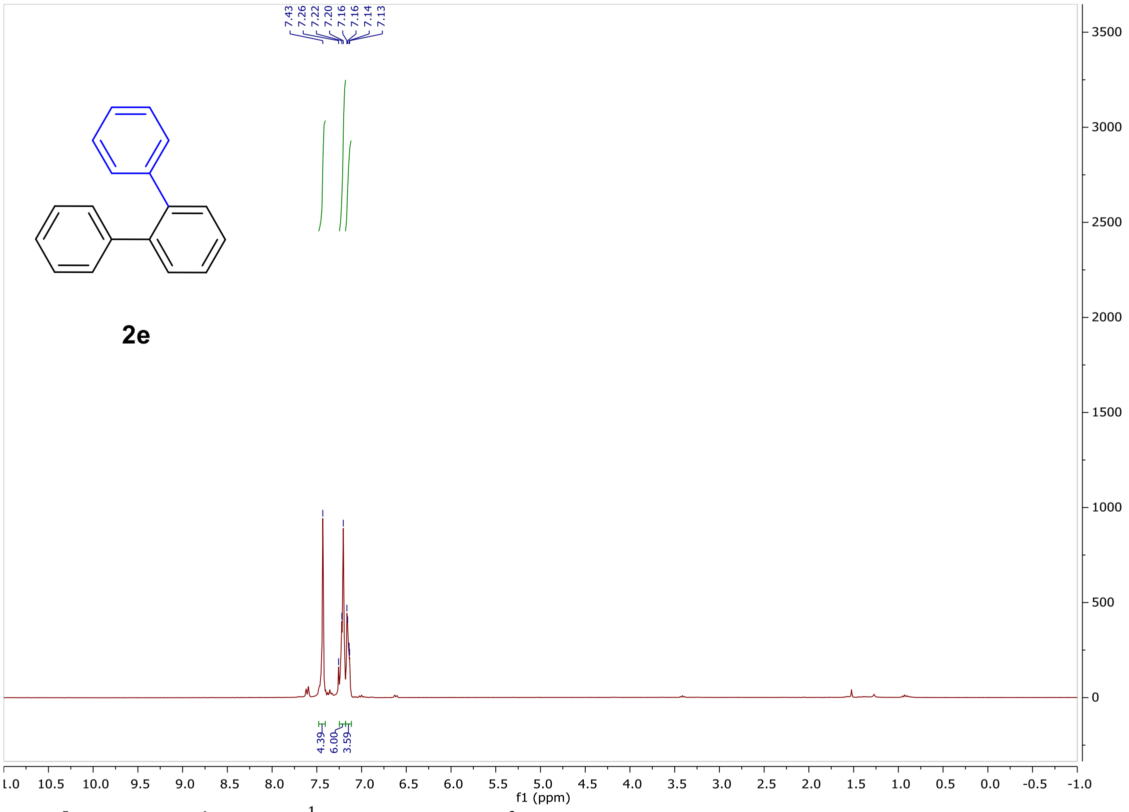

Supplementary Figure 11. ^{1}H NMR spectra of **2e**

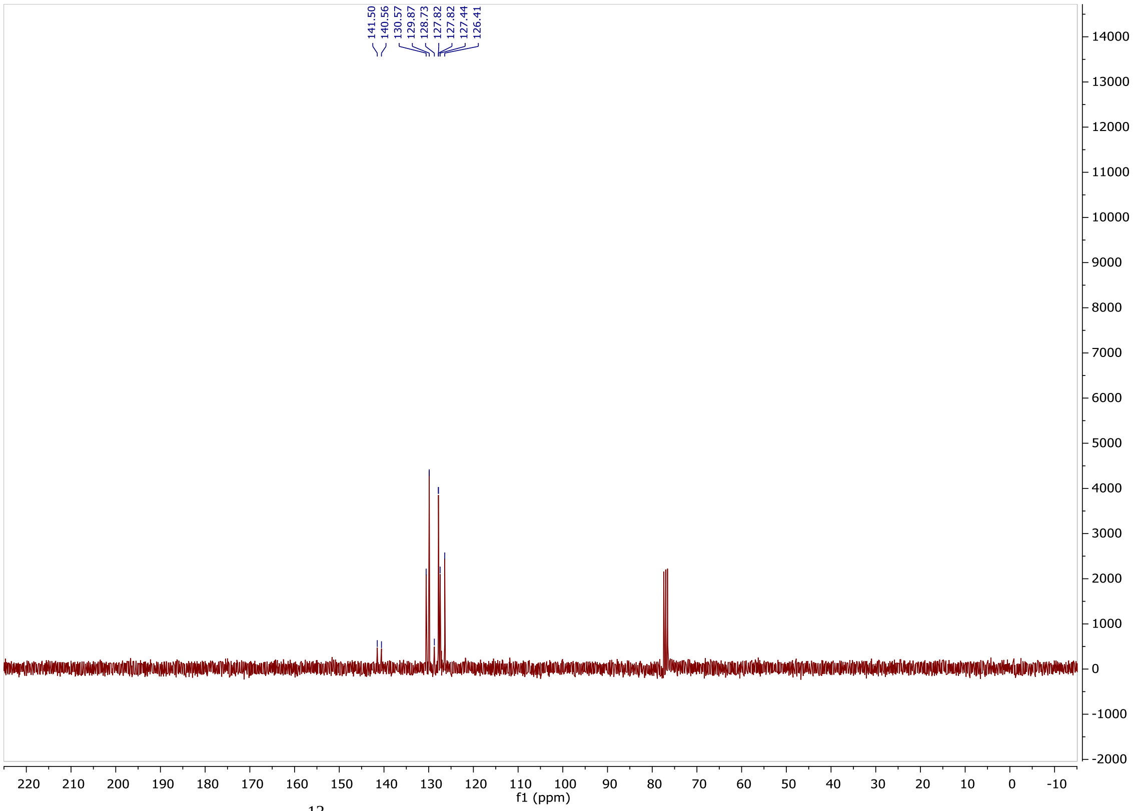

Supplementary Figure 12. ^{13}C NMR spectra of **2e**

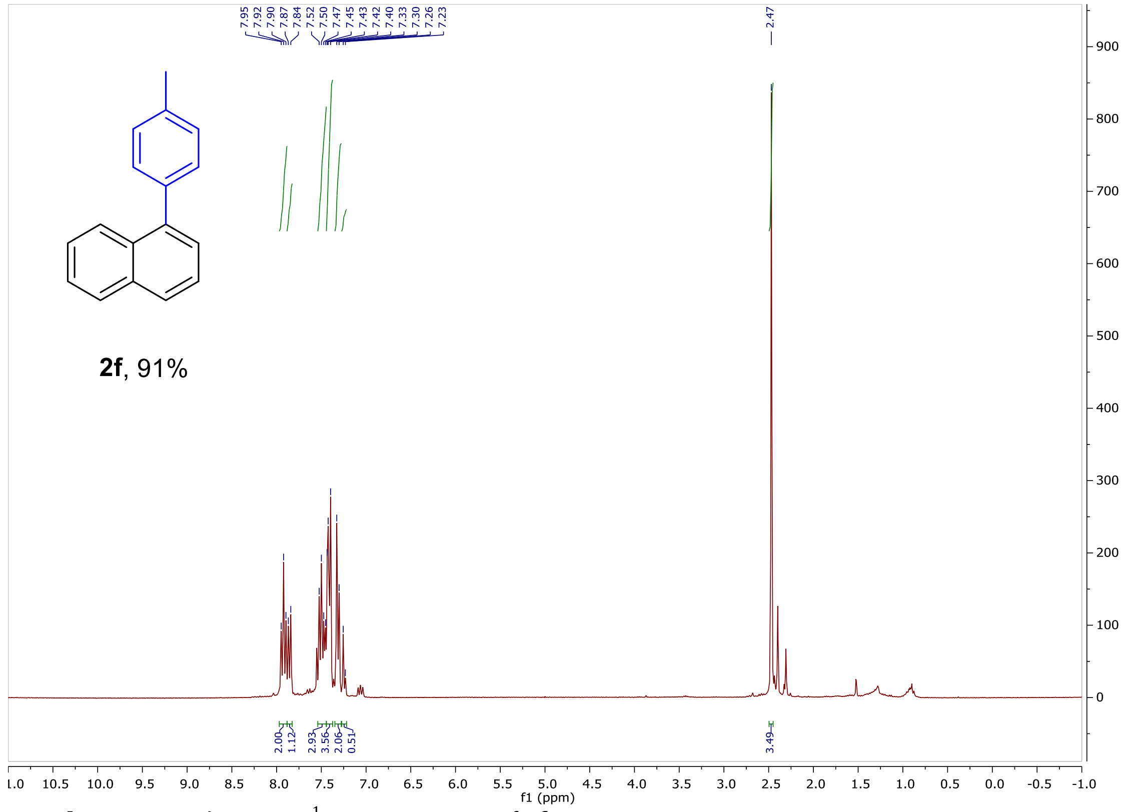

Supplementary Figure 13. ^{1}H NMR spectra of **2f**

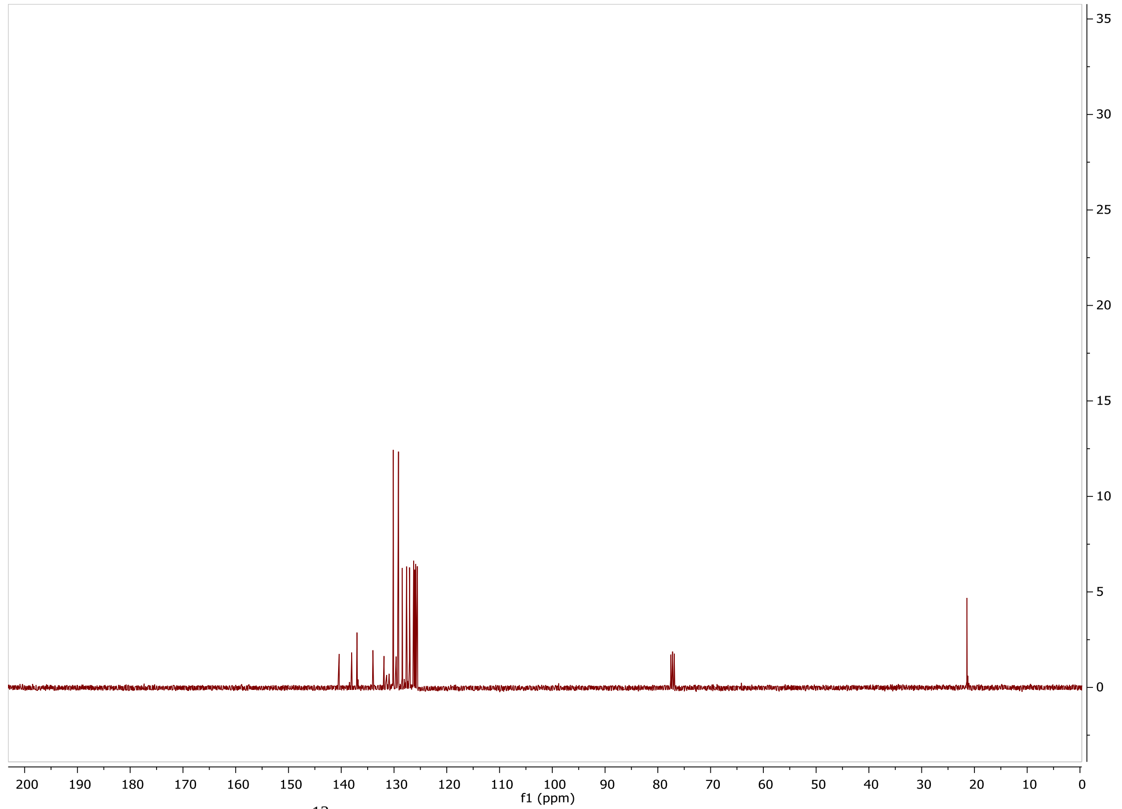

Supplementary Figure 14. ^{13}C NMR spectra of **2f**

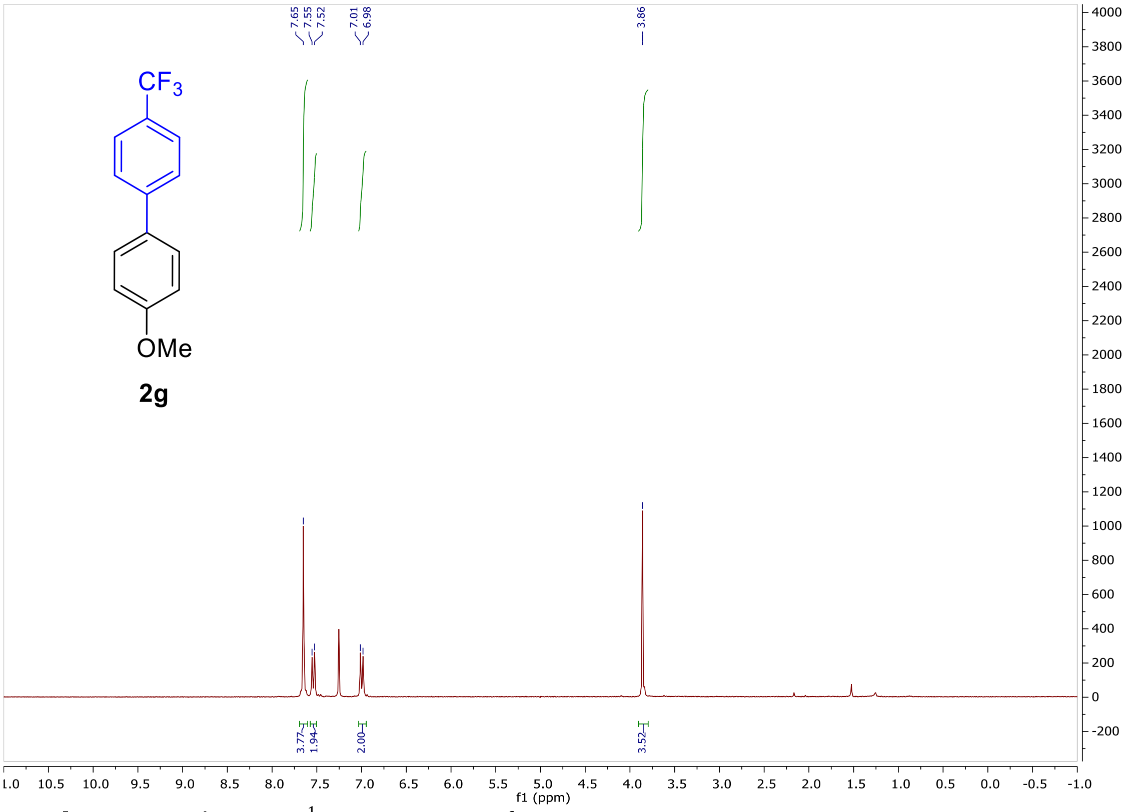

Supplementary Figure 15. ^{1}H NMR spectra of **2g**

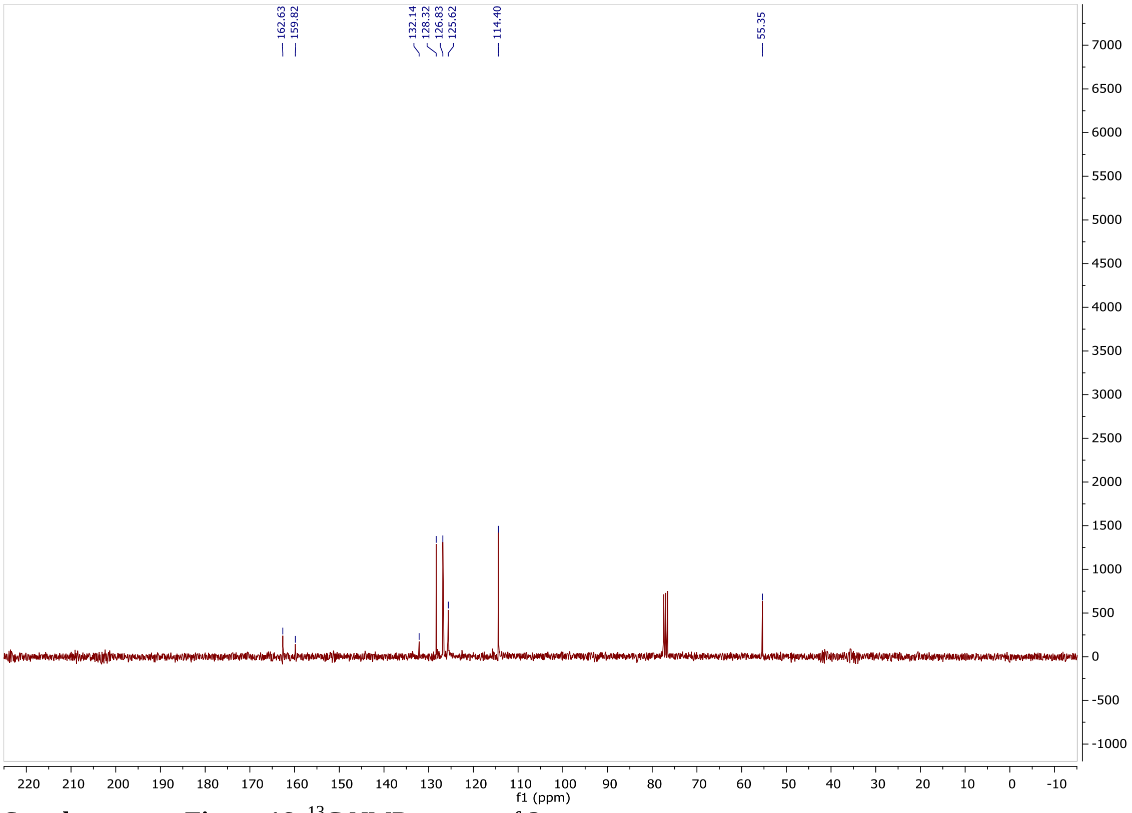

Supplementary Figure 16. ^{13}C NMR spectra of **2g**

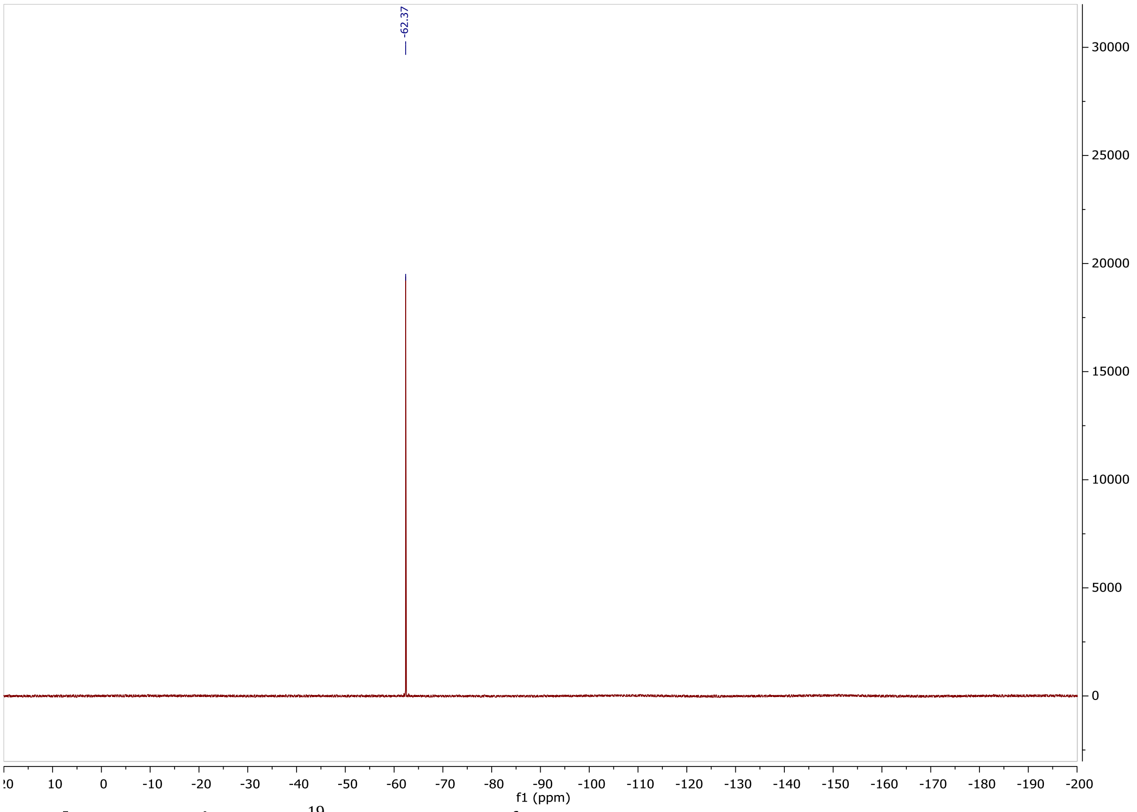

Supplementary Figure 17. ^{19}F NMR spectra of **2g**

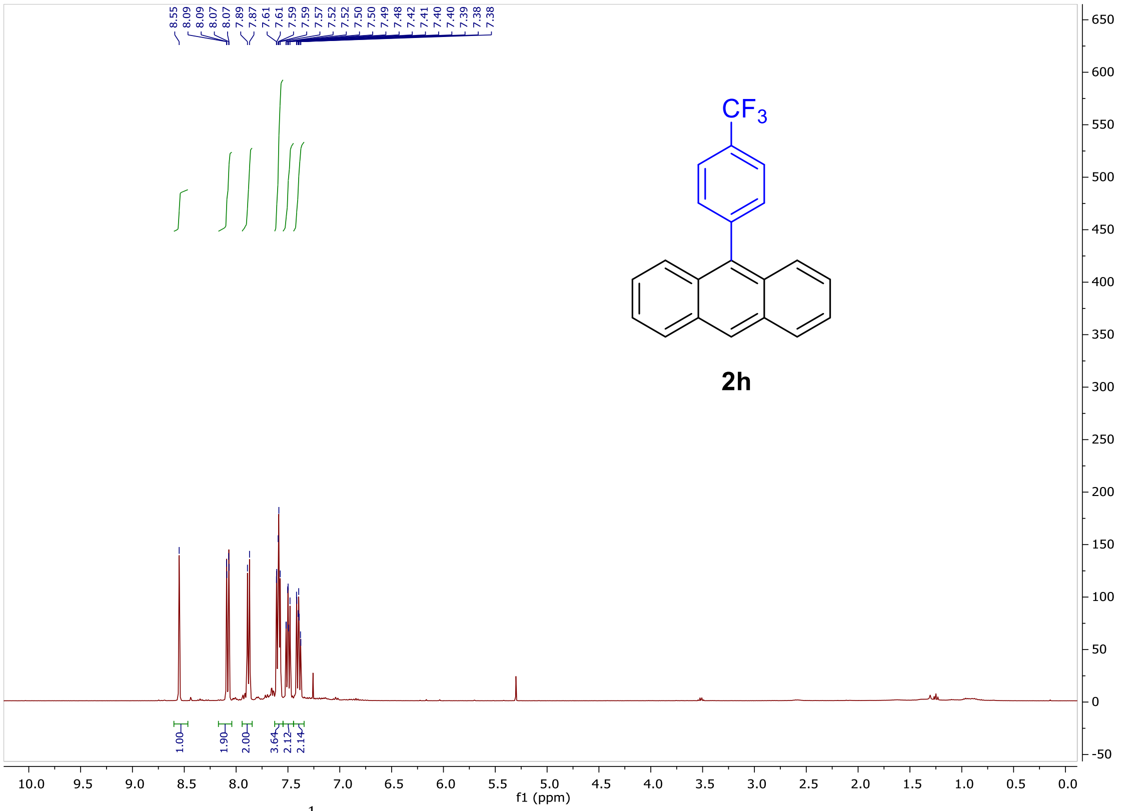

Supplementary Figure 18. ^{1}H NMR spectra of **2h**

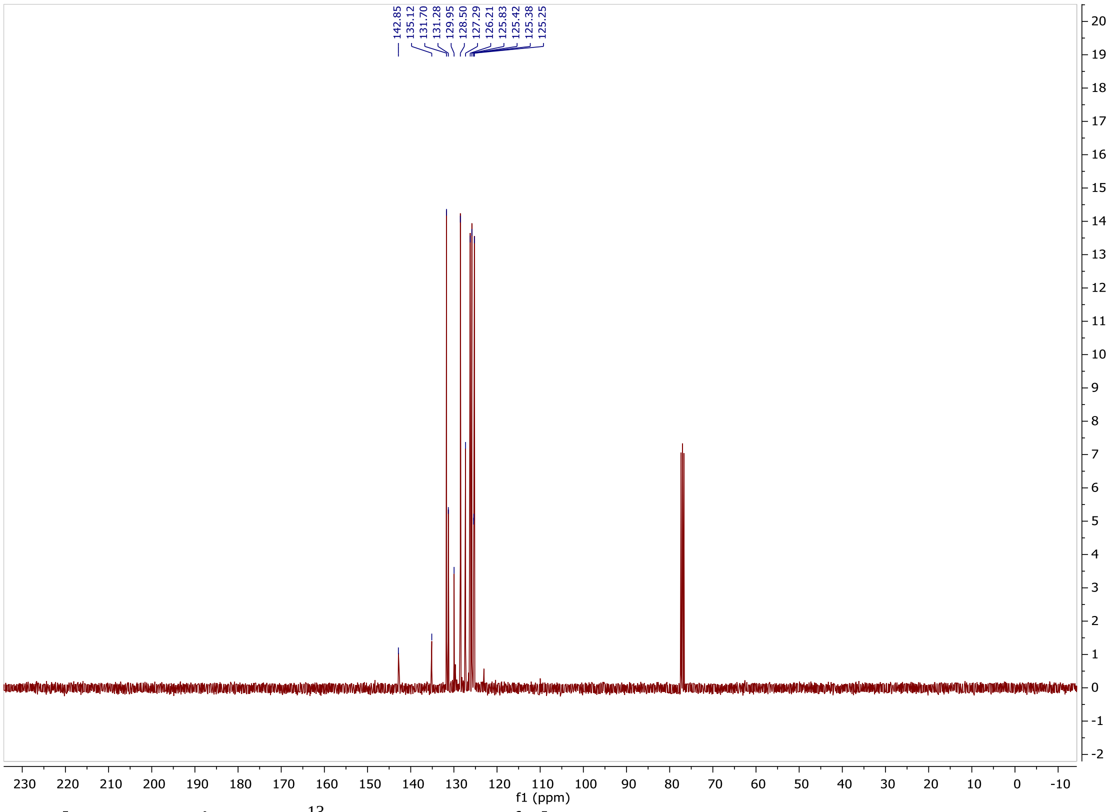

Supplementary Figure 19. ^{13}C NMR spectra of **2h**

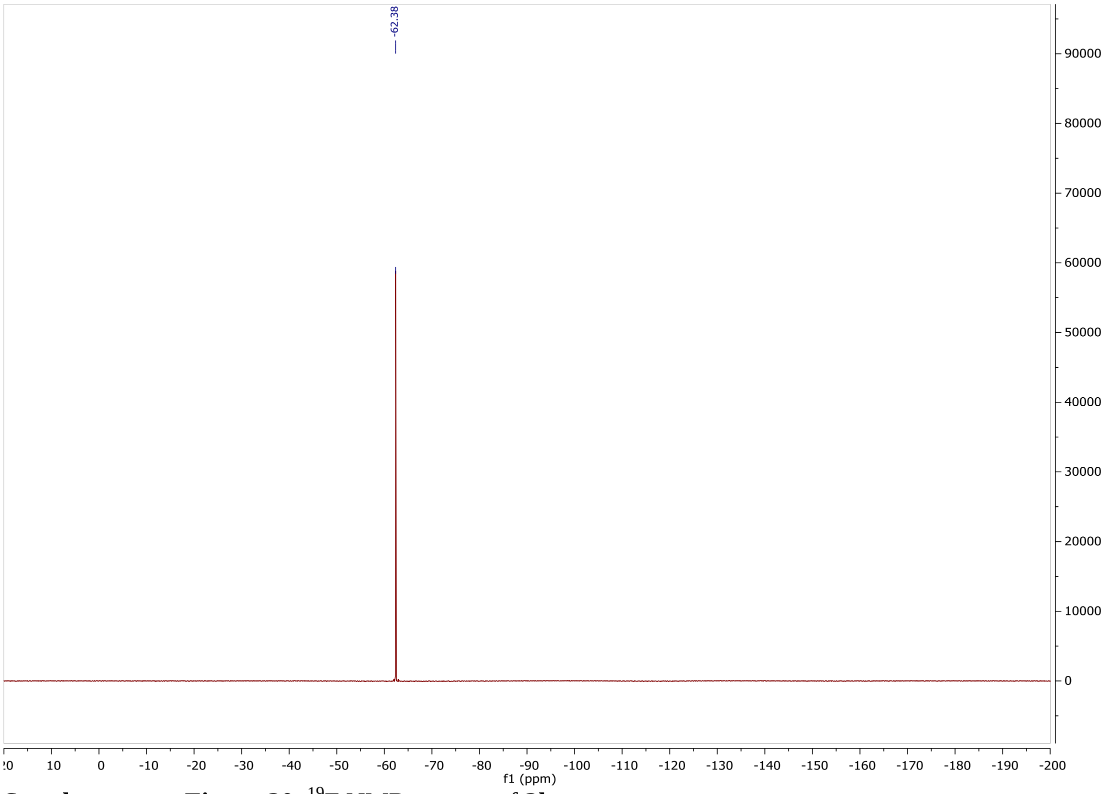

Supplementary Figure 20. ^{19}F NMR spectra of **2h**

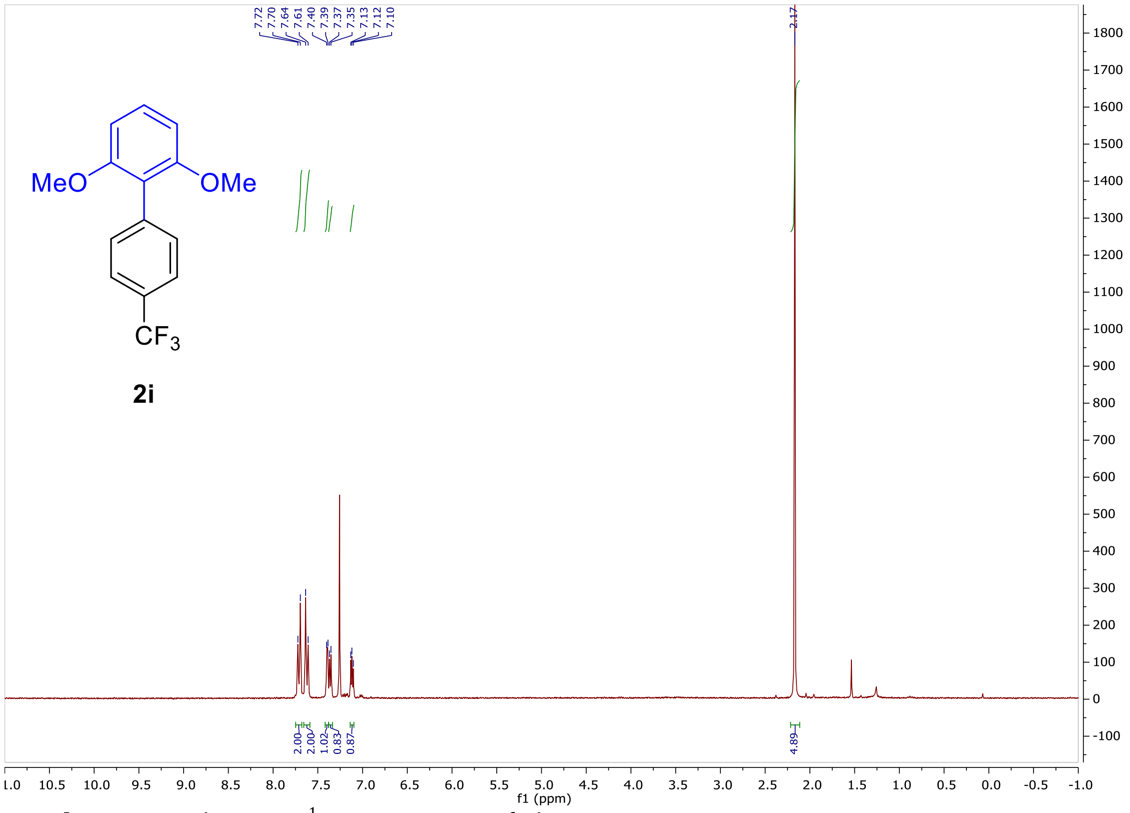

Supplementary Figure 21. ^{1}H NMR spectra of **2i**

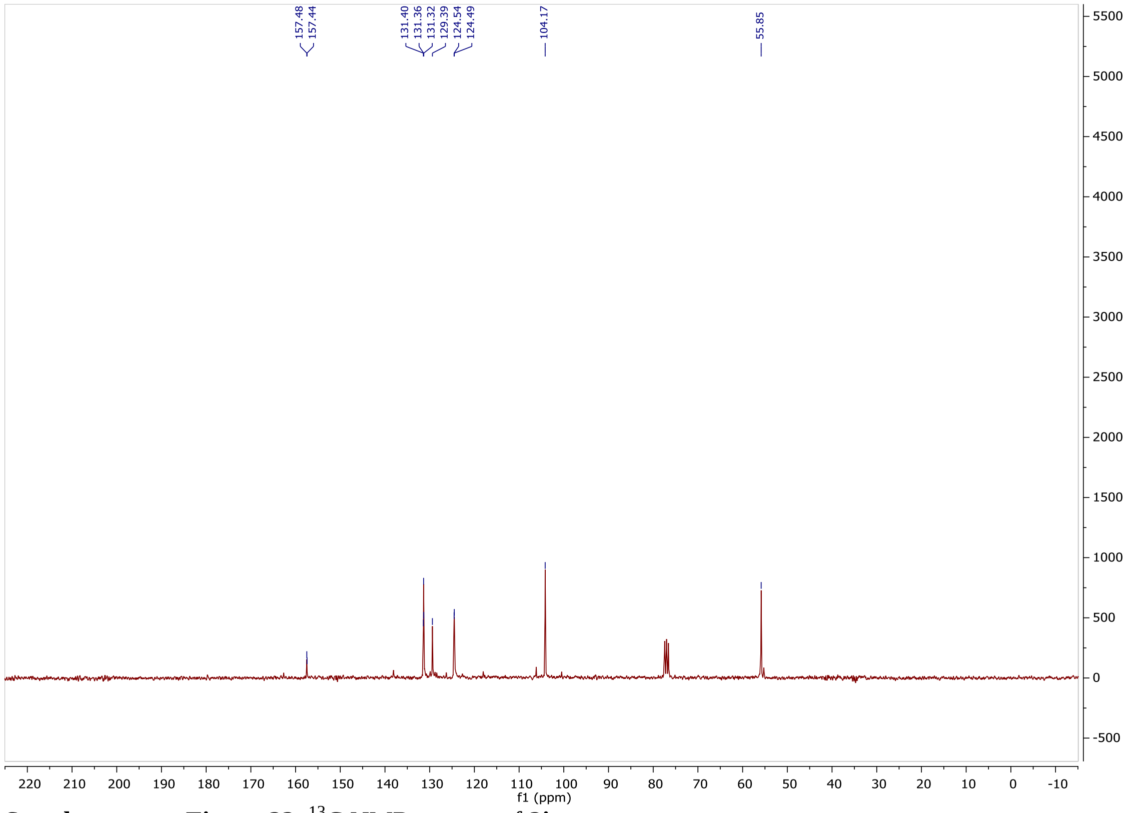

Supplementary Figure 22. ^{13}C NMR spectra of **2i**

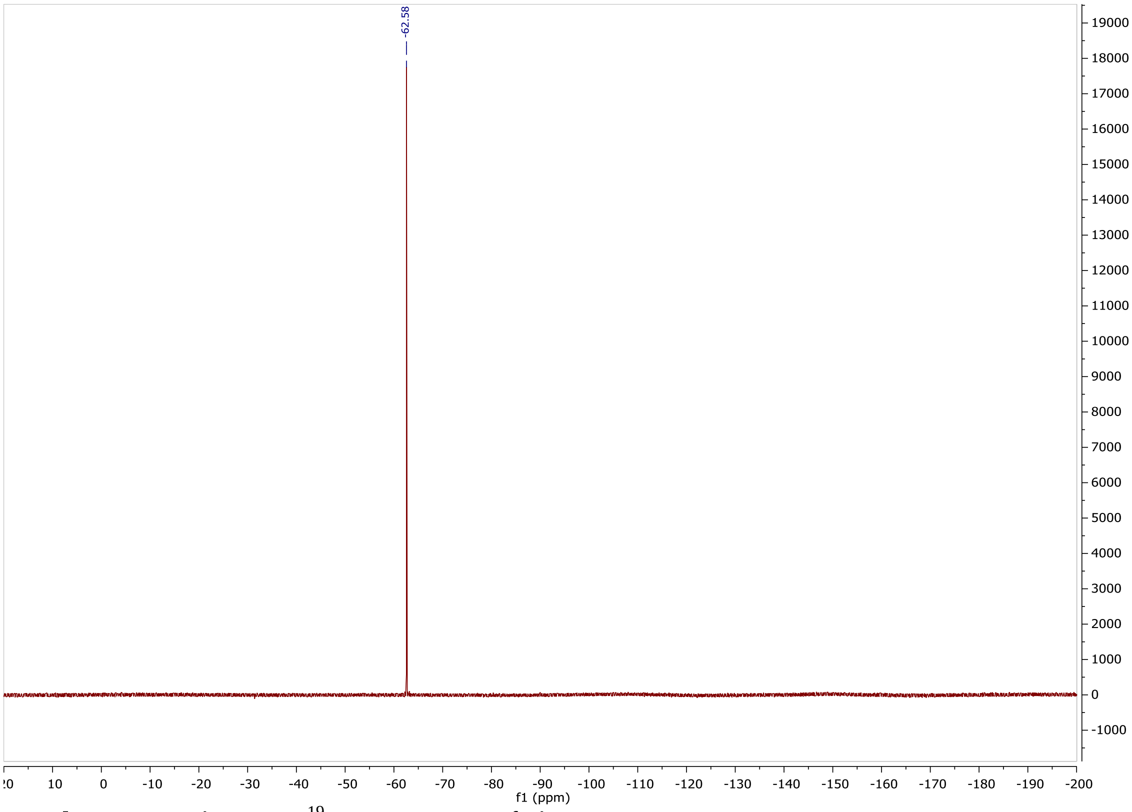

Supplementary Figure 23. ^{19}F NMR spectra of **2i**

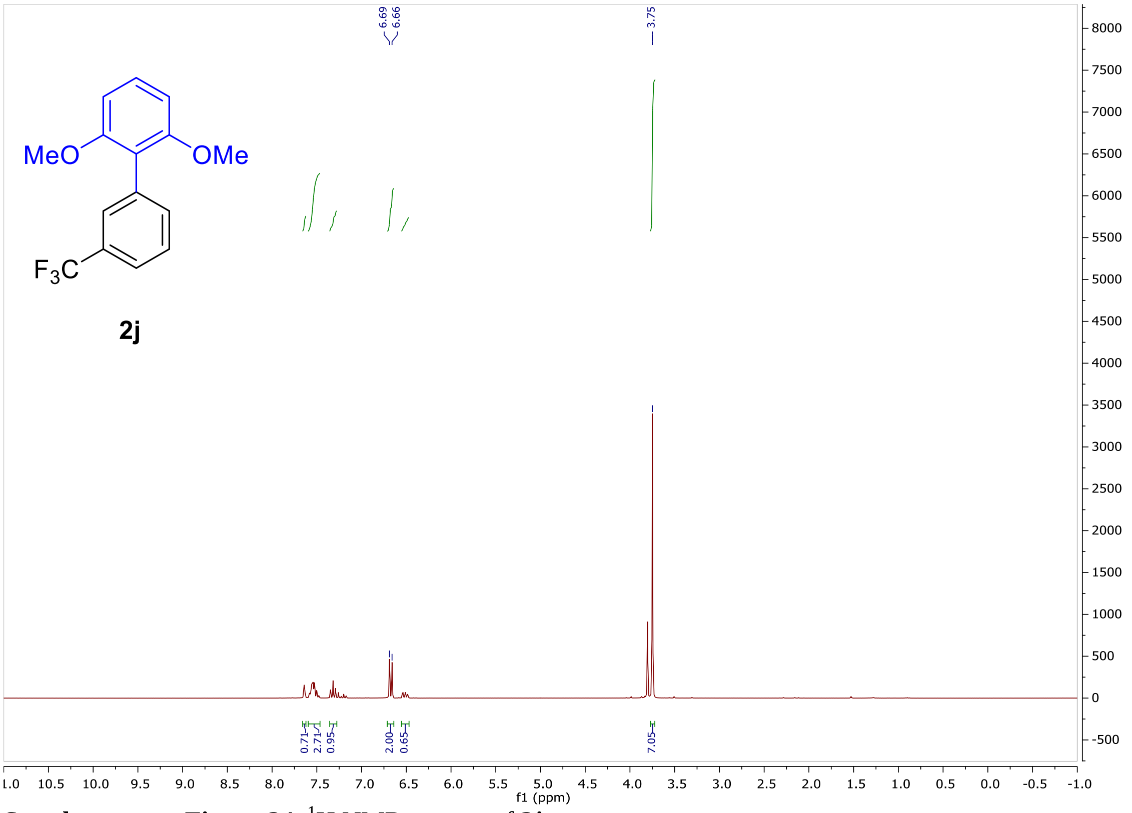

Supplementary Figure 24. ^{1}H NMR spectra of **2j**

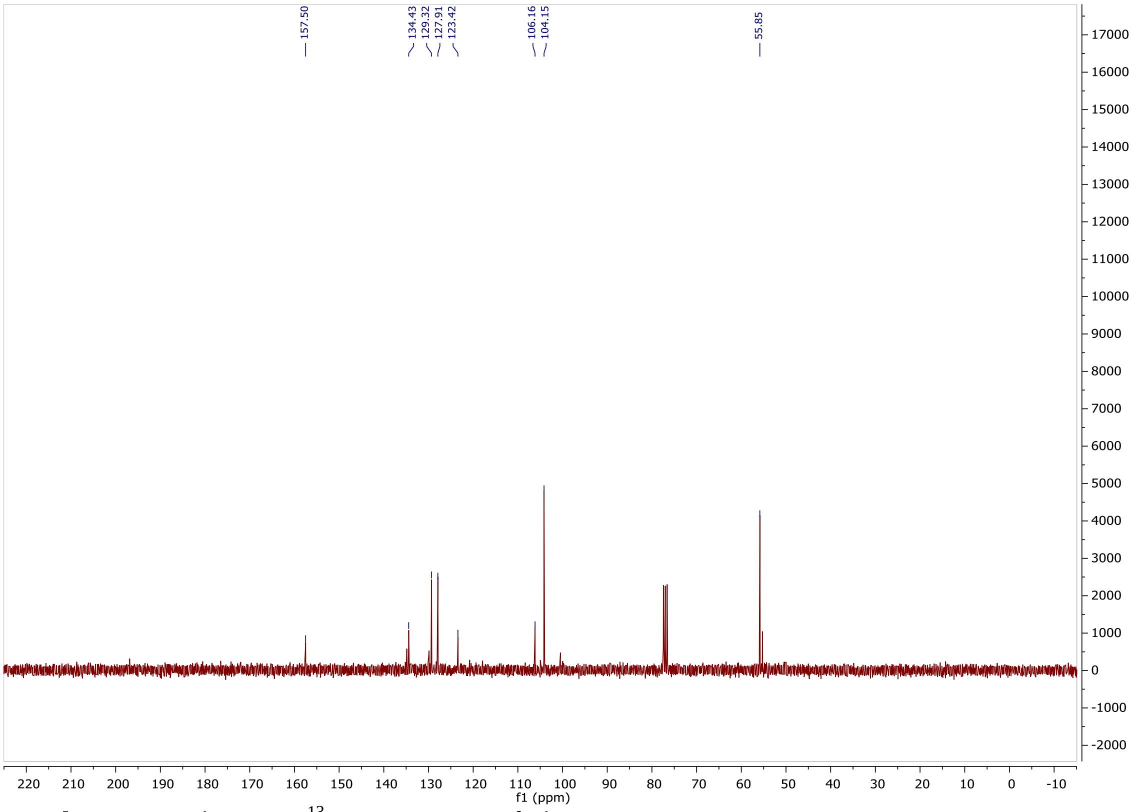

Supplementary Figure 25. ^{13}C NMR spectra of **2j**

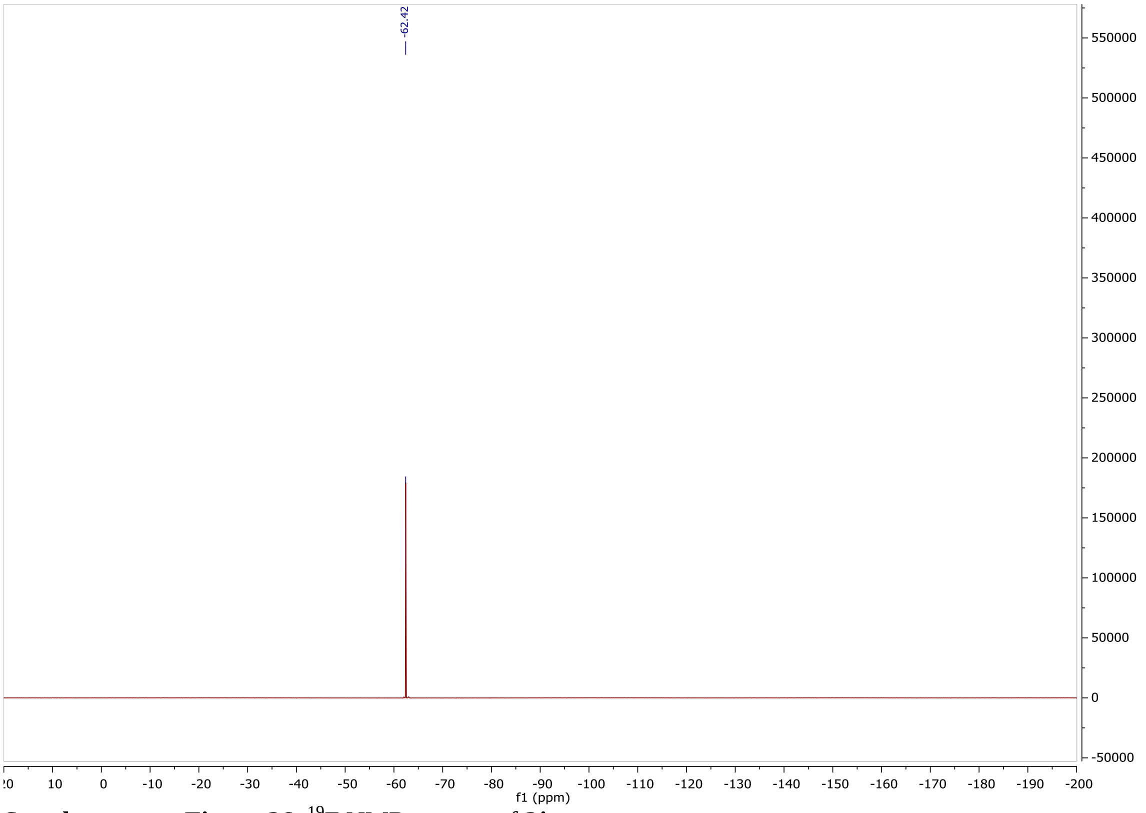

Supplementary Figure 26. ^{19}F NMR spectra of **2j**

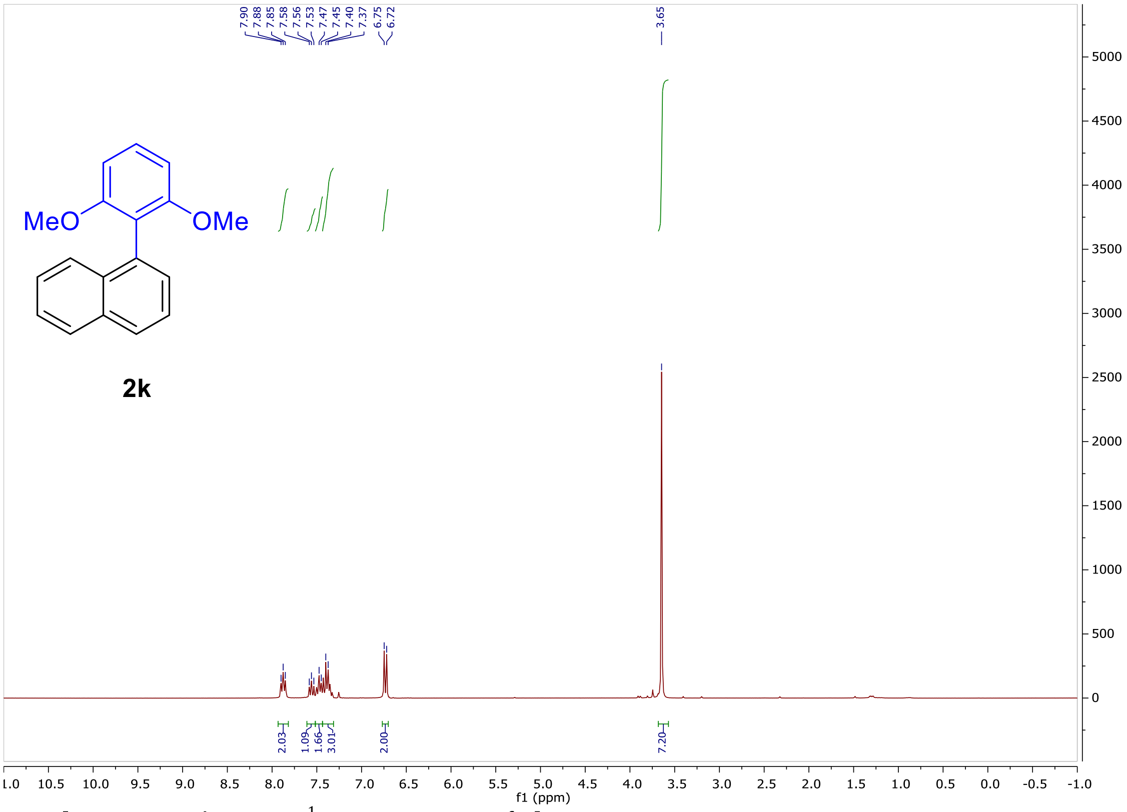

Supplementary Figure 27. ^{1}H NMR spectra of **2k**

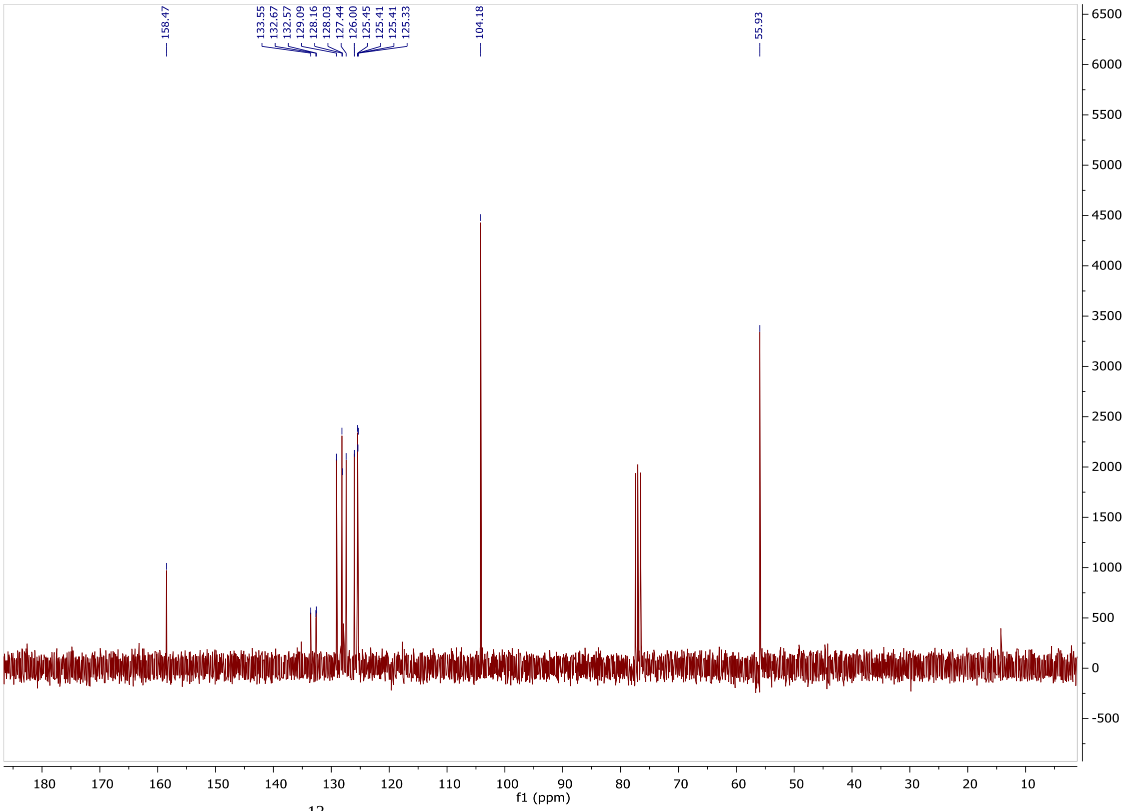

Supplementary Figure 28. ^{13}C NMR spectra of **2k**

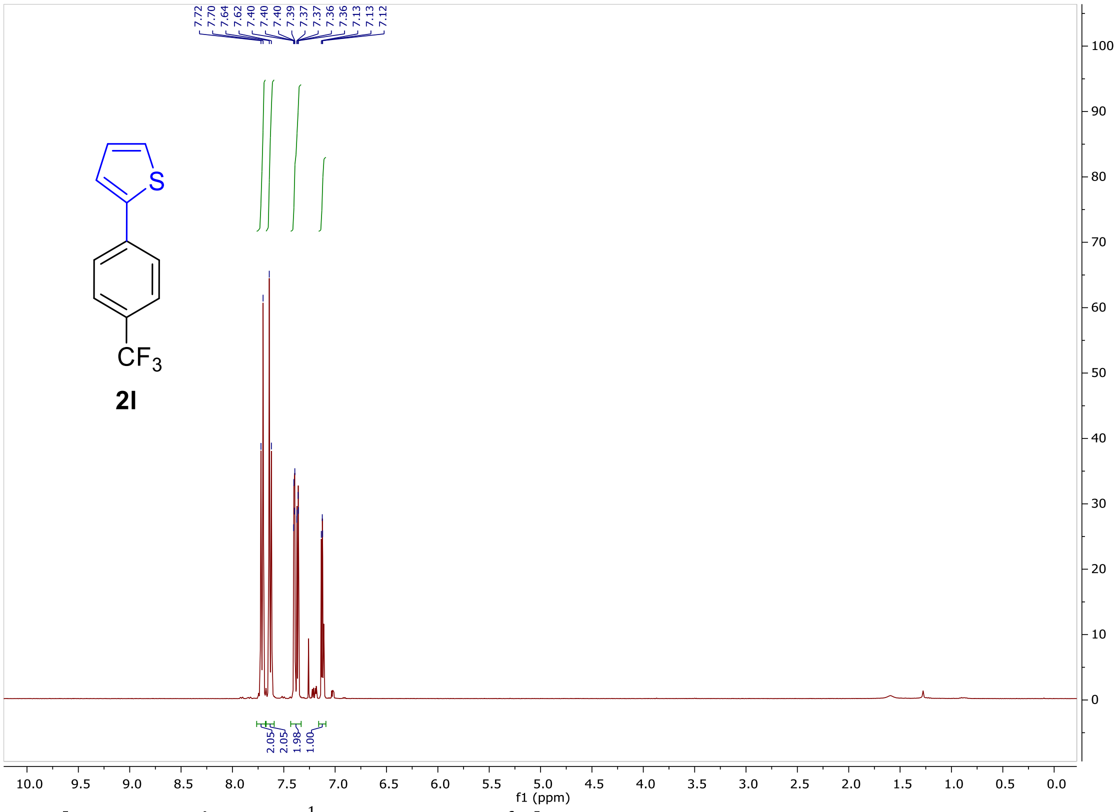

Supplementary Figure 29. ^{1}H NMR spectra of **2l**

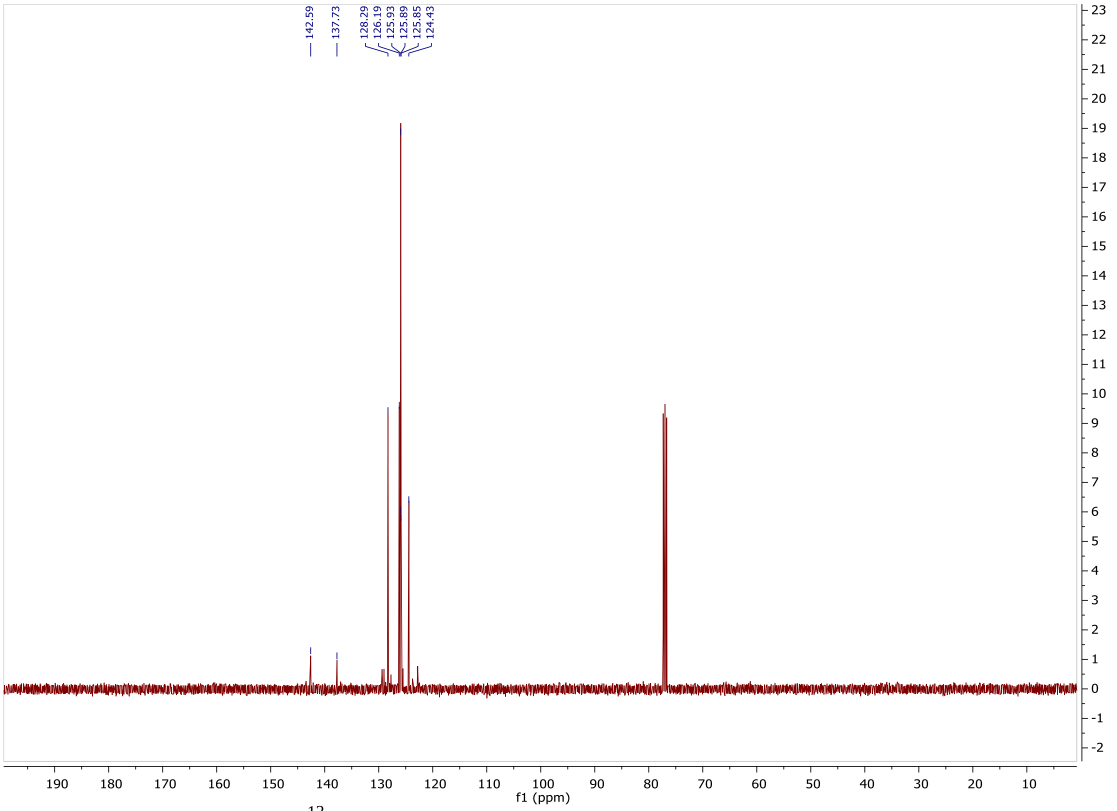

Supplementary Figure 30. ^{13}C NMR spectra of **2l**

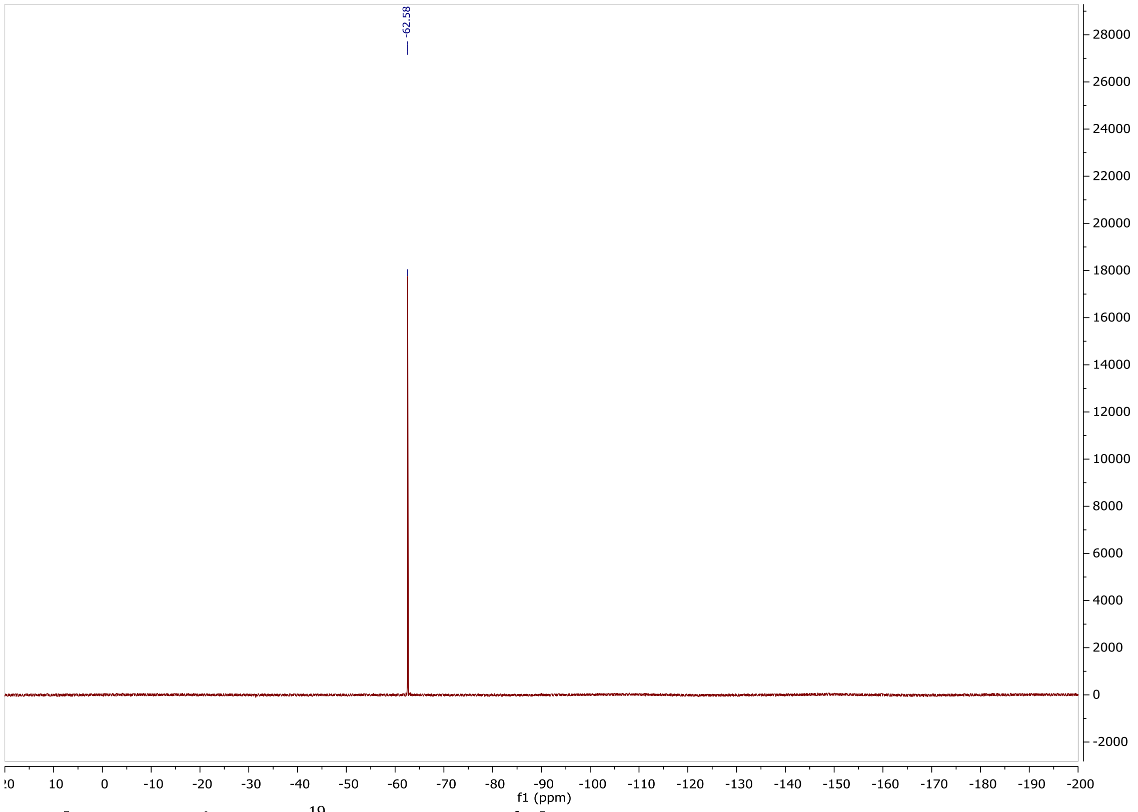

Supplementary Figure 31. ^{19}F NMR spectra of **2l**

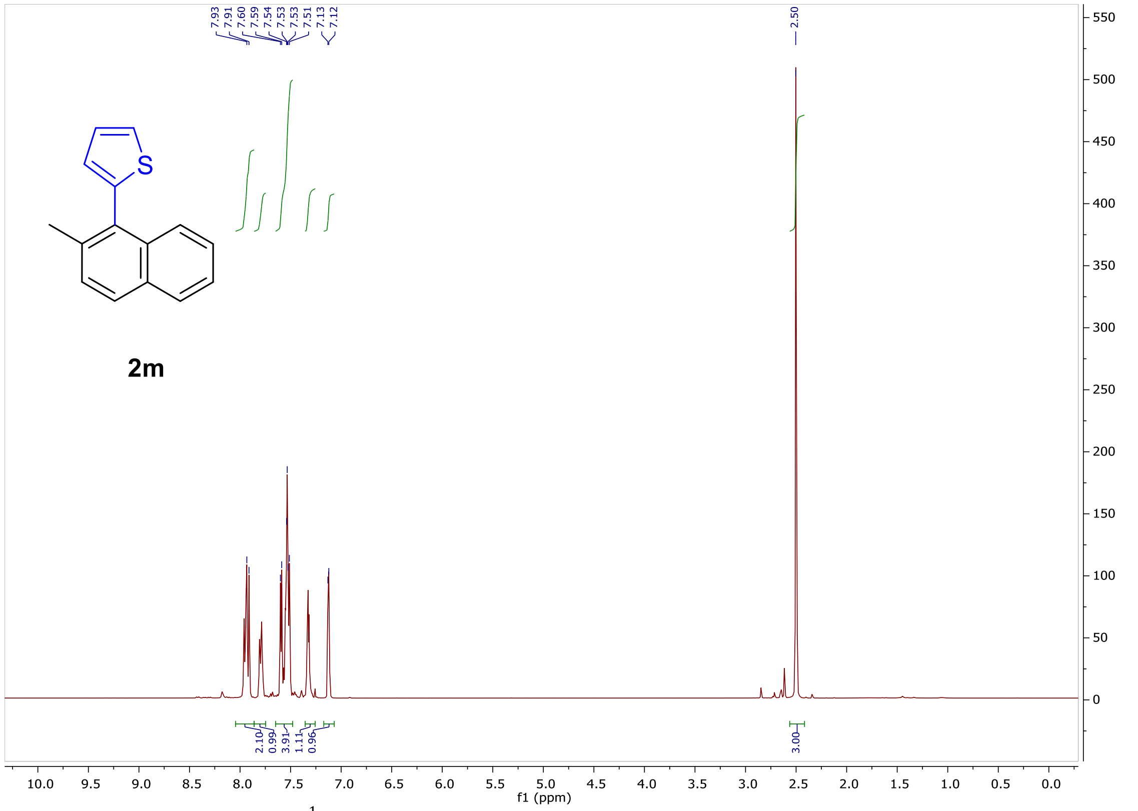

Supplementary Figure 32. ^{1}H NMR spectra of **2m**

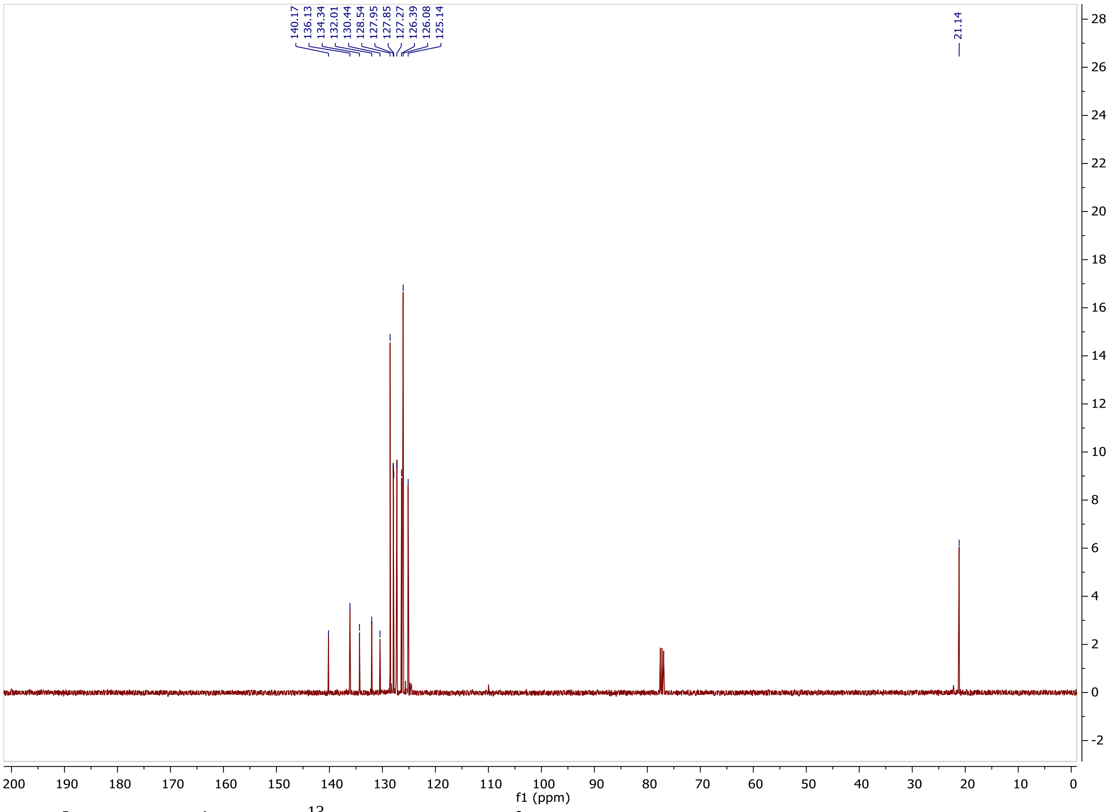

Supplementary Figure 33. ^{13}C NMR spectra of **2m**

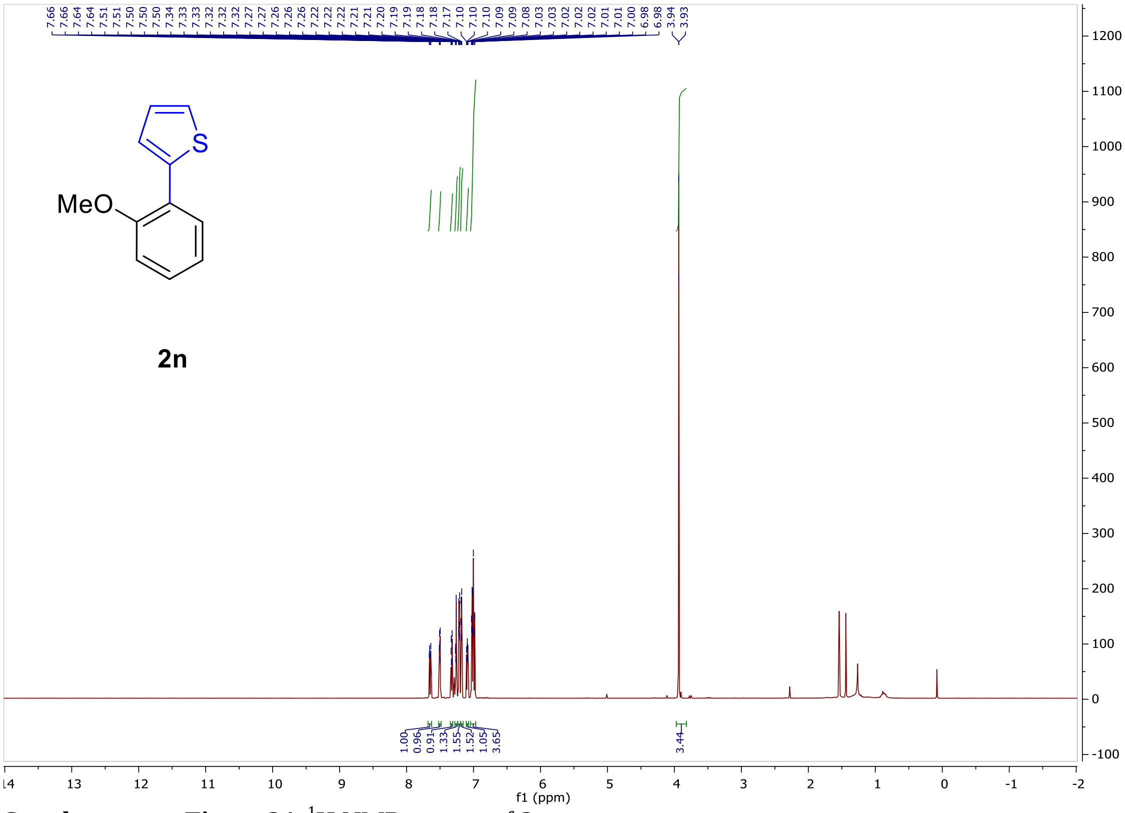

Supplementary Figure 34. ^{1}H NMR spectra of **2n**

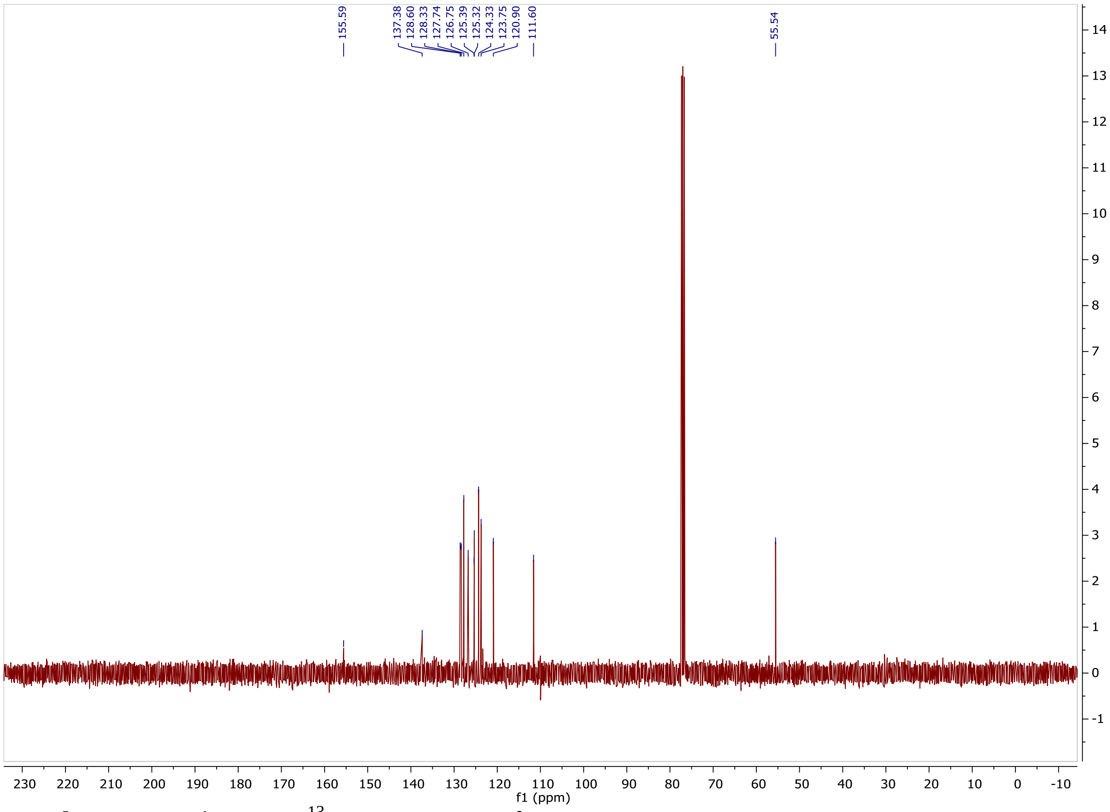

Supplementary Figure 35. ^{13}C NMR spectra of **2n**

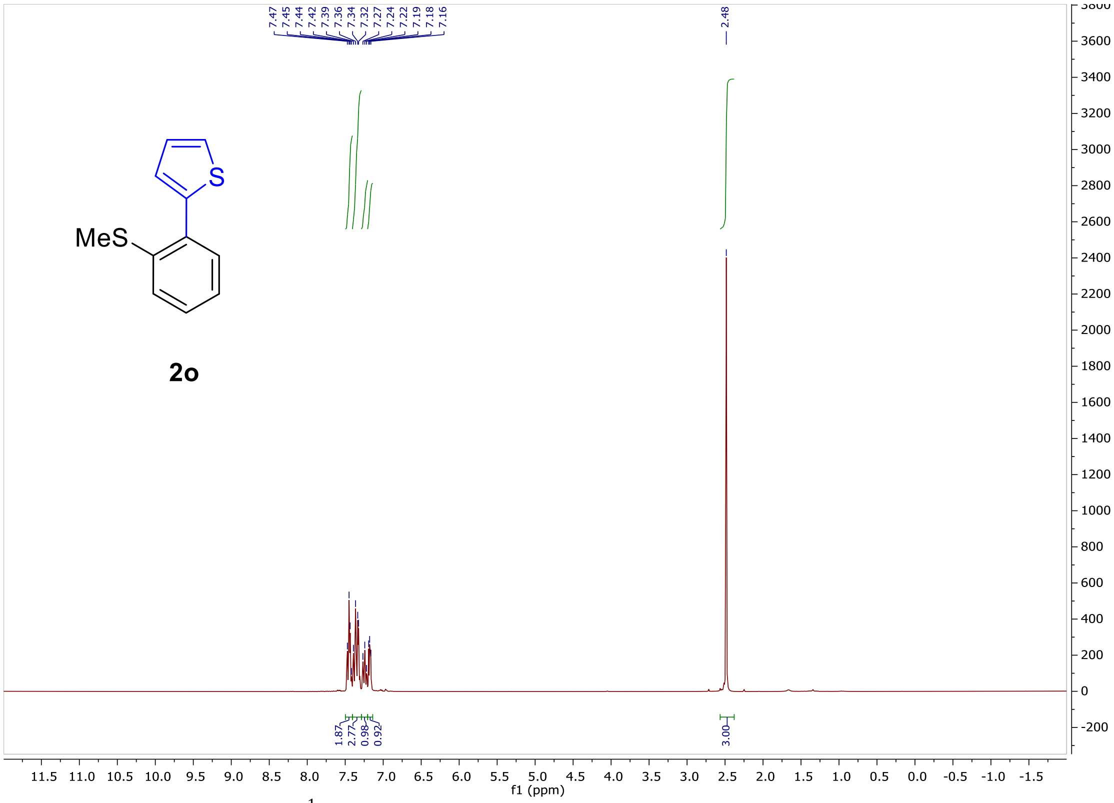

Supplementary Figure 36. ^{1}H NMR spectra of **2o**

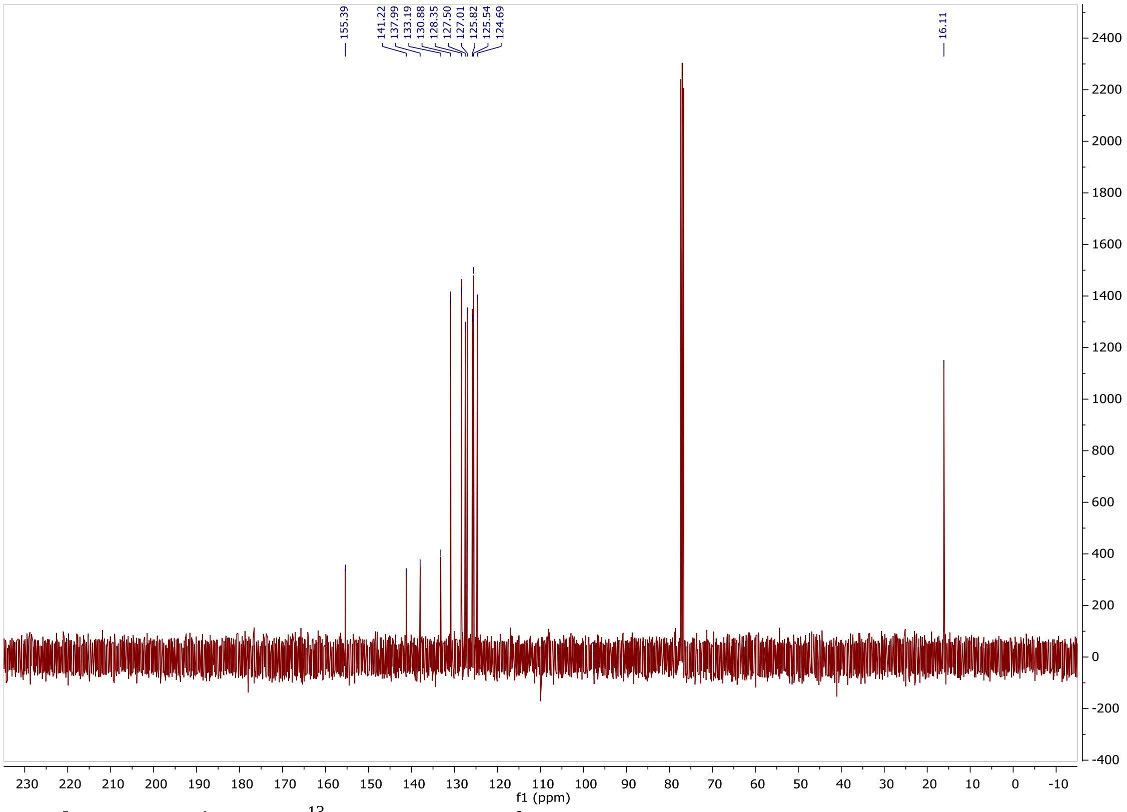

Supplementary Figure 37. ^{13}C NMR spectra of **2o**

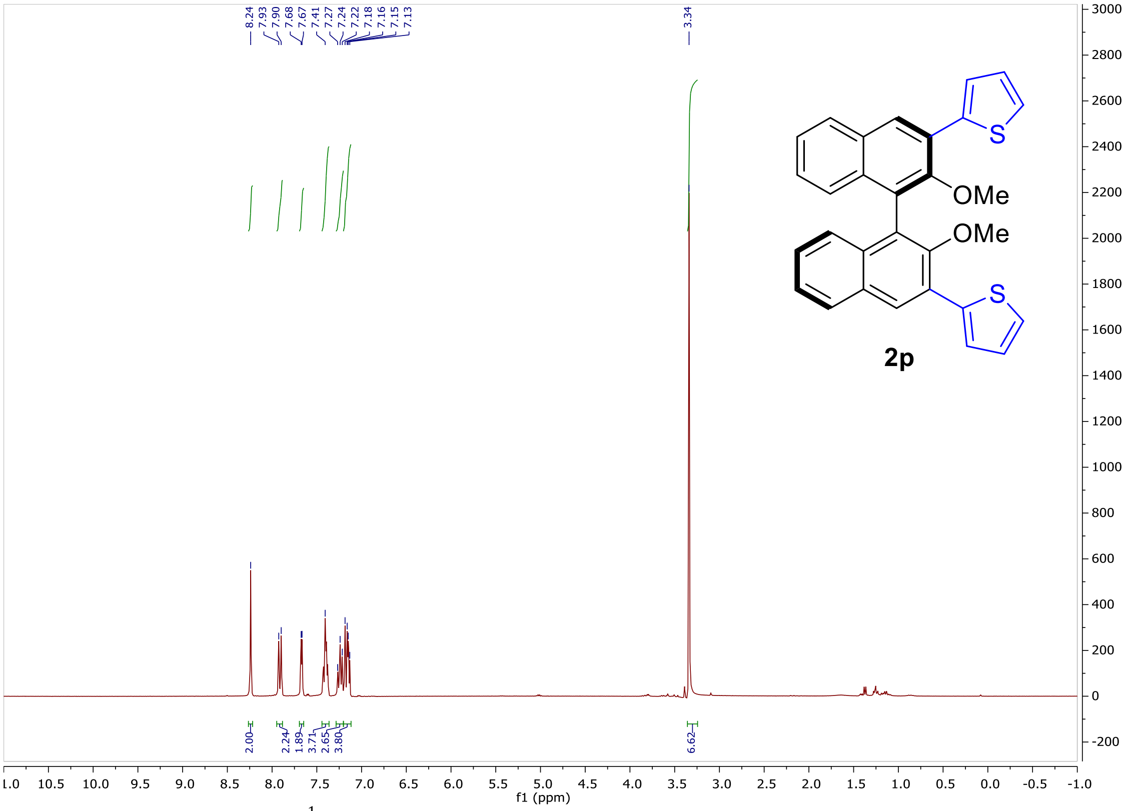

Supplementary Figure 38. ^{1}H NMR spectra of **2p**

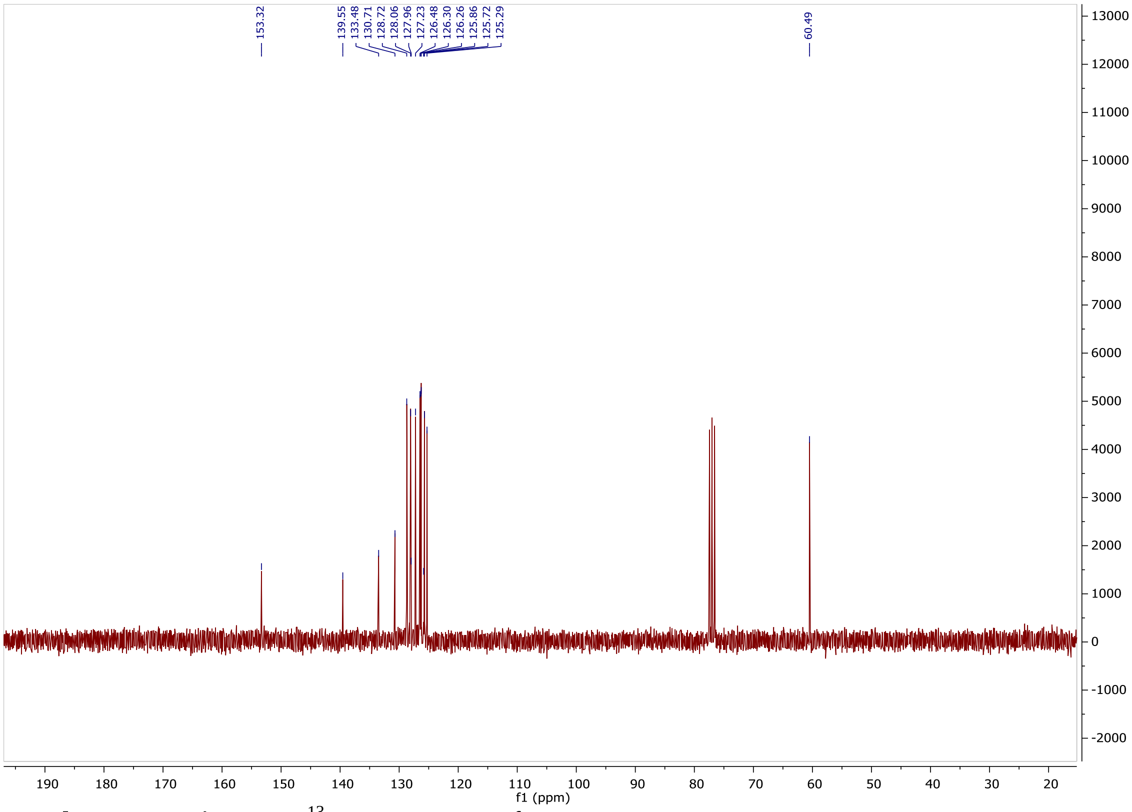

Supplementary Figure 39. ^{13}C NMR spectra of **2p**

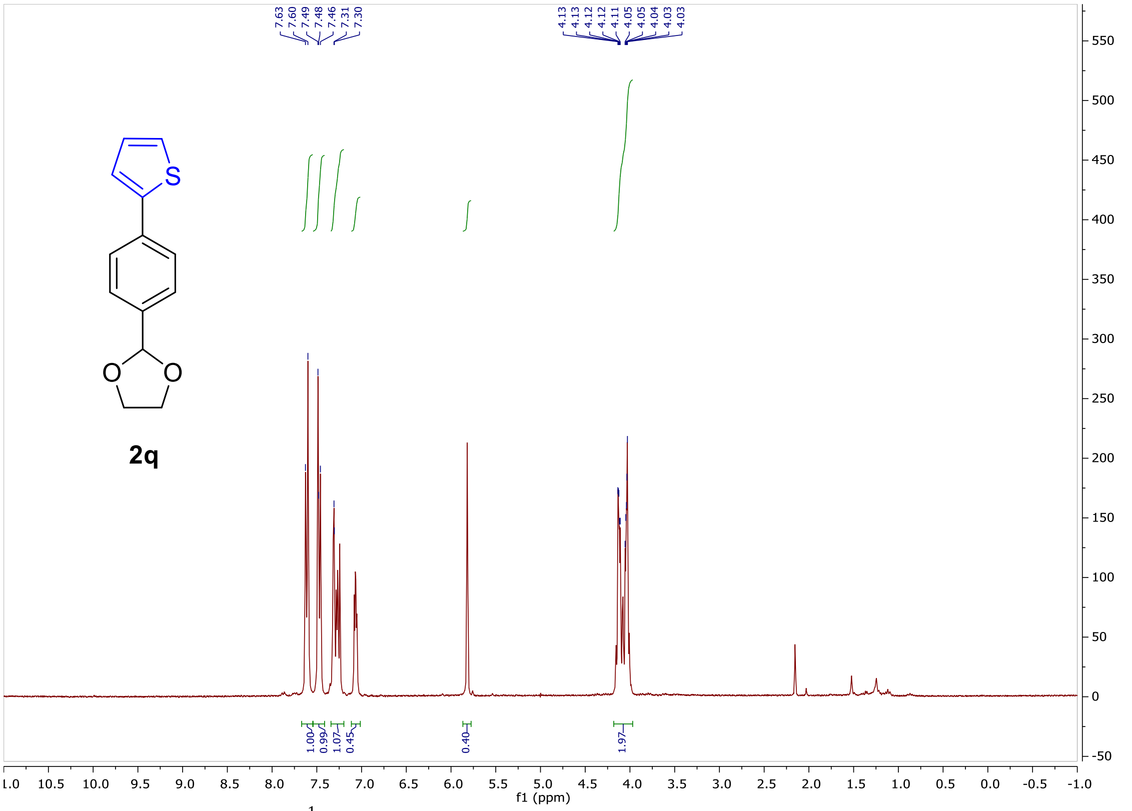

Supplementary Figure 40. ^{1}H NMR spectra of **2q**

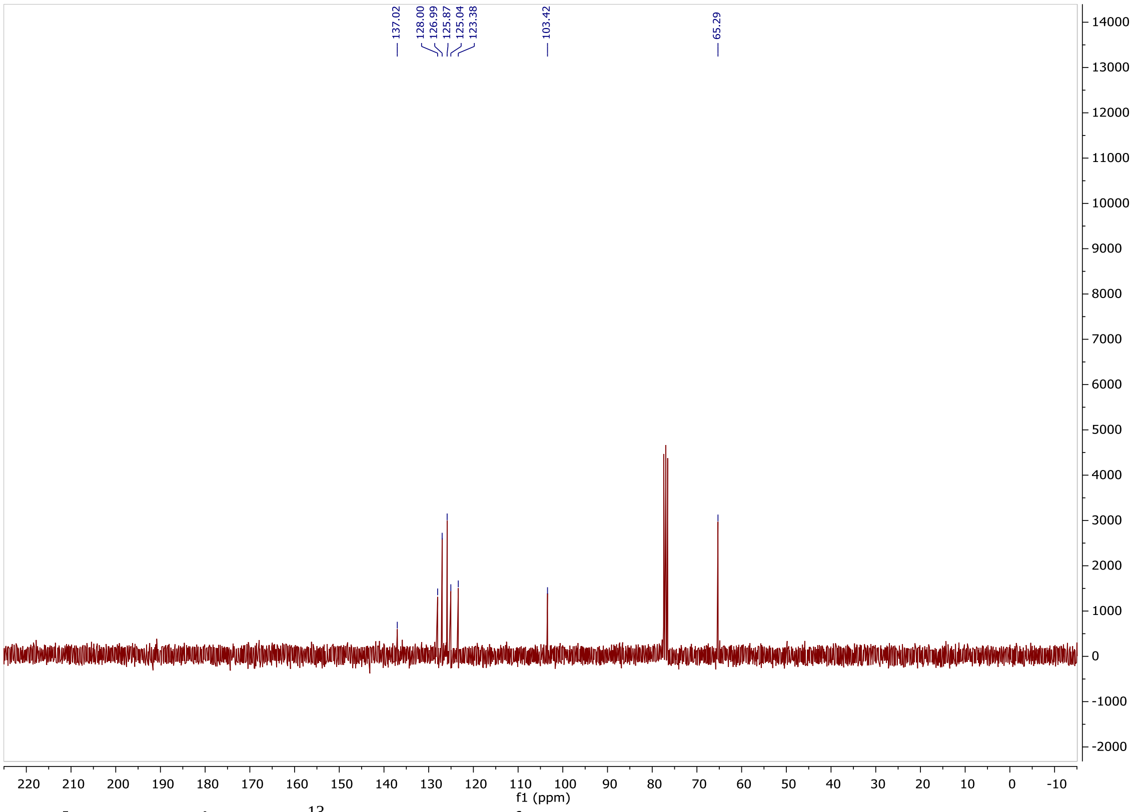

Supplementary Figure 41. ^{13}C NMR spectra of **2q**

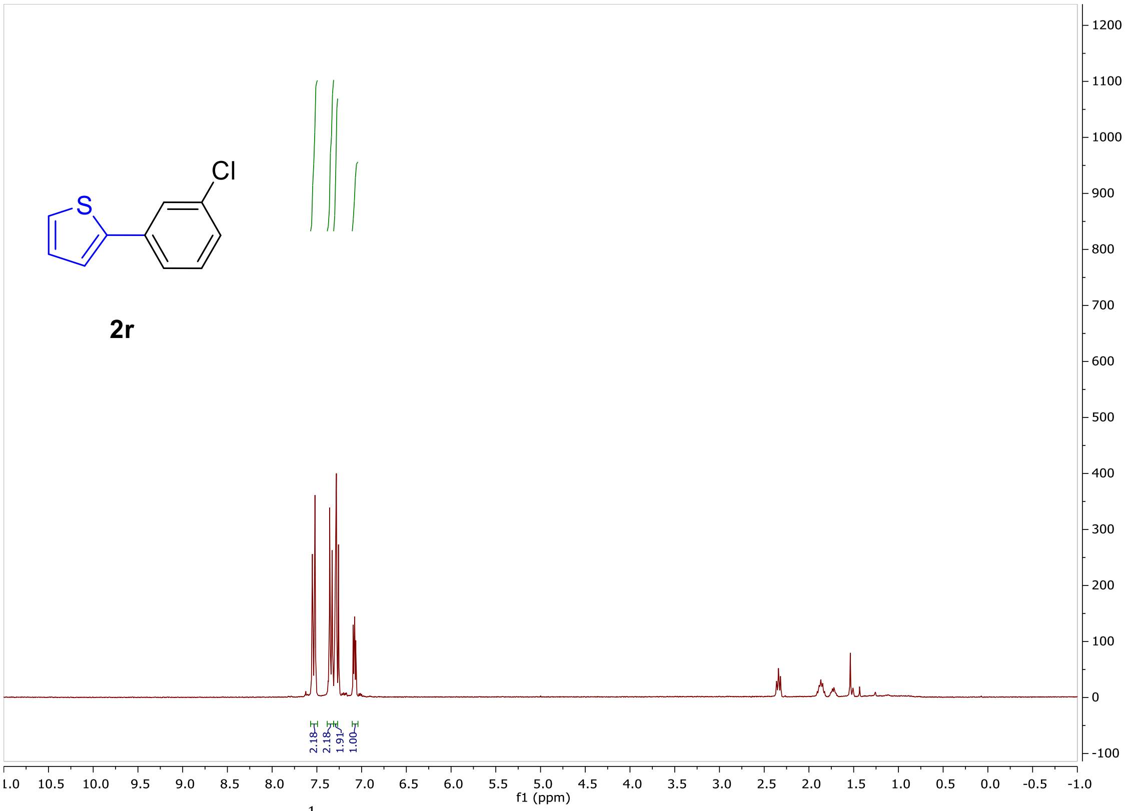

Supplementary Figure 42. ^{1}H NMR spectra of **2r**

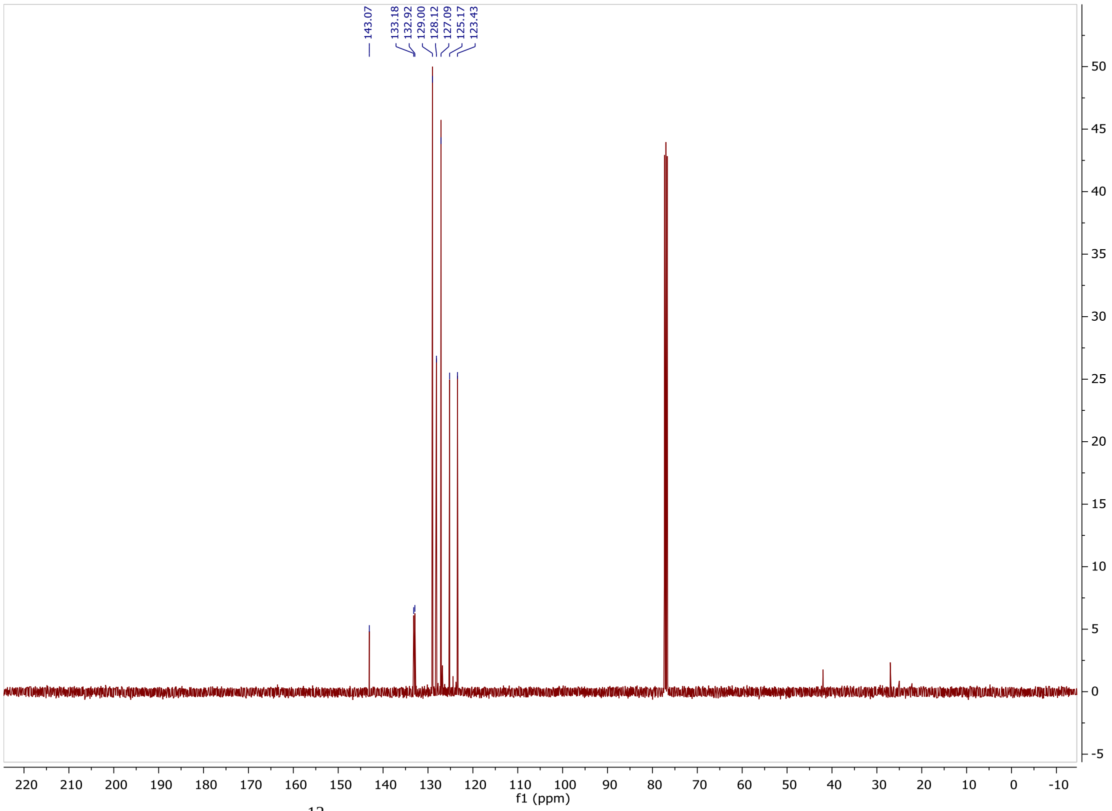

Supplementary Figure 43. ^{13}C NMR spectra of **2r**

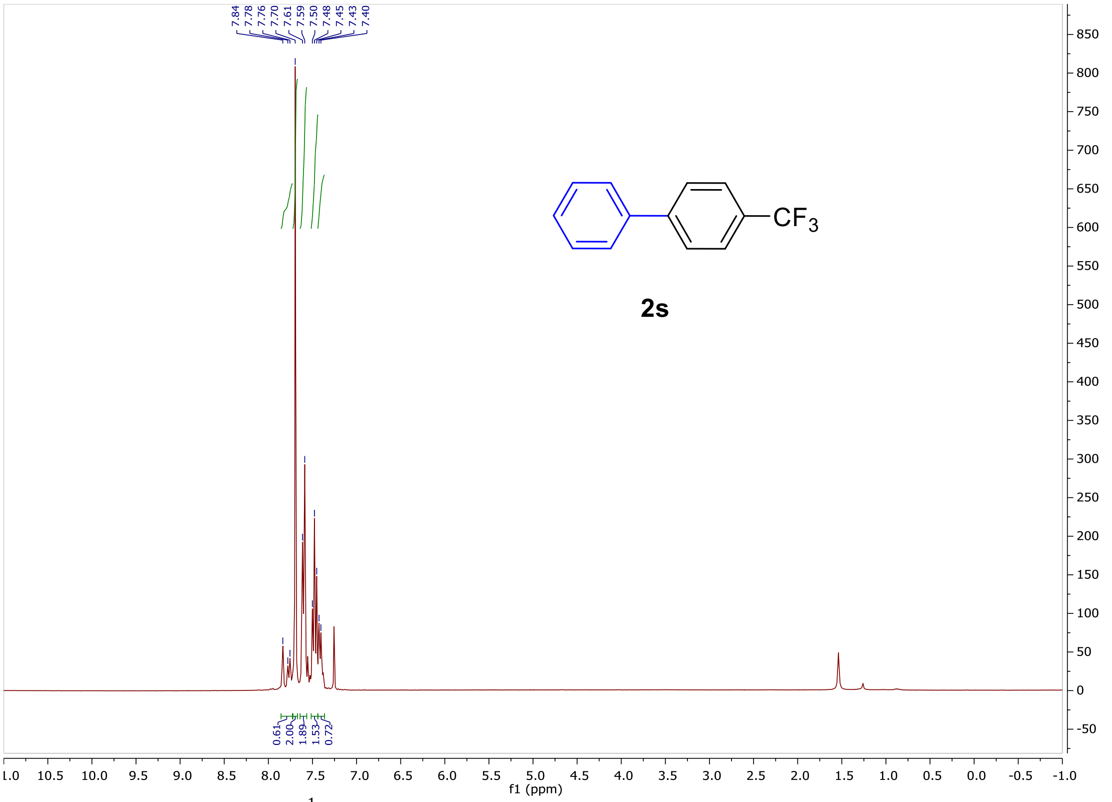

Supplementary Figure 44. ^{1}H NMR spectra of **2s**

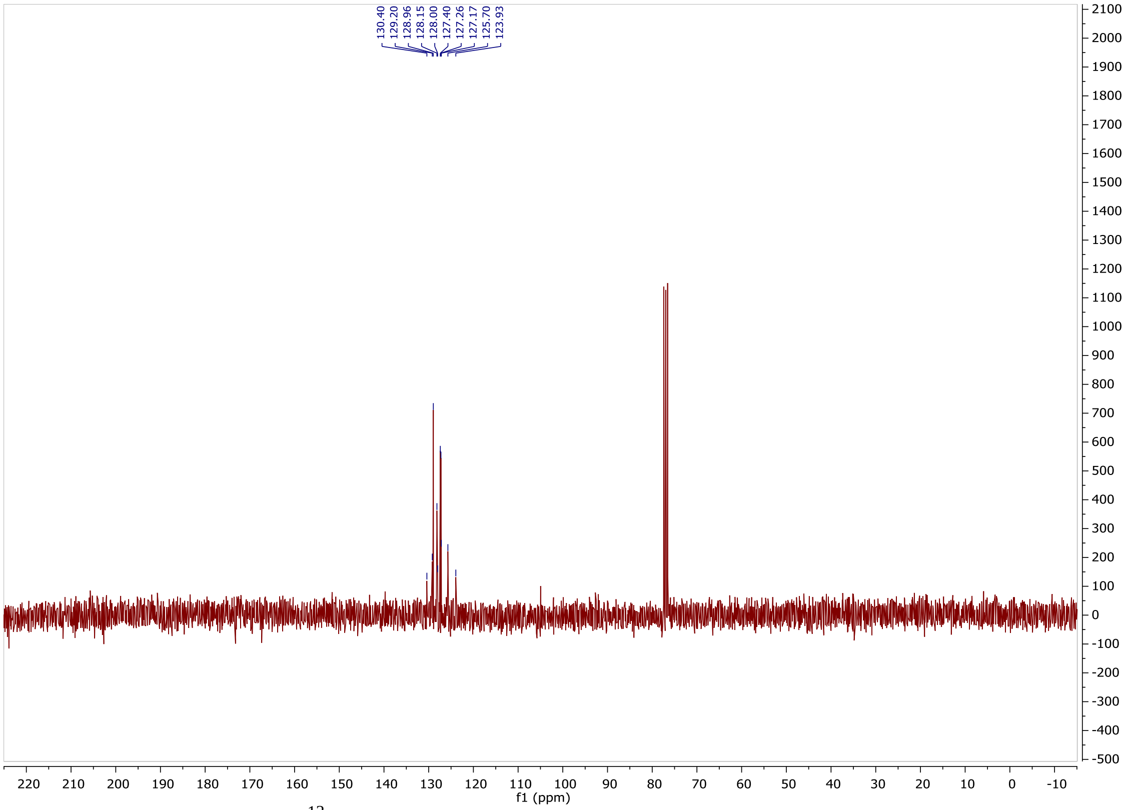

Supplementary Figure 45. ^{13}C NMR spectra of **2s**

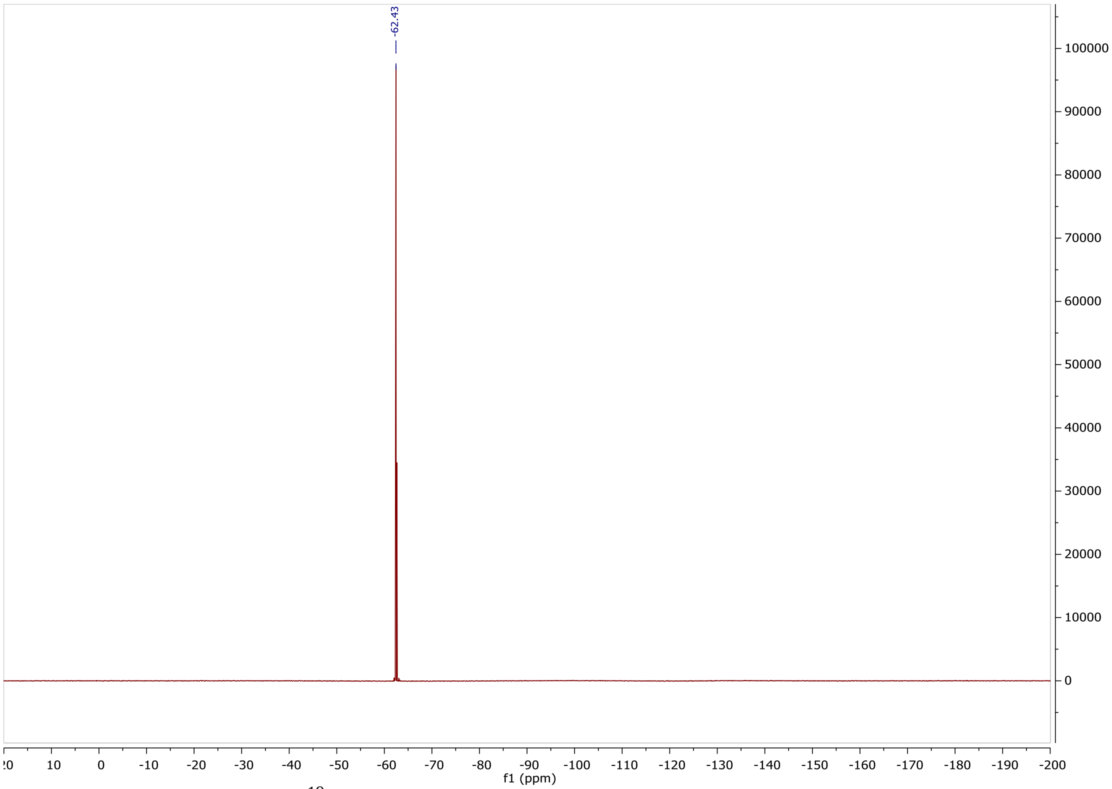

Supplementary Figure 46. ^{19}F NMR spectra of **2s**

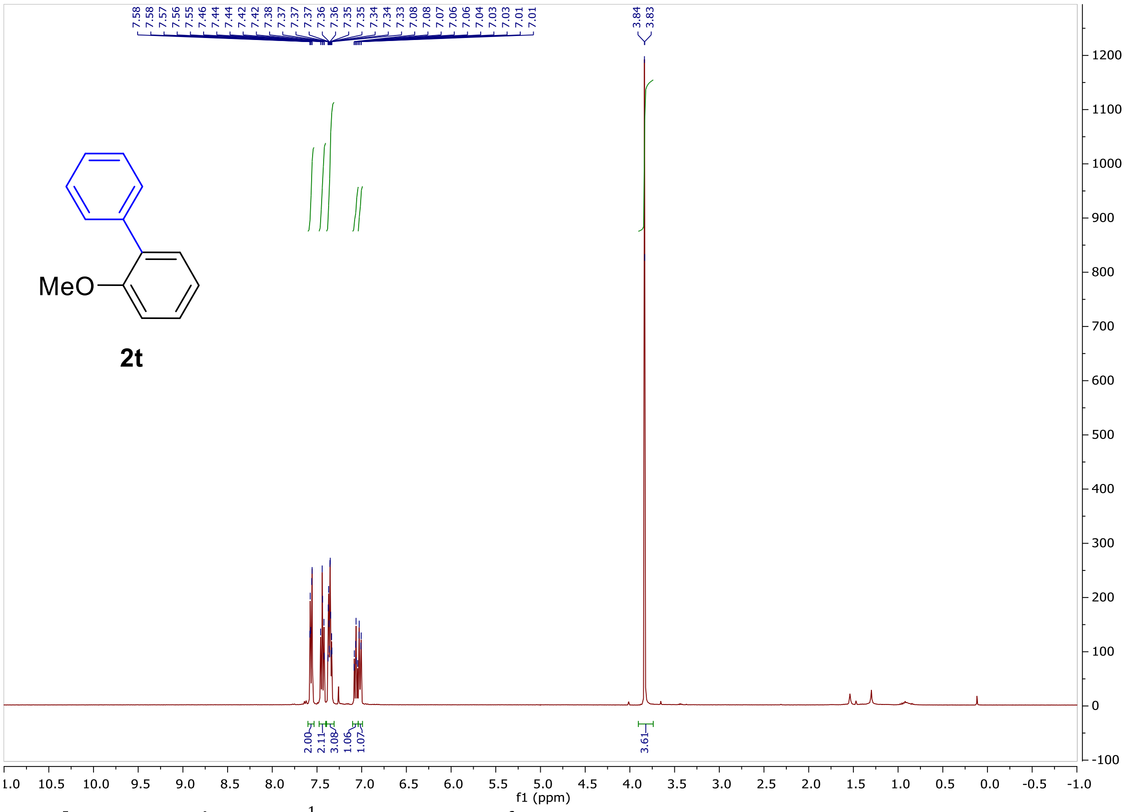

Supplementary Figure 47. ^{1}H NMR spectra of **2t**

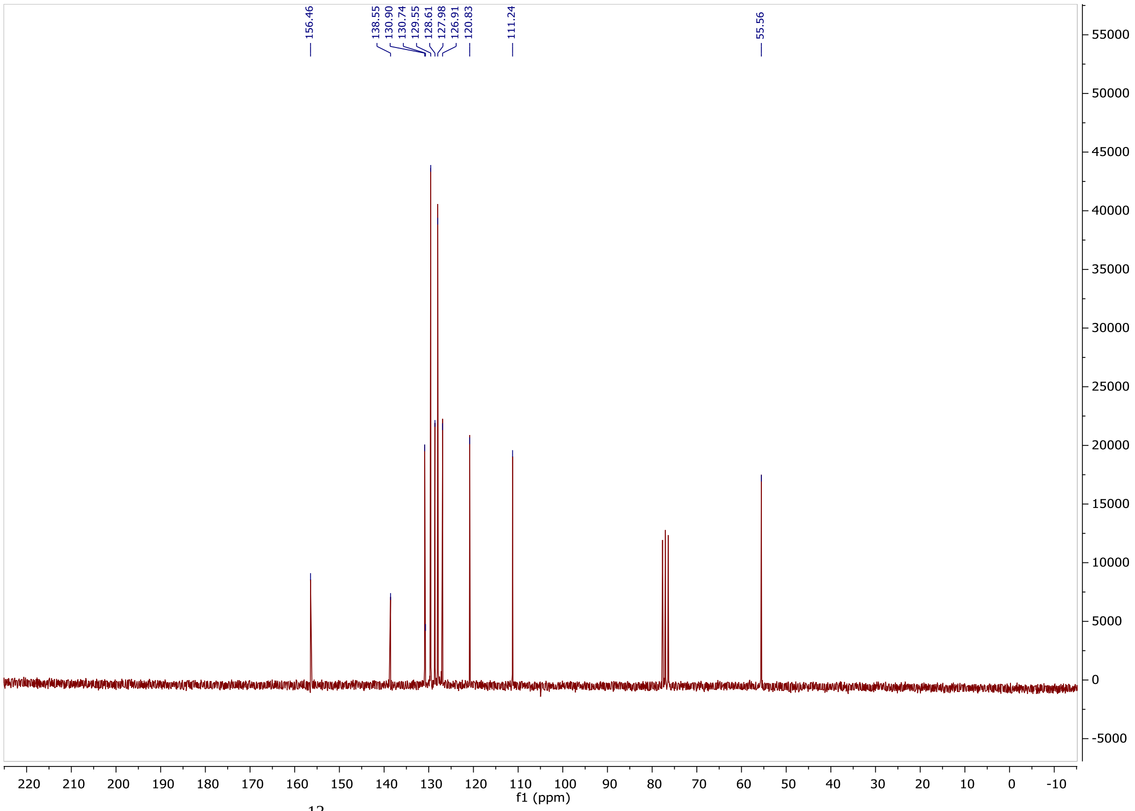

Supplementary Figure 48. ^{13}C NMR spectra of **2t**

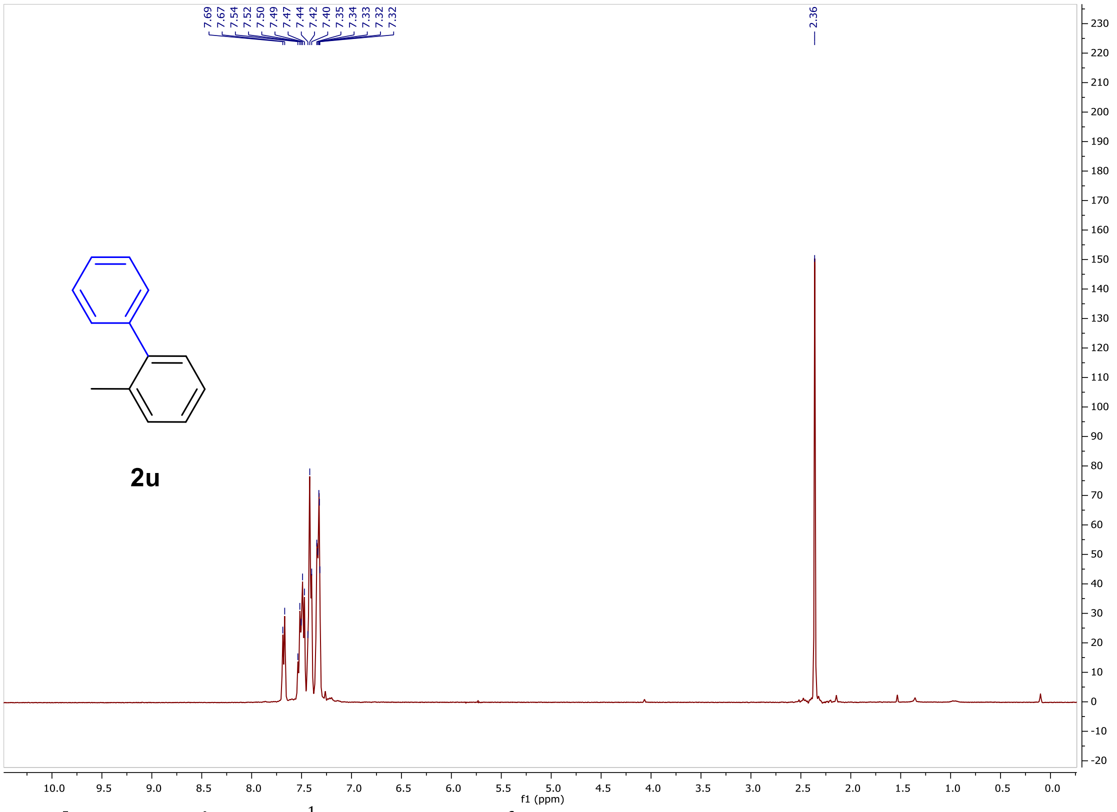

Supplementary Figure 49. ^{1}H NMR spectra of **2u**

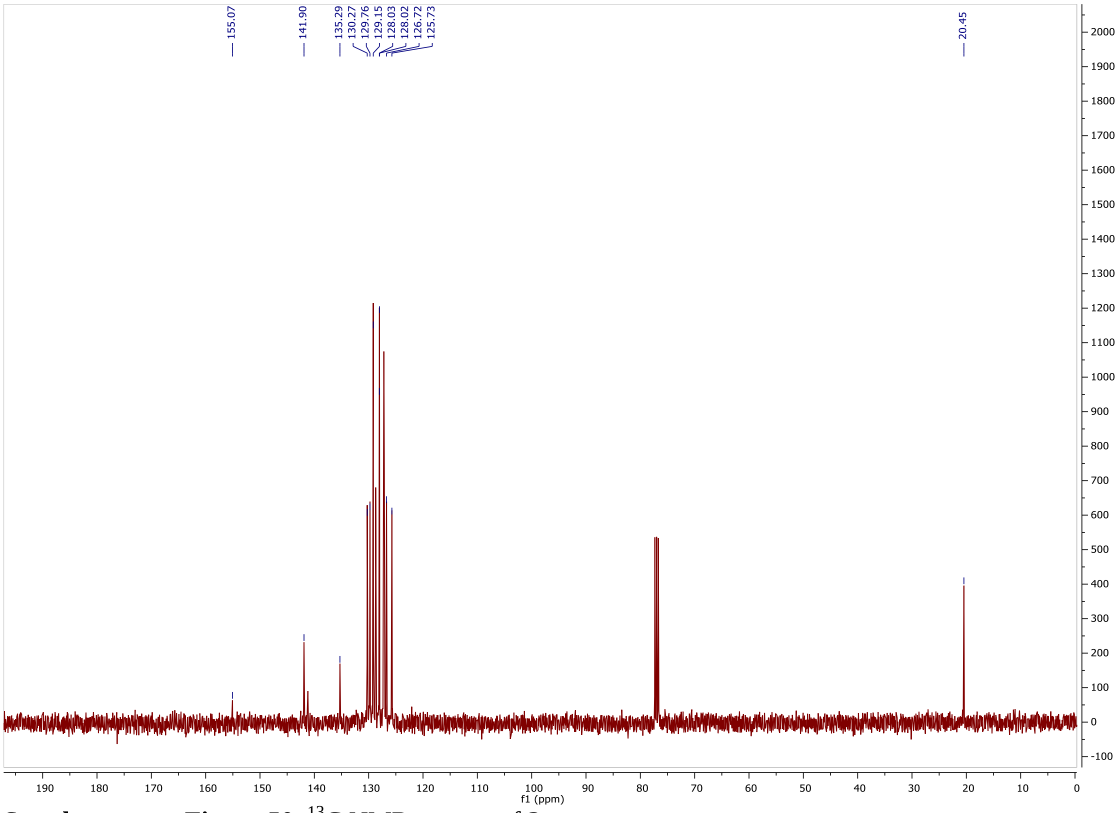

Supplementary Figure 50. ^{13}C NMR spectra of **2u**

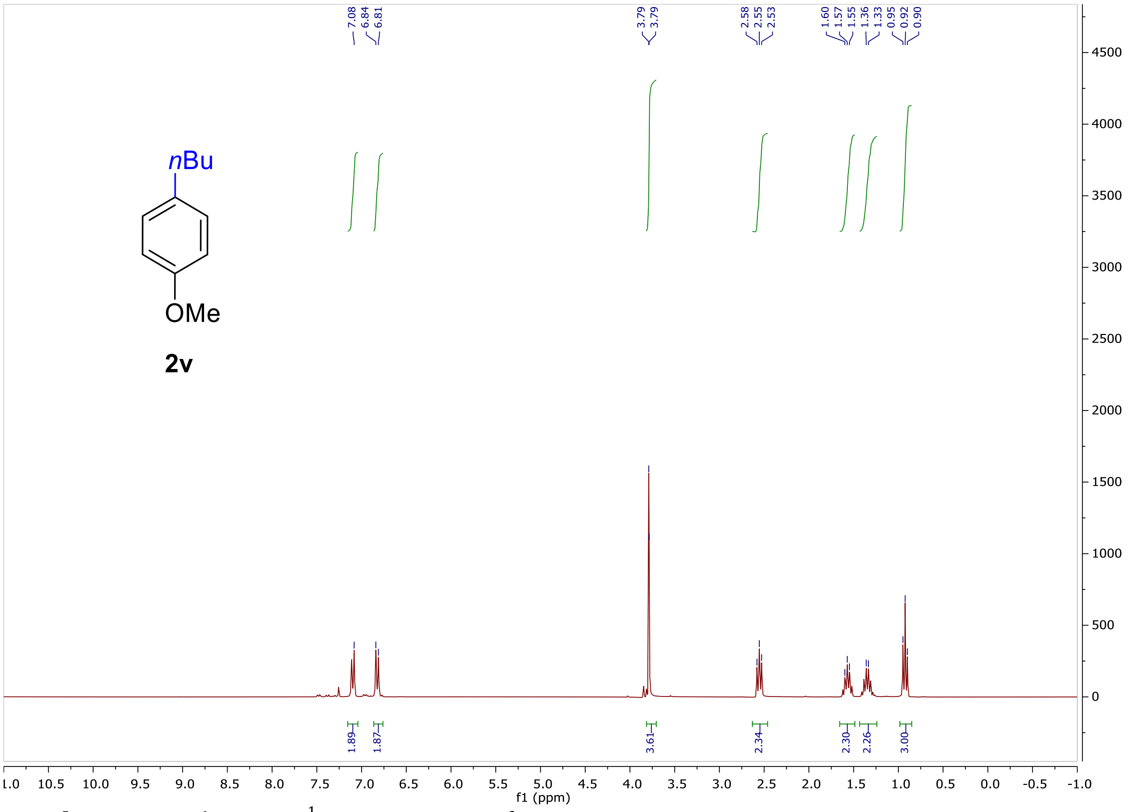

Supplementary Figure 51. ^{1}H NMR spectra of **2v**

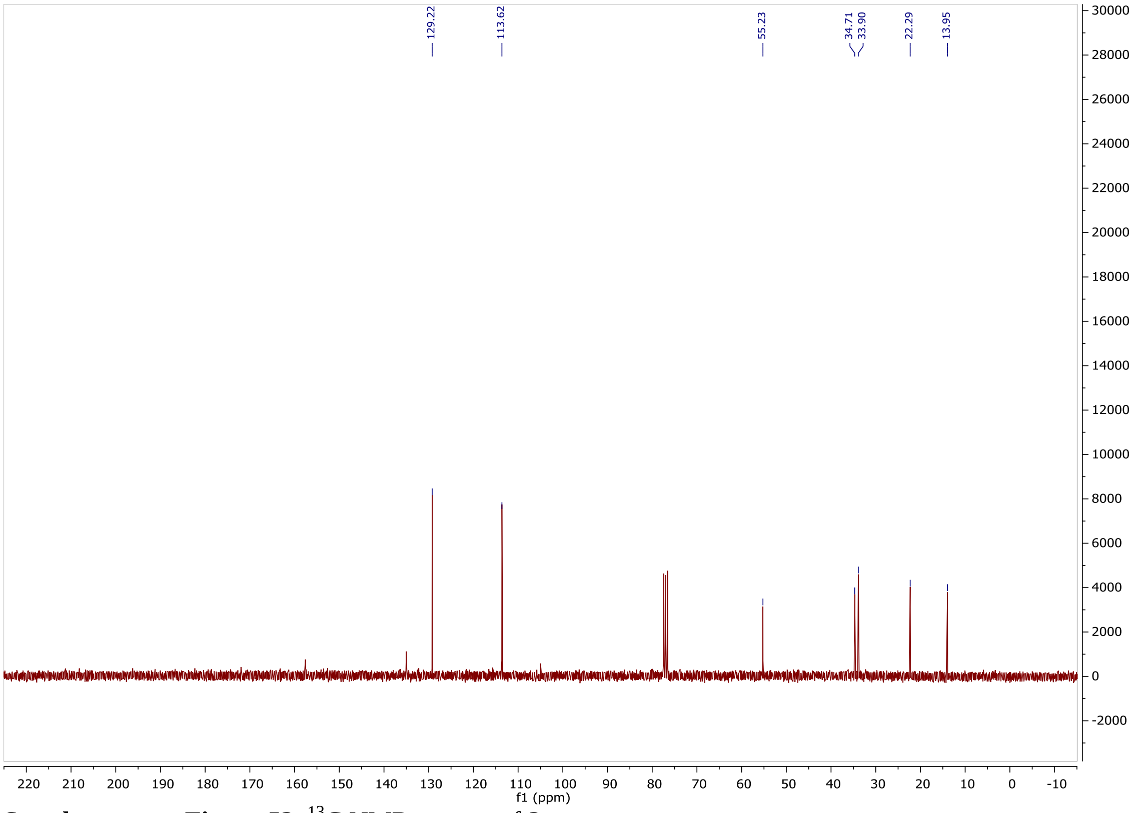

Supplementary Figure 52. ^{13}C NMR spectra of **2v**

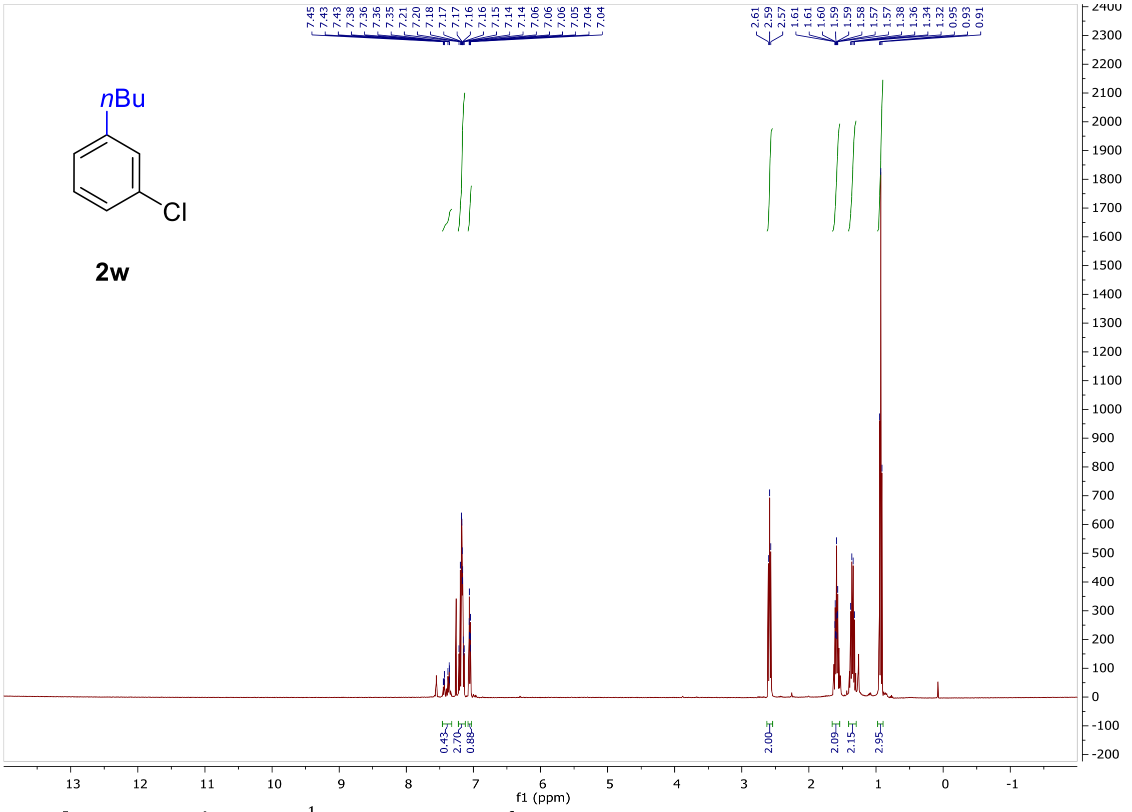

Supplementary Figure 53. ^{1}H NMR spectra of **2w**

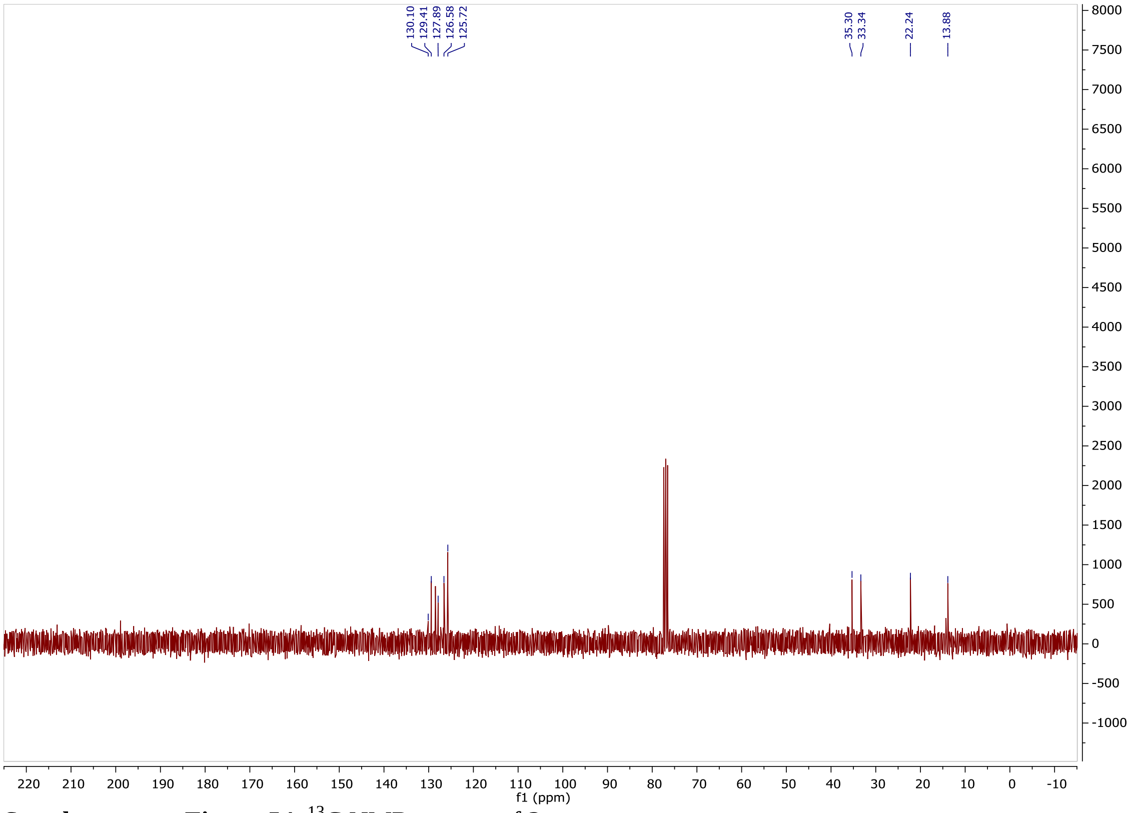

Supplementary Figure 54. ^{13}C NMR spectra of **2w**

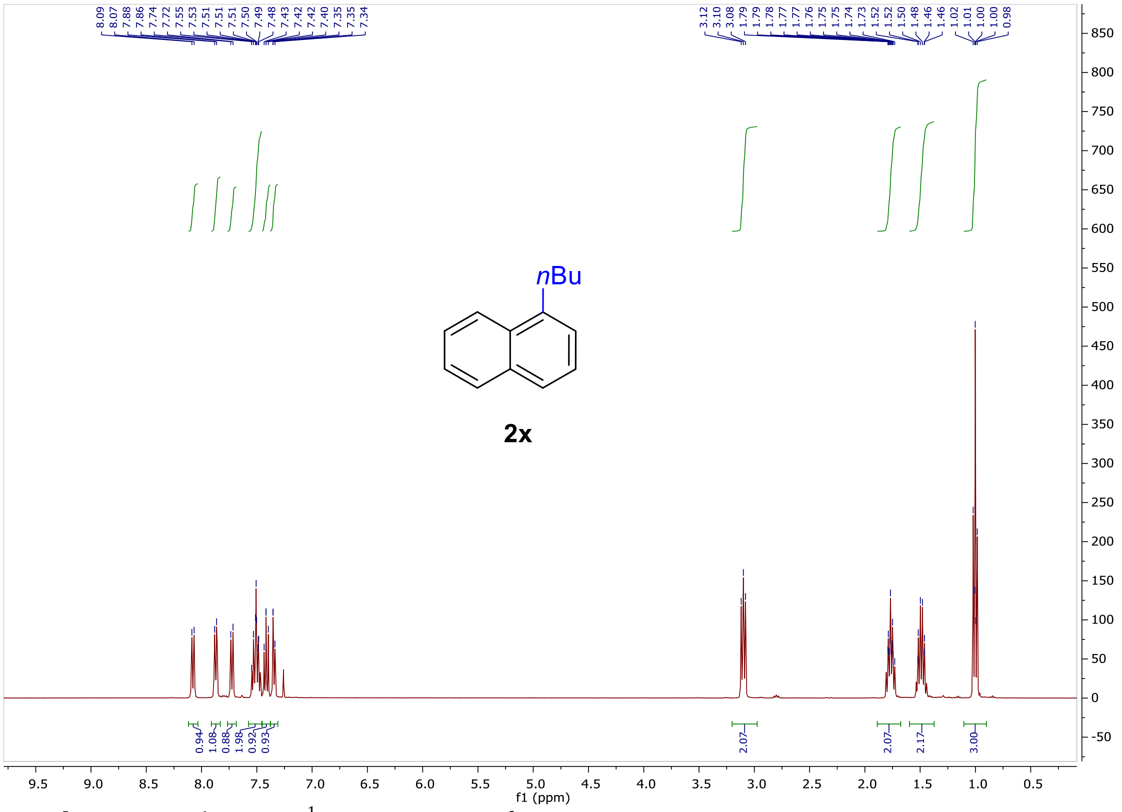

Supplementary Figure 55. ^{1}H NMR spectra of **2x**

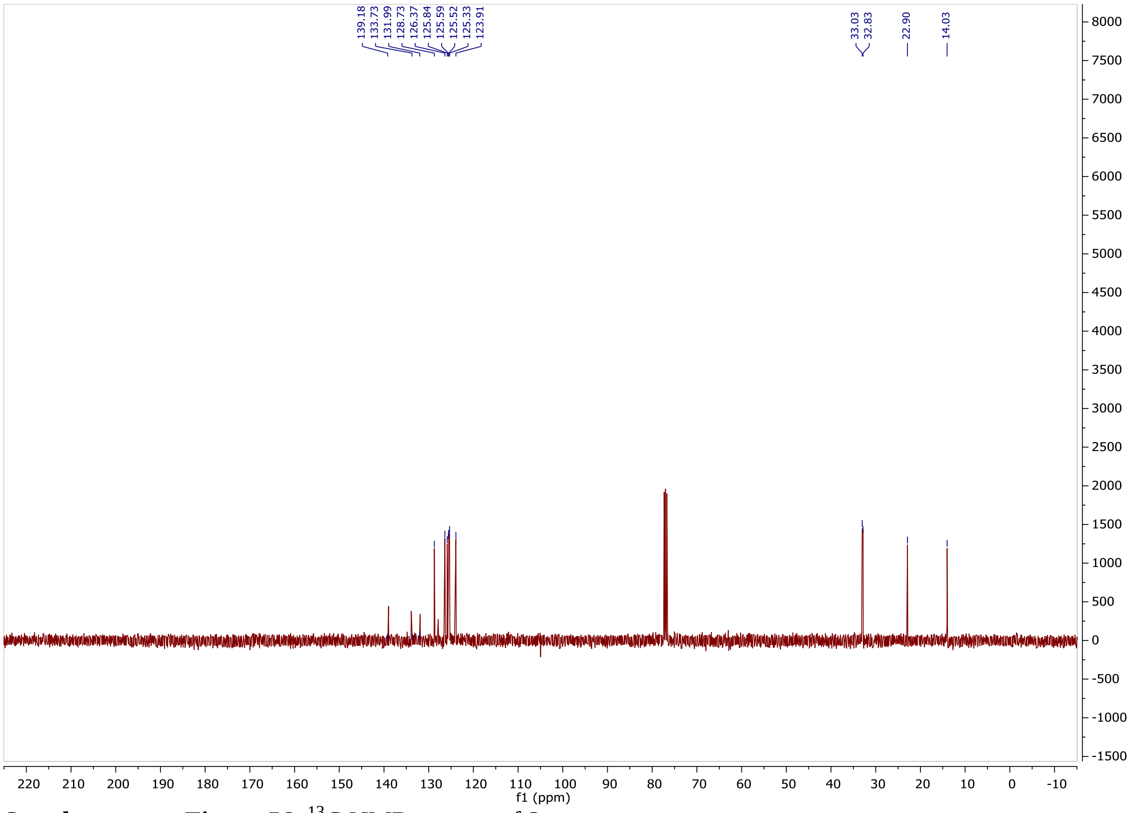

Supplementary Figure 56. ^{13}C NMR spectra of **2x**

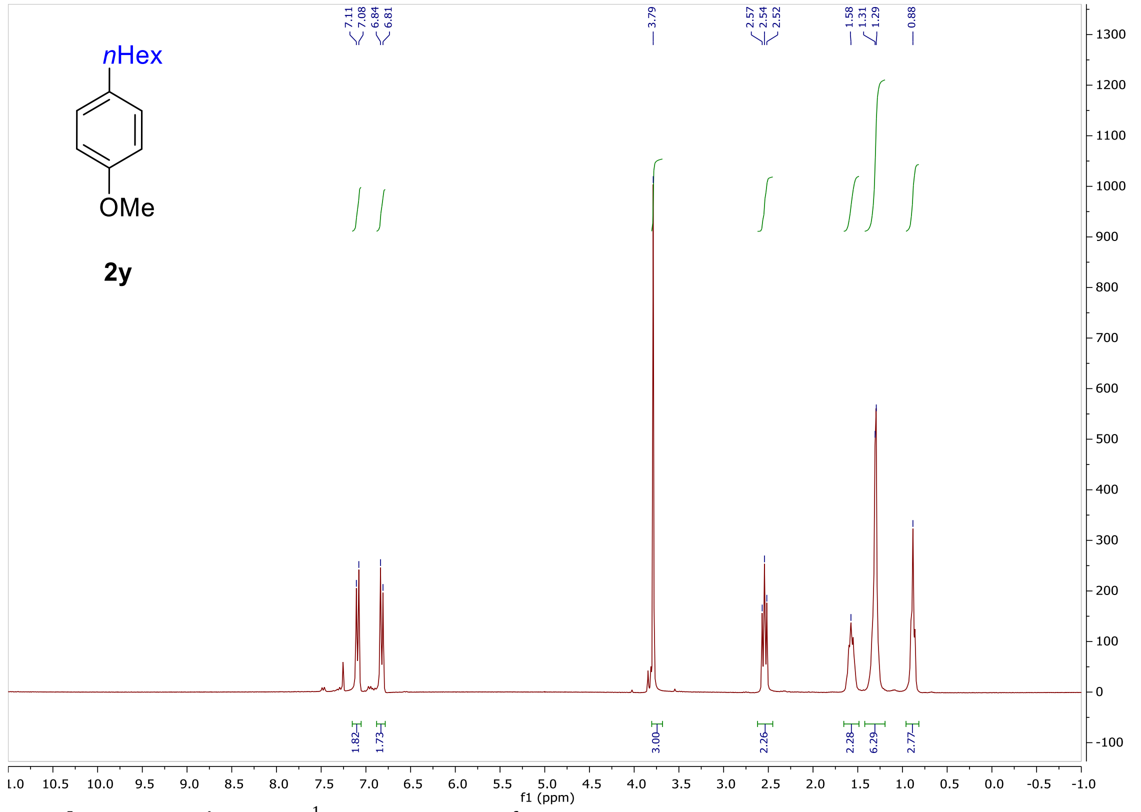

Supplementary Figure 57. ^{1}H NMR spectra of **2y**

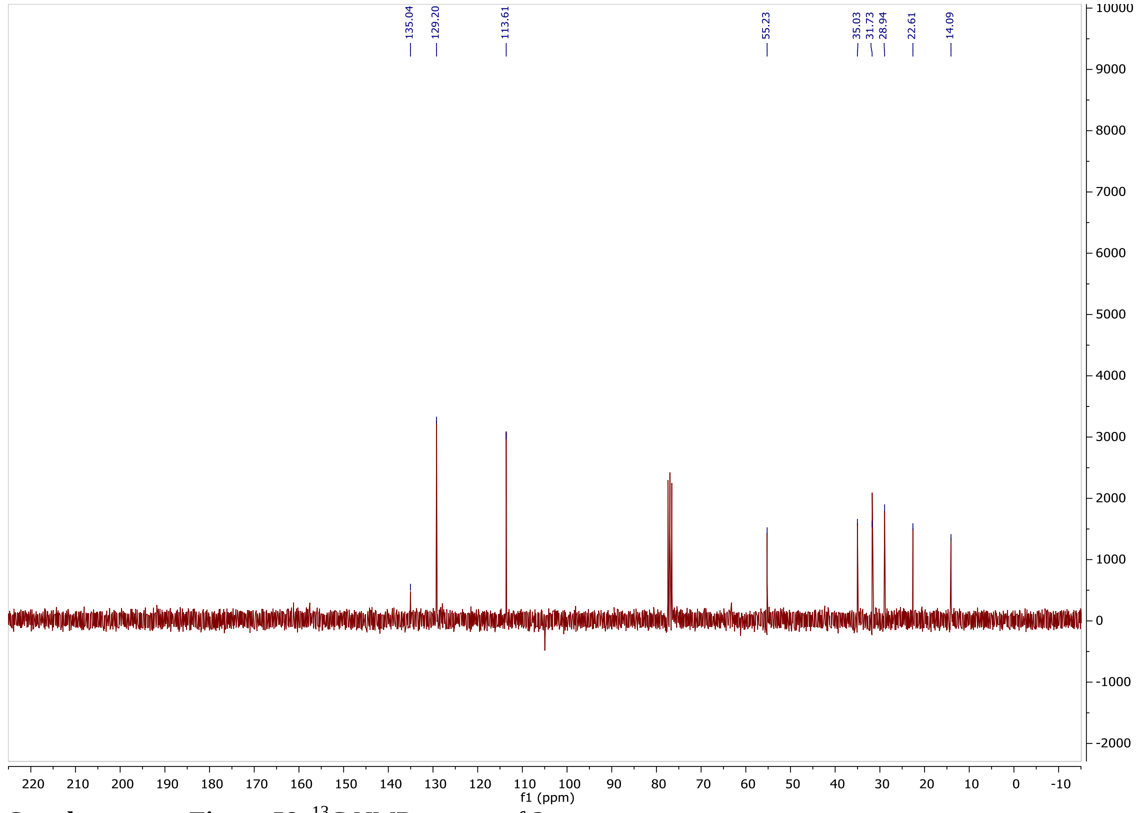

Supplementary Figure 58. ^{13}C NMR spectra of **2y**

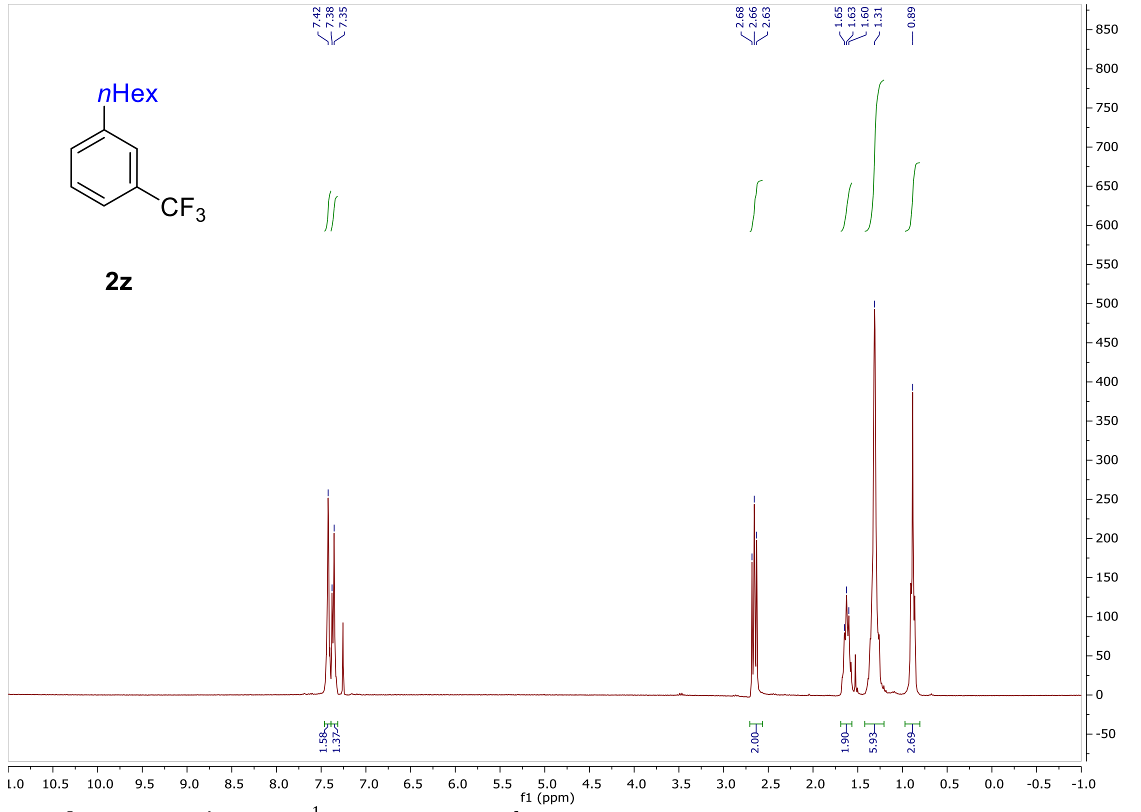

Supplementary Figure 59. ^{1}H NMR spectra of **2z**

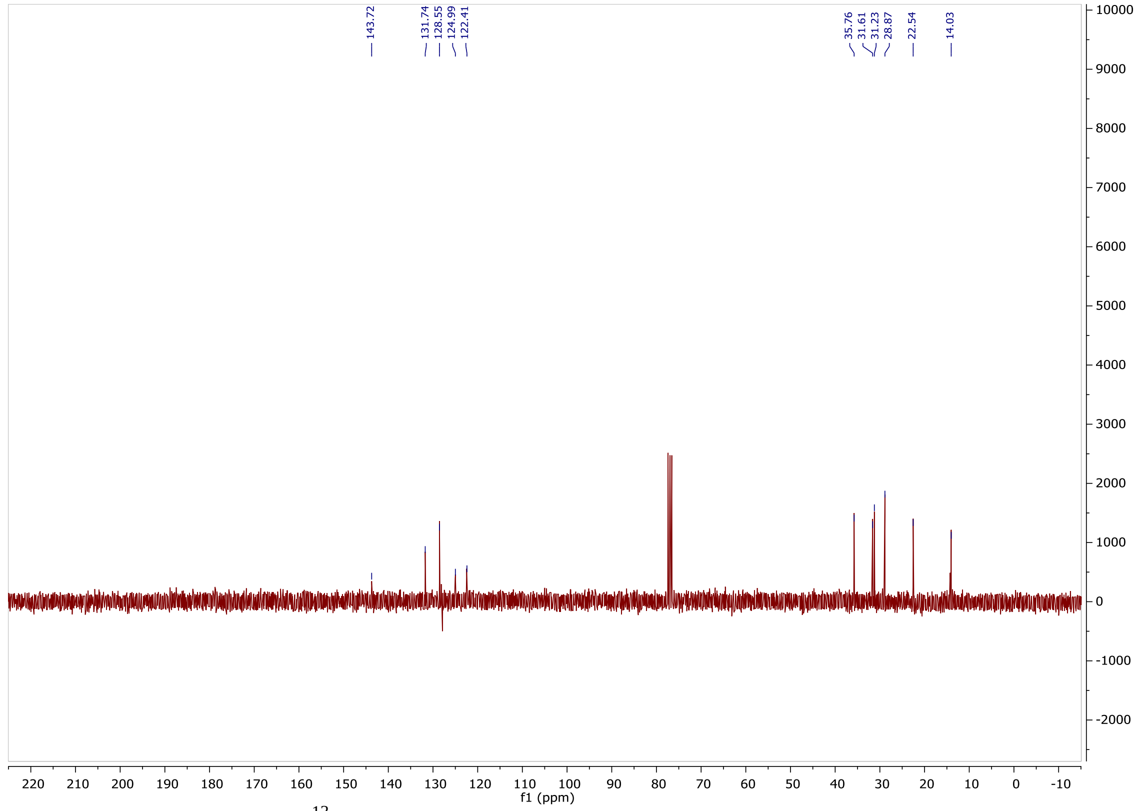

Supplementary Figure 60. ^{13}C NMR spectra of **2z**

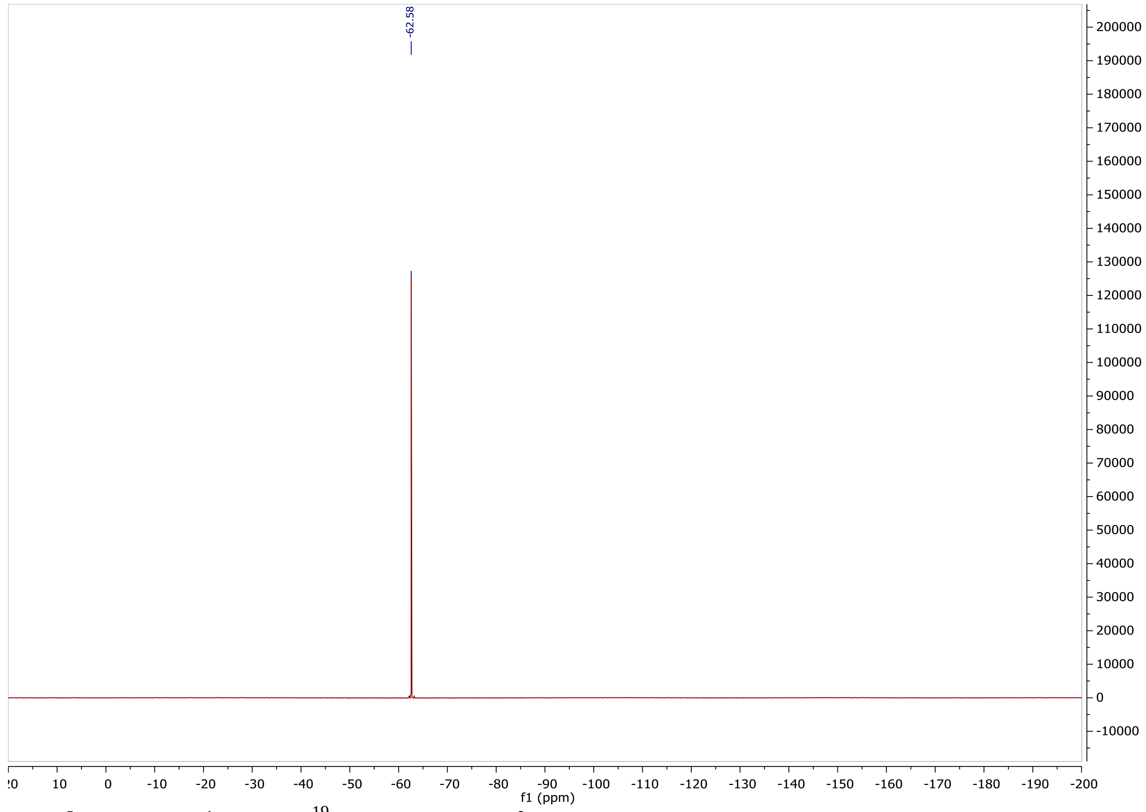

Supplementary Figure 61. ^{19}F NMR spectra of **2z**

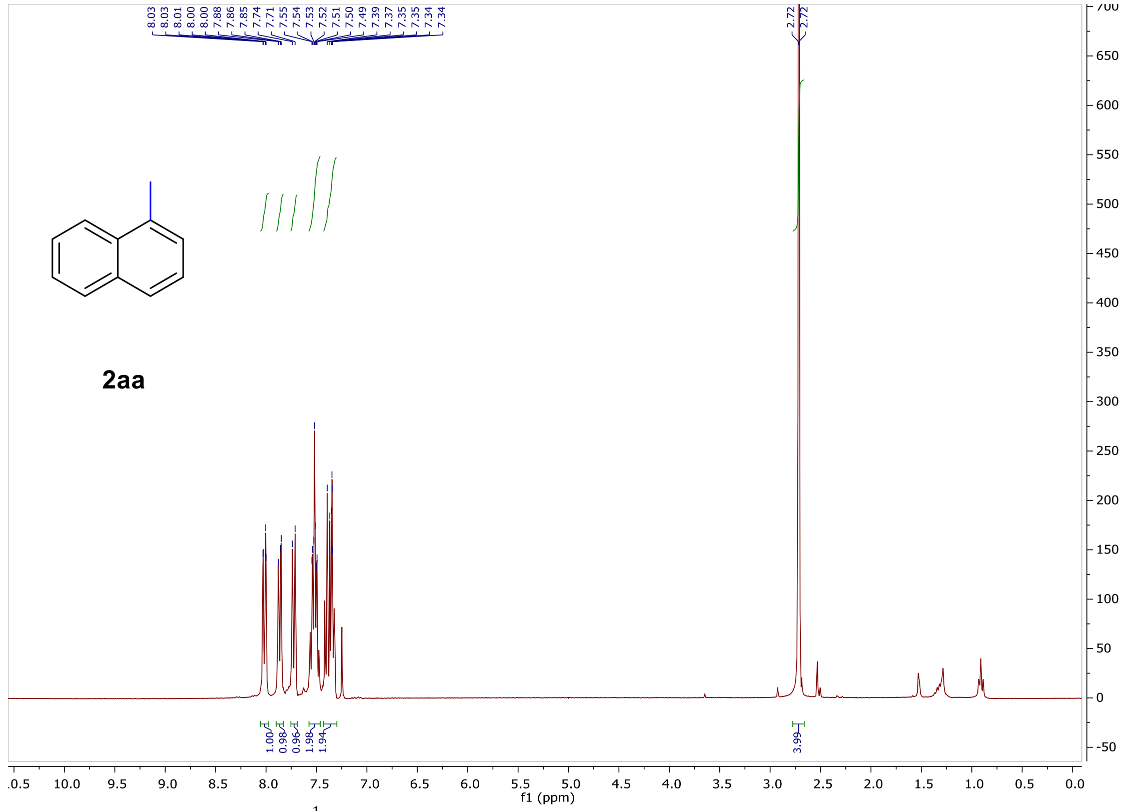

Supplementary Figure 62. ^{1}H NMR spectra of **2aa**

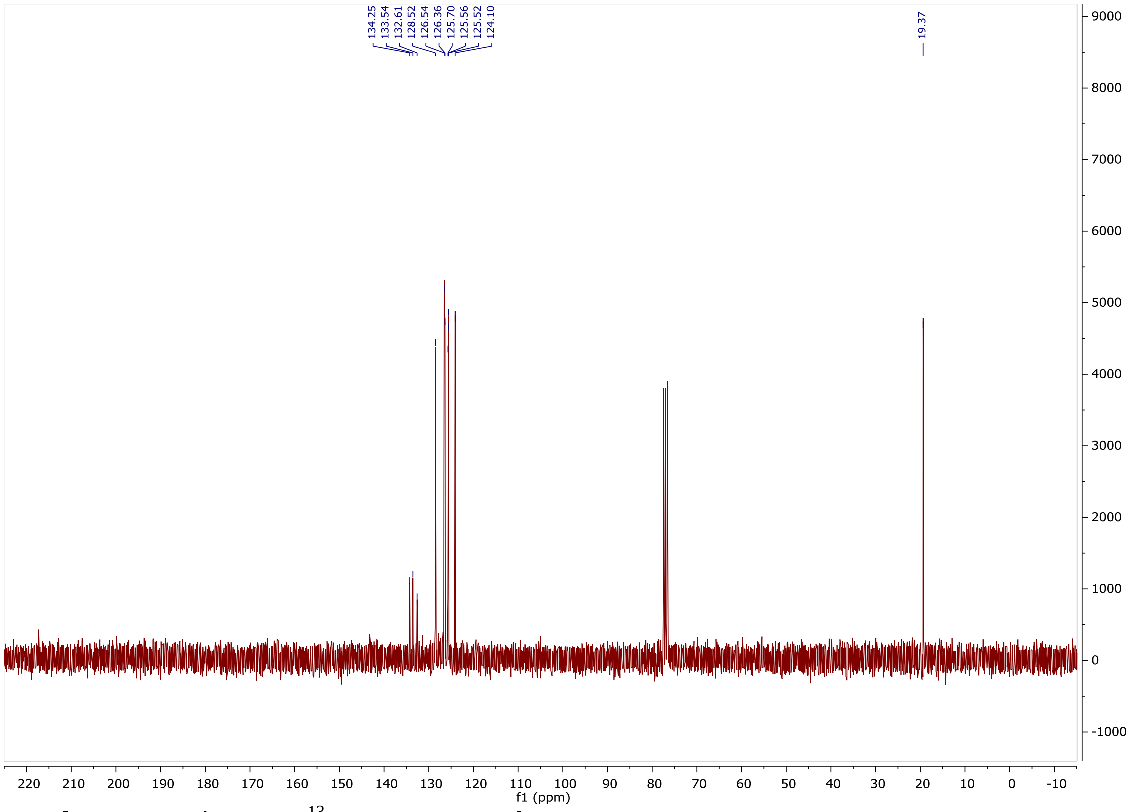

Supplementary Figure 63. ^{13}C NMR spectra of **2aa**

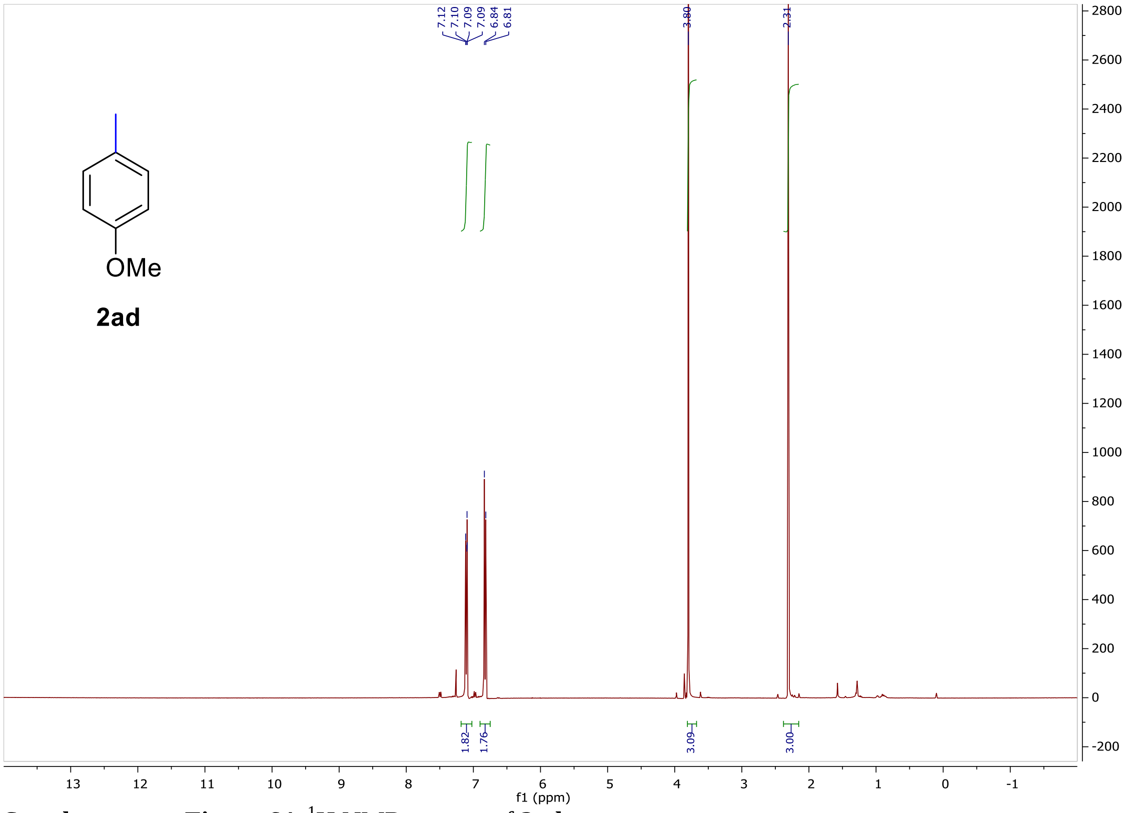

Supplementary Figure 64. ^{1}H NMR spectra of **2ad**

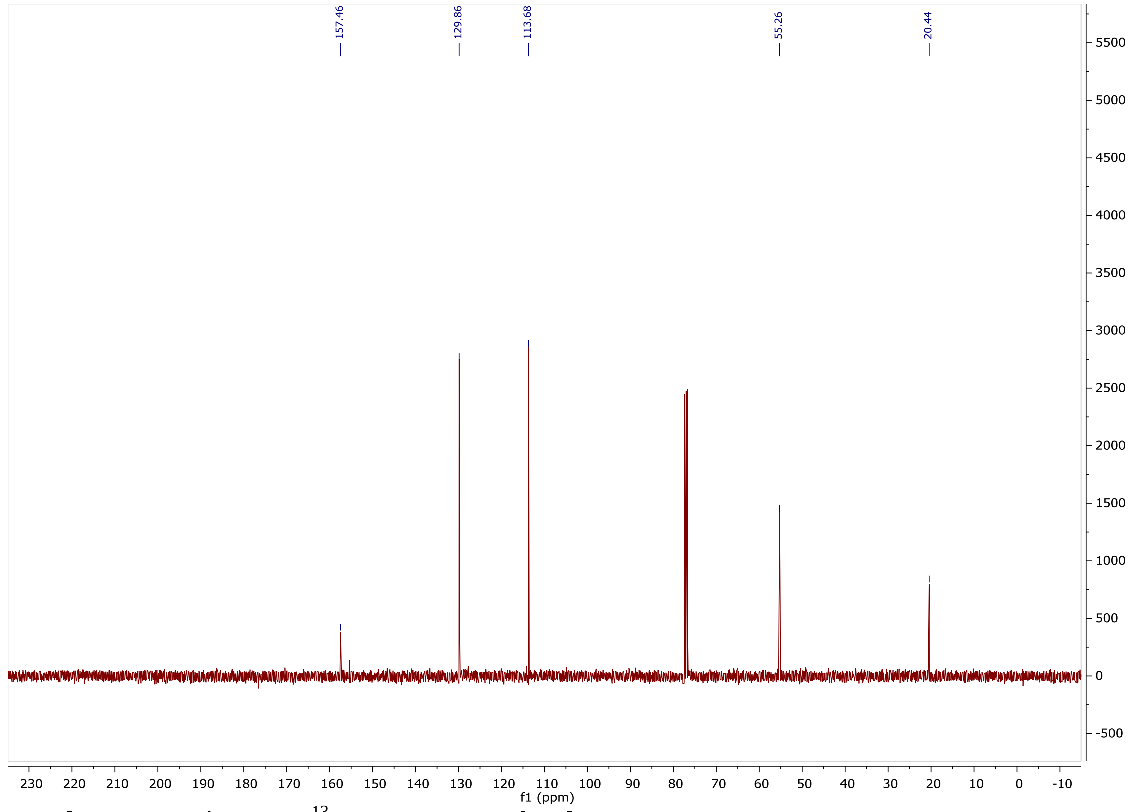

Supplementary Figure 65. ^{13}C NMR spectra of **2ad**

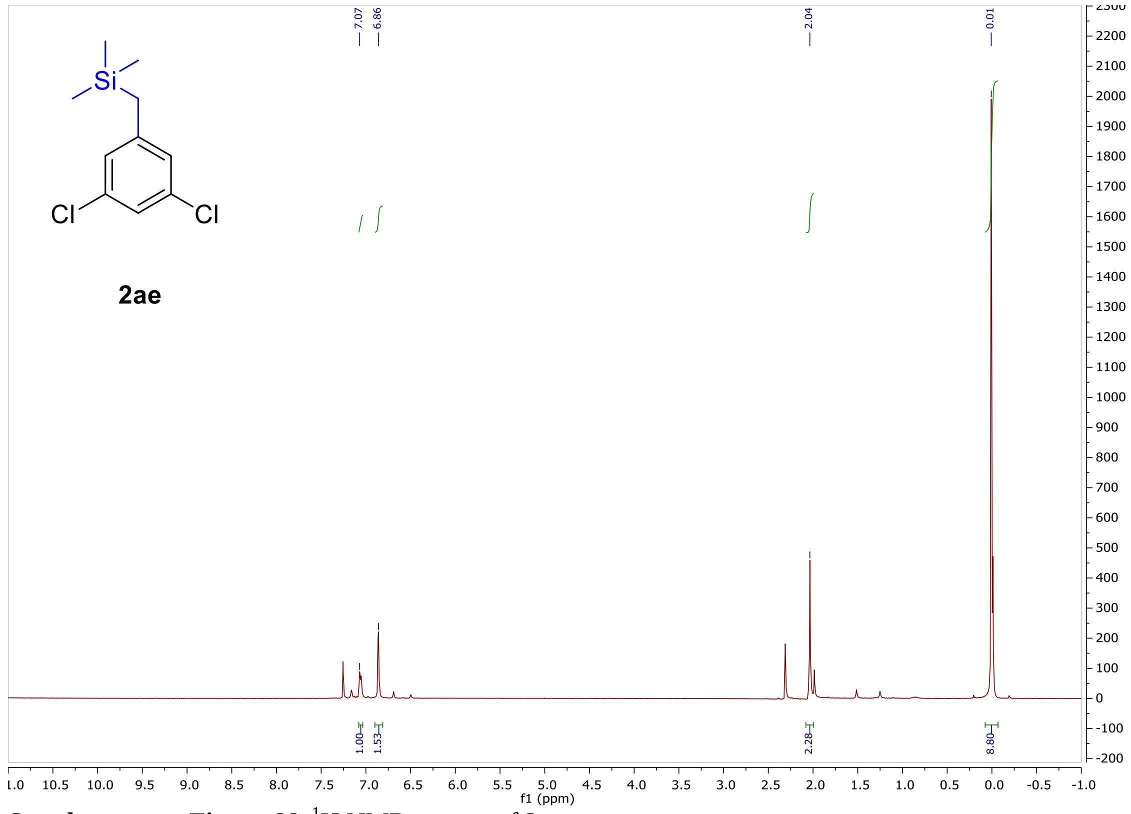

Supplementary Figure 66. ^{1}H NMR spectra of **2ae**

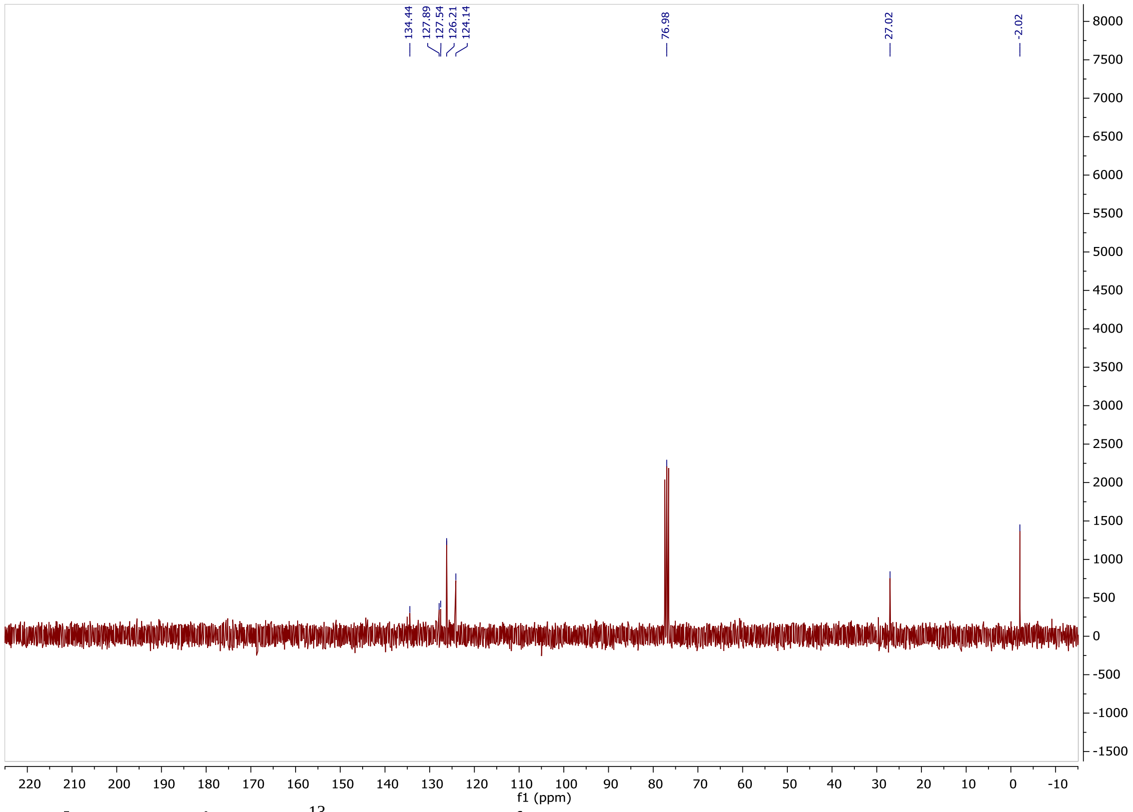

Supplementary Figure 67. ^{13}C NMR spectra of **2ae**

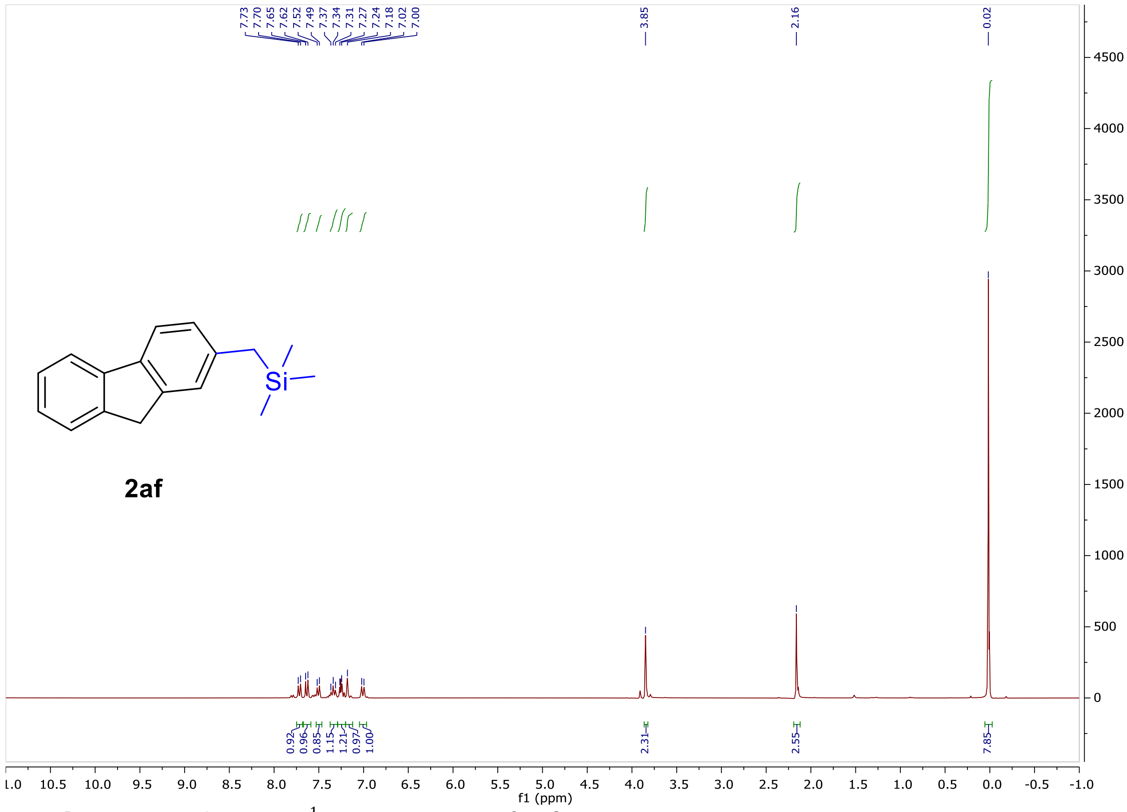

Supplementary Figure 68. ^{1}H NMR spectra of **2af**

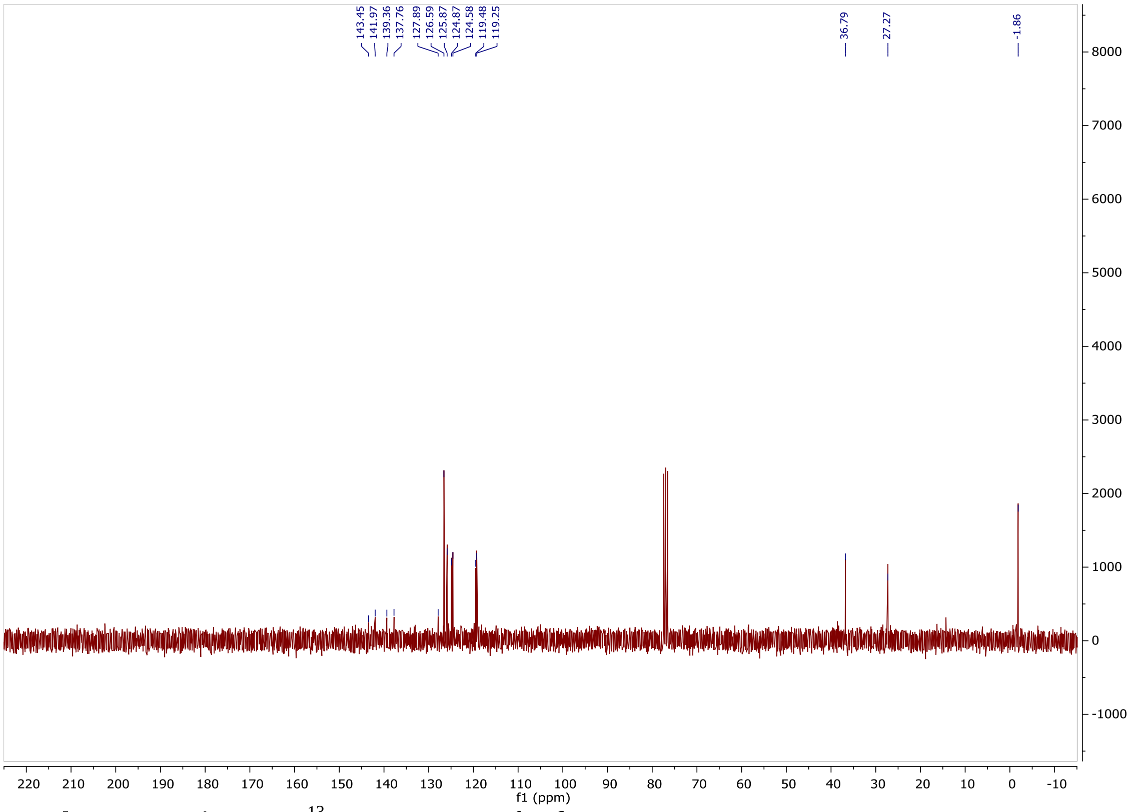

Supplementary Figure 69. ^{13}C NMR spectra of **2af**

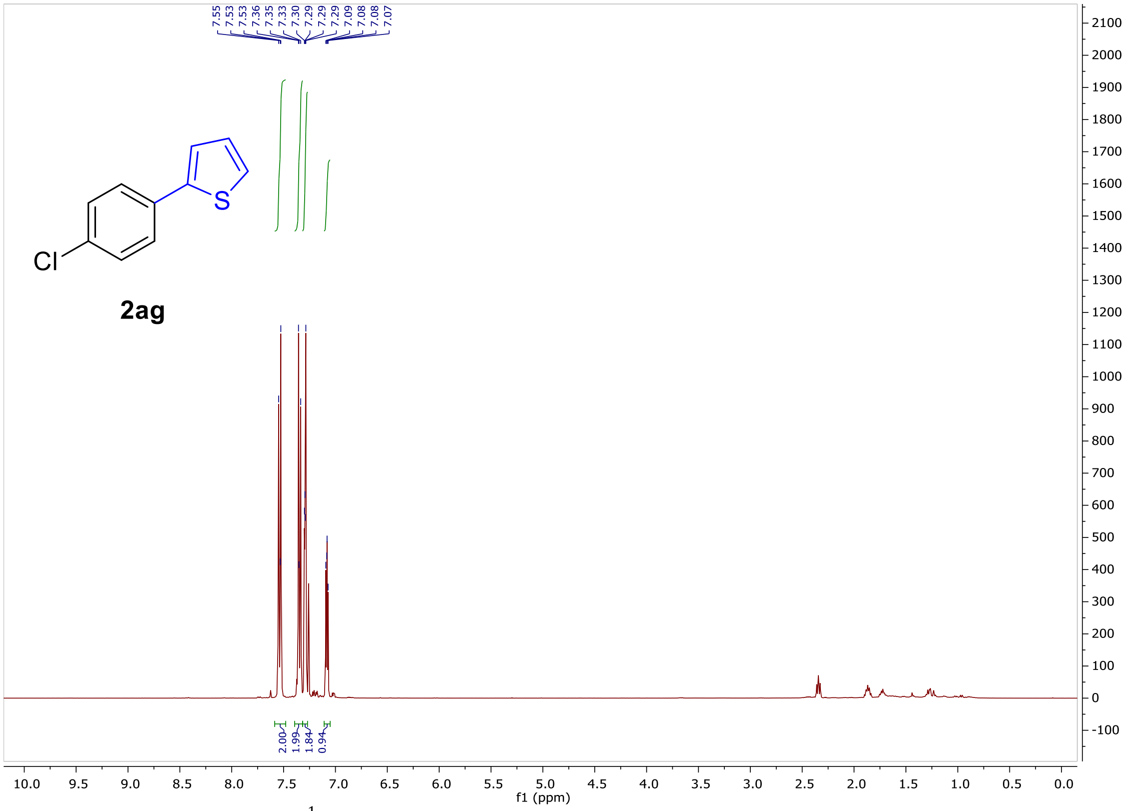

Supplementary Figure 70. ^{1}H NMR spectra of **2ag**

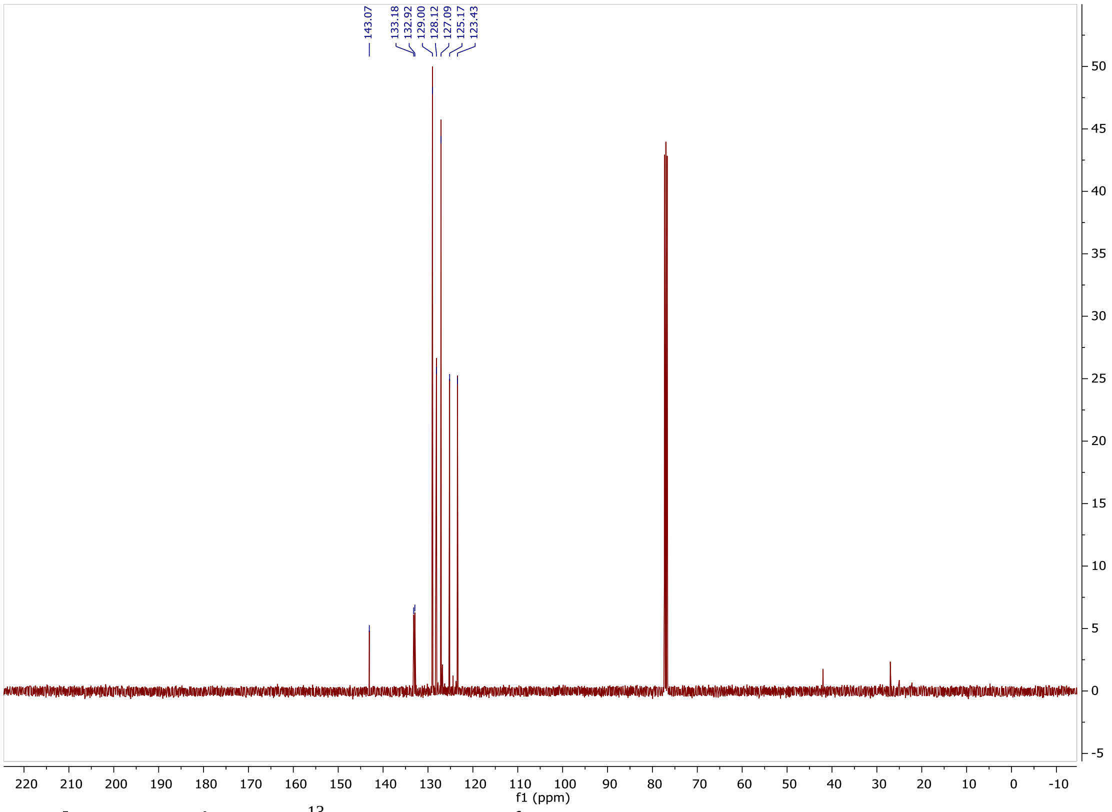

Supplementary Figure 71. ^{13}C NMR spectra of **2ag**

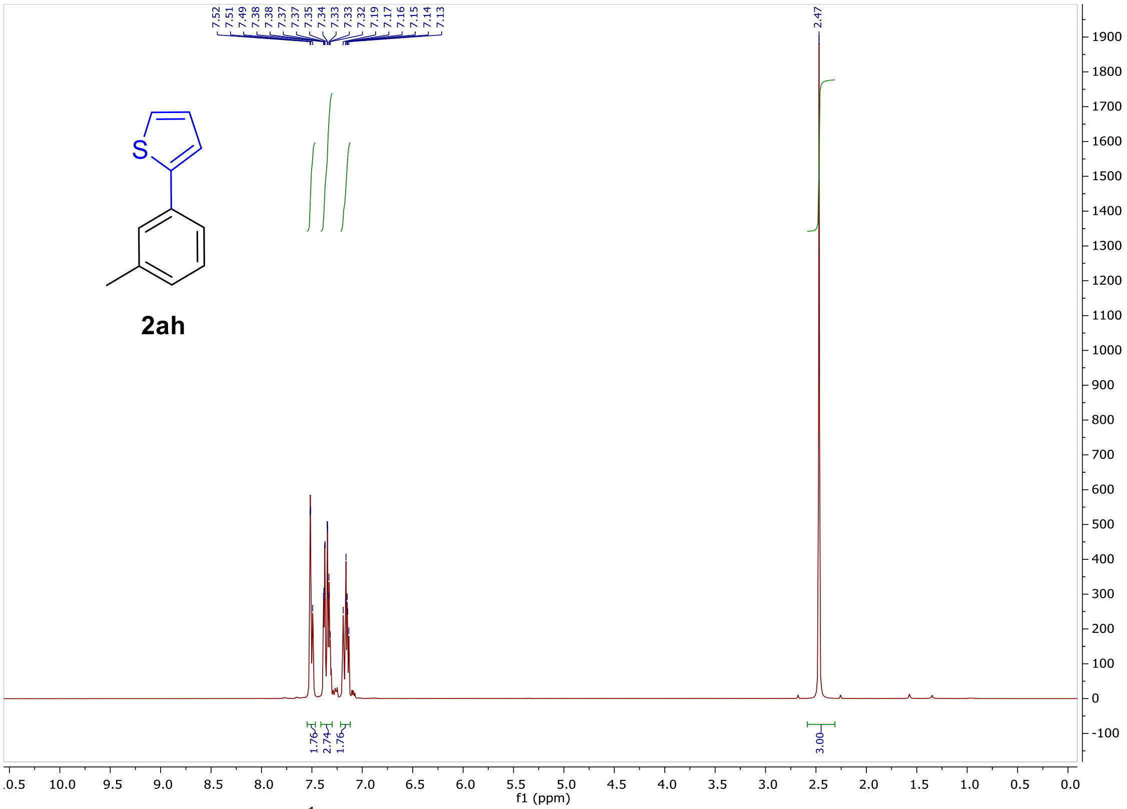

Supplementary Figure 72. ^{1}H NMR spectra of **2ah**

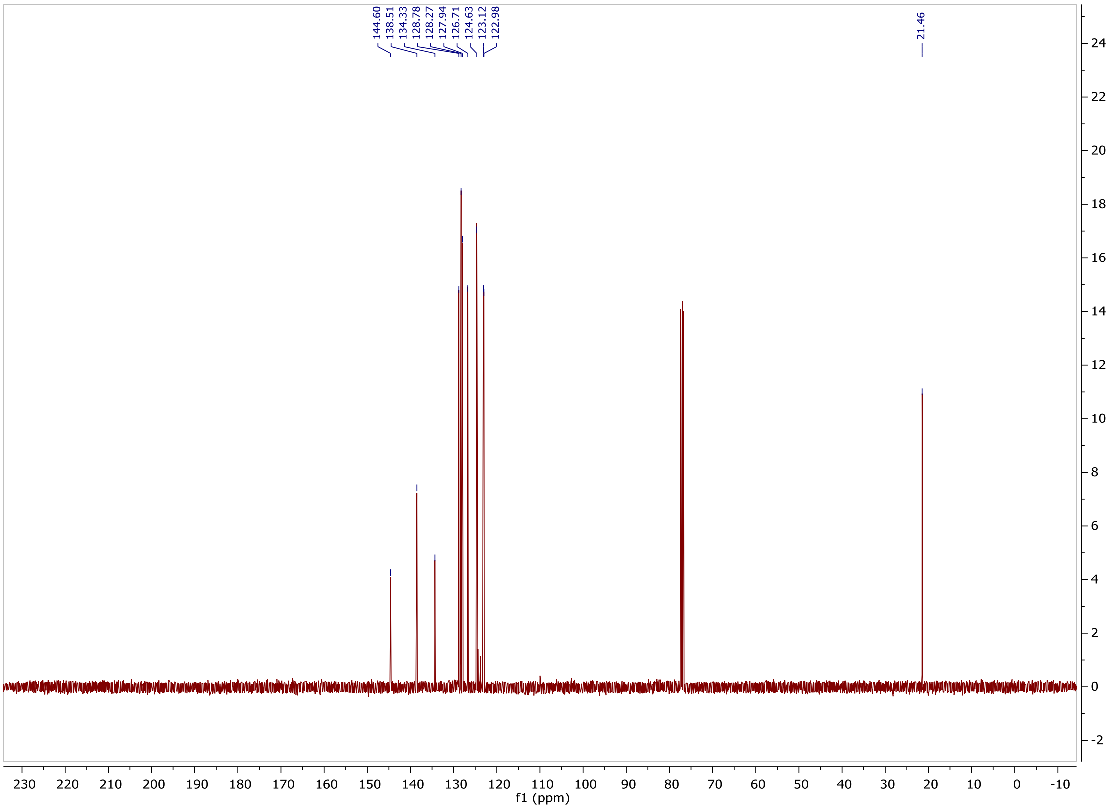

Supplementary Figure 73. ^{13}C NMR spectra of **2ah**

Supplementary Tables

Supplementary Table 1. Pd-catalysed cross-coupling reaction of organolithium compounds and organic halides employing deep eutectic solvents (DES). Conditions: Commercial available PhLi (1.8 M in $n\text{Bu}_2\text{O}$) was added to a mixture of **1b** (0.3 mmol, 56 mg) and Pd-PEPPSI-*i*Pr (10 mol %) in 1 mL of DES. Conversion determined by GC analysis.

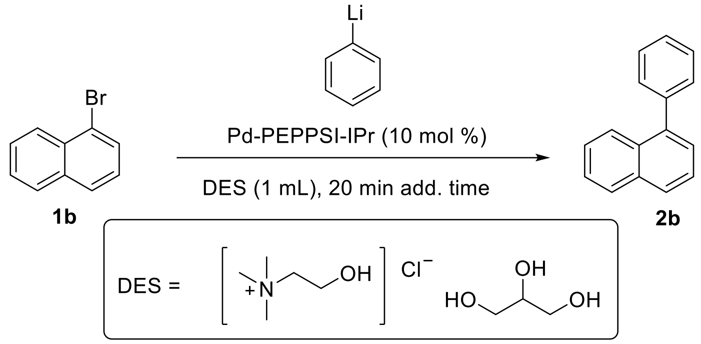

entry	PhLi (equiv.)	Magnetic stirring speed	Reaction time after addition	Conversion
1	2 eq	1200 rpm	5 min	30%
2	10 eq	1200 rpm	5 min	53%
3	2 eq	300 rpm	2 hours	28%

Supplementary Notes:

Supplementary Note 1: The transformations described here have been performed under N_2 atmosphere. However, we have repeated the synthesis (e.g. compound **2m**) keeping the Schlenk flask open to the air and a similar selectivity (>99%) and isolated yield (95%) was obtained.

Supplementary Note 2: The authors have not experienced significant problems of exothermicity in comparison to usual couplings (or other catalytic reactions). The synthesis of **2aa** was performed on 6 mmol scale (1.25 g), with a small increase of temperature of 4 °C (from 25 °C to 28 °C) upon addition of the organolithium reagents.

Supplementary Note 3: The authors have performed a cross coupling of 1-bromonaphthalene and dry MeLi, by removing the solvent under vacuum of a commercial organolithium reagent, and subsequent transferring to a glove box. The cross coupling works although, with strongly reduced selectivity in which **2aa** was formed up to 15%. We do explicitly warn for the pyrophoric nature of dry organolithium species.

Supplementary Note 4: The authors have performed a cross coupling of 1-bromonaphthalene and phenyl lithium under conditions given in general procedure A, however using 1 eq of the lithium species, rather than 1,2. Compound **2b** was obtained in similar conversion and yield.

Supplementary Note 5: We did not experience any problem with salt formation (for instance on stirring the reaction mixture) under any of the conditions we used.

Supplementary Note 6: Experimental procedure and calculation of the E-factor for the synthesis of **2ag**, including aqueous work-up: The reaction mixture was quenched with 1 mL of water, extracted with 1 mL of AcOEt and the organic phase was dried with anhydrous Na_2SO_4. Evaporation of the solvent under reduced pressure afforded the crude product that was then filtered over a silica gel plug to afford the pure product. Yield 97%. The E factor including the water used for the work-up is 15.4. The E factor reported in literature for the Suzuki coupling is 84.

Supplementary Methods:

General

Chromatography: Merck silica gel type 9385 230-400 mesh, TLC: Merck silica gel 60, 0.25 mm. Components were visualized by UV and cerium/molybdenum or potassium permanganate staining. Progress and conversion of the reaction were determined by GC-MS (GC, HP6890: MS HP5973) with an HP1 or HP5 column (Agilent Technologies, Palo Alto, CA). Mass spectra were recorded on an AEI-MS-902 mass spectrometer (EI+) or a LTQ Orbitrap XL (ESI+). ^{1}H- and ^{13}C-NMR were recorded on a Varian AMX400 (400 and 100.59 MHz, respectively) using $CDCl_3$ as solvent. Chemical shift values are reported in ppm with the solvent resonance as the internal standard ($CHCl_3$: δ 7.26 for ^{1}H, δ 77.0 for ^{13}C). Data are reported as follows: chemical shifts, multiplicity (s = singlet, d = doublet, t = triplet, q = quartet, br = broad, m = multiplet), coupling constants (Hz), and integration. All reactions were carried out under a nitrogen atmosphere. THF and Et_2O were dried and distilled over sodium. Pd[P(*t*-Bu)$_3$]$_2$, was purchased from Strem, $Pd_2(dba)_3$, XPhos, Pd-PEPPSI-*i*Pr and Pd-PEPPSI-*i*Pent were purchased from Aldrich and used without further purification. *n*-BuLi (1.6 M solution in hexane) was purchased from Acros. PhLi (1.8 M solution in dibutylether), MeLi (1.6 M in diethylether), $TMSCH_2Li$ (1.0 M in pentane), *n*-HexLi (2.3 M in hexane), 2-thienylLi (1.0 M in THF/hexane), and the compounds used as precursor for the preparation of lithium reagents, namely 1-bromo-2,6-dimethoxy-benzene, 1-bromo-4-methylbenzene and 1-bromo-4-(trifluoromethyl)benzene were purchased from Aldrich. All the bromides were commercially available and were purchased from Aldrich, TCI Europe N.V. and Acros Organics. *p*-tolyllithium, (4-(trifluoromethyl)phenyl)lithium and (2,6-dimethoxyphenyl)lithium were prepared according to described procedures.[1,2,3,4] E factors were calculated according to the procedure reported by Lipshutz et.al..[5]

General Procedure A for the Cross-Coupling with (Hetero)aryllithium Reagents

The corresponding commercially available or homemade (hetero)aryllithium reagent was added over a mixture of substrate (1 mmol) and Pd-PEPPSI-*i*Pr (1.5 mol %, 10.5 mg) at room temperature for 10 min. After the addition was completed a saturated solution of aqueous NH_4Cl was added and the mixture was extracted with AcOEt or Et_2O. The organic phases were combined and dried with anhydrous Na_2SO_4. Evaporation of the solvent under reduced pressure afforded the crude product that was then filtered over a silica gel plug.

General Procedure B for the Cross-Coupling with Alkyllithium Reagents

The corresponding commercially available alkyllithium reagent was added over a mixture of substrate (1 mmol) and Pd[P(*t*-Bu)$_3$]$_2$ (2 mol%, 10 mg) at room temperature for 10 min. After the addition was completed a saturated solution of aqueous NH_4Cl was added and the mixture was extracted with AcOEt or Et_2O. The organic phases were combined and dried with anhydrous Na_2SO_4. Evaporation of the solvent under reduced pressure afforded the crude product that was then filtered over a silica gel plug.

General Procedure C for Reactions Carried out in 120 mmol Scale

Commercially available *n*-BuLi (100 mL, 1.6 M solution in hexane) was added via cannula over a mixture of substrate (120 mmol, 27 g) and Pd[P(*t*-Bu)$_3$]$_2$ (0.4 mol%, 250 mg) at room temperature for 30 min, keeping the temperature between 20-25 °C with the use of an additional water bath. After the addition was completed water was slowly added and the mixture was extracted with AcOEt or Et_2O. The organic phase were combined and dried with anhydrous Na_2SO_4 and solvent was removed under reduced pressure affording the final product in reagent grade quality.

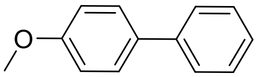

4-Methoxybiphenyl (2a):[3]
CAS Registry Number: 613-37-6.
Synthesized using catalytic system A with 1-bromo-4-methoxybenzene (1 mmol, 187 mg) and 798 µL of PhLi.
Catalytic system A: Reaction carried out at room temperature. White solid obtained after filtration over a silica plug (SiO_2, *n*-pentane/ Et_2O 100:1), 155 mg, 84% yield.

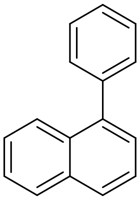

1-Phenylnaphthalene (2b):[6,3]
CAS Registry Number: 605-02-7

Synthesized using catalytic system A with 1-bromonaphthalene (1 mmol, 207 mg) and 798 µL of PhLi.
Catalytic system A: Reaction carried out at room temperature. White solid obtained after filtration over a silica plug (SiO_2, *n*-pentane/Et_2O 100:1), 178 mg, 87% yield.

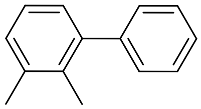

2,3-Dimethyl-1,1'-biphenyl (2c):[7]
CAS Registry Number: 3864-18-4
Synthesized using catalytic system A 1-bromo-2,3-dimethylbenzene (1 mmol, 185 mg) and 798 µL of PhLi.
Catalytic system A: Reaction carried out at room temperature. White solid obtained after filtration over a silica plug (SiO_2, *n*-pentane/Et_2O 100:1), 159 mg, 87% yield.

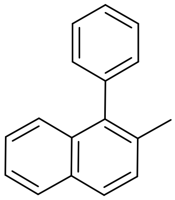

2-Methyl-1-phenylnaphthalene (2d):[8]
CAS Registry Number: 29304-63-0
Synthesized using catalytic system A with 1-bromo-2-methylnaphthalene (1 mmol, 221 mg) and 798 µL of PhLi. White solid obtained after filtration over a silica plug (SiO_2, *n*-pentane/ Et_2O 100:1), 196 mg, 90% yield.

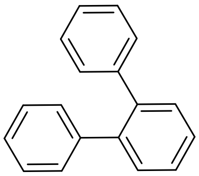

1,1':2',1"-Terphenyl (2e):[9]
CAS Registry Number: 84-15-1
Synthesized using catalytic system A with 2-bromo-1:1'-biphenyl (1 mmol, 233 mg) and 798 µL of PhLi. Colorless oil obtained after filtration over a silica plug (SiO_2, *n*-pentane/ Et_2O 100:1), 225 mg, 98% yield.

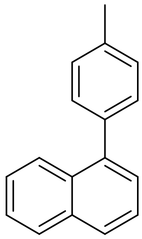

1-(*p*-Tolyl)naphthalene (2f):[6]
CAS Registry Number: 27331-34-6
Synthesized using catalytic system A with 1-bromonaphthalene (1 mmol, 207 mg) and 2394 µL of *p*-tolyllithium (0.6 M solution in diethylether).
White solid obtained after filtration over a silica plug (SiO_2, *n*-pentane/ Et_2O 100:1), 198 mg, 91% yield.

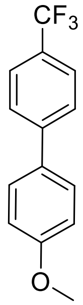

4-Methoxy-4'-(trifluoromethyl)-1,1'-biphenylphenyl (2g):[8]
CAS Registry Number: 10355-12-1
Synthesized using catalytic system A with 1-bromo-4-methoxybenzene (1 mmol, 187 mg) and 2394 µL of *p*-trifluoromethylphenyllithium (0.6 M solution in diethylether). Off white solid obtained after filtration over a silica plug (SiO_2, *n*-pentane/ Et_2O 100:1), 229 mg, 91% yield.

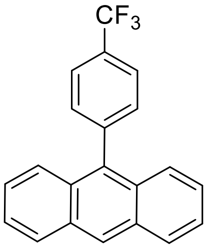

9-(4-(Trifluoromethyl)phenyl)anthracene (2h):[10]
CAS Registry Number: 386-23-2
Synthesized using catalytic system A with 9-bromoanthracene (1 mmol, 257 mg) and 2394 µL of *p*-trifluoromethylphenyllithium (0.6 M solution in diethylether). Yellow solid obtained after filtration over a silica plug (SiO_2, *n*-pentane/ Et_2O 100:1), 305 mg, 92% yield.

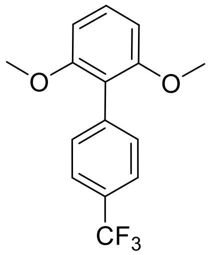

2,6-Dimethoxy-4'-(trifluoromethyl)-1,1'-biphenylphenyl (2i):[11]
CAS Registry Number: 603112-21-6
Synthesized using catalytic system A with 1-bromo-4-(trifluoromethyl)benzene (1 mmol, 225 mg) and 2394 µL of 2,3-dimethoxy-phenyllithium (0.6 M solution in diethylether). White solid obtained after filtration over a silica plug (SiO_2, *n*-pentane/ Et_2O 100:1), 274 mg, 97% yield.

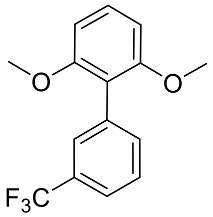

2,6-Dimethoxy-3'-(trifluoromethyl)-1,1'-biphenylphenyl (2j):[12]
CAS Registry Number: 603112-20-5
Synthesized using catalytic system A with 1-bromo-3-(trifluoromethyl)benzene (1 mmol, 225 mg) and 2394 µL of 2,3-dimethoxy-phenyllithium (0.6 M solution in diethylether). White solid obtained after filtration over a silica plug (SiO_2, *n*-pentane/ Et_2O 100:1), 237 mg, 84% yield.

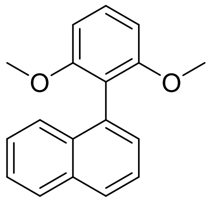

1-(2,6-Dimethoxyphenyl)naphthalene (2k):[13]
CAS Registry Number: 173300-93-1
Synthesized using catalytic system A with 1-bromonaphthalene (1 mmol, 207 mg) and 2394 µL of of 2,3-dimethoxy-phenyllithium (0.6 M solution in diethylether). White solid obtained after filtration over a silica plug (SiO_2, *n*-pentane), 222 mg, 84% yield.

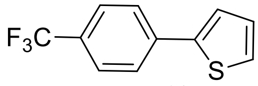

2-(4-(Trifluoromethyl)phenyl)thiophene (2l):[14]
CAS Registry Number: 115933-15-8.

Synthesized using catalytic systems B with 1-bromo-4-(trifluoromethyl)benzene (1 mmol, 225 mg) and 1200 µL of 2-thienyllithium. White solid obtained after column chromatography (SiO_2, *n*-pentane/ EtOAc 100:1), 219 mg, 96% yield.

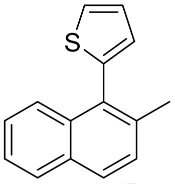

1-(2-Methylnaphthalen-1-yl)thiophene (2m):[15]
CAS Registry Number: 1064187-66-1
Synthesized using catalytic system B with 2-methyl-1-bromonaphthalene (1 mmol, 221 mg) and 1200 µL of 2-thienyllithium. Colorless oil obtained after filtration over a silica plug (SiO_2, *n*-pentane), 220 mg, 98% yield.

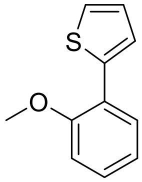

2-(2-Methoxyphenyl)thiophene (2n):[16]
CAS Registry Number: 17595-92-5
Synthesized using catalytic system B with 1-bromo-2-methoxybenzene (1 mmol, 187 mg) and 1200 µL of 2-thienyllithium. White solid obtained after filtration over a silica plug (SiO_2, *n*-pentane), 165 mg, 87% yield.

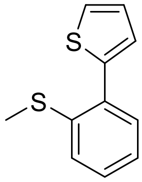

2-(2-(Methylthio)phenyl)thiophene (2o):
Synthesized using catalytic system B with (2-bromophenyl)(methyl)sulfane (1 mmol, 202 mg) and 1200 µL of 2-thienyllithium.Yellow oil obtained after column chromatography (SiO_2, *n*-pentane/EtOAc 100:1), 88% yield.^{1}H NMR (300 MHz, $CDCl_3$) δ 7.44 (q, J = 5.9, 5.1 Hz, 2H), 7.41 – 7.29 (m, 3H), 7.24 (t, J = 7.1 Hz, 1H), 7.20 – 7.14 (m, 1H), 2.48 (s, 3H). ^{13}C NMR (75 MHz, $CDCl_3$) δ 141.29, 138.07 , 133.24 , 130.95 , 128.43 , 127.58 , 127.09 , 125.90 , 125.61 , 124.76 , 16.17. HRMS (APCI+, *m/z*): calculated for $C_{11}H_{11}S_2$ [M+H$^+$]: 207.03022; found: 207.03149.

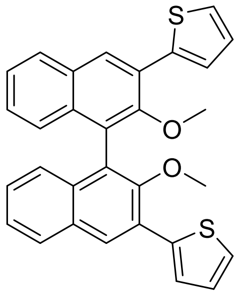

2,2'-(2,2'-Dimethoxy-[1,1'-binaphthalene]-3,3'-diyl)dithiophene (2p):
Synthesized using catalytic system B with 3,3'-dibromo-2,2'-dimethoxy-1,1'-binaphthalene (1 mmol, 472 mg) and 2400 µL of 2-thienyllithium. Yellow solid obtained after column chromatography (SiO_2, *n*-pentane/EtOAc 100:1), 81% yield. ^{1}H NMR (400 MHz, $CDCl_3$) δ 8.24 (s, 2H), 7.91 (d, J = 8.2 Hz, 2H), 7.67 (d, J = 3.5 Hz, 2H), 7.40 (q, J = 4.9, 4.3 Hz, 4H), 7.24 (t, J = 7.1 Hz, 3H), 7.16 (dd, J = 9.7, 6.1 Hz, 4H), 3.34 (s, 6H). ^{13}C NMR (100 MHz, $CDCl_3$) δ 153.32, 139.55, 133.48, 130.71, 128.72, 128.06, 127.96, 127.23, 126.48, 126.31, 126.26, 125.86, 125.72, 125.29, 60.49. HRMS (APCI+, *m/z*): calculated for $C_{30}H_{23}O_2S_2$ [M+H$^+$]: 479.11395; found: 479.11182.

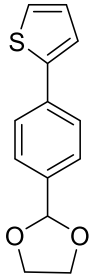

2-(4-(Thiophen-2-yl)phenyl)-1,3-dioxolane (2q):[16]
CAS Registry Number: 81707-47-3
Synthesized using catalytic system B with 2-bromo-4-phenyl)-1,3-dioxolane (1 mmol, 229 mg) and 1200 µL of 2-thienyllithium. White solid obtained after filtration over a silica plug (SiO_2, *n*-pentane), 197 mg, 85% yield.

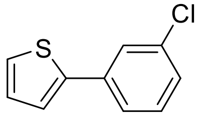

2-(3-Chlorophenyl)thiophene (2r):[17]
CAS Registry Number: 59156-10-4
Synthesized using catalytic system B with 1-bromo-3-chlorobenzene (1 mmol, 191 mg) and 1200 µL of 2-thienyllithium. White solid obtained after filtration over a silica plug (SiO_2, *n*-pentane), 169 mg, 87% yield.

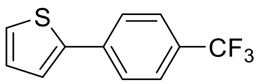

2-(4-(Trifluoromethyl)phenyl)thiophene (2l):[3]
CAS Registry Number: 115933-15-8
Synthesized using catalytic system B with 1-chloro-4-(trifluoromethyl)benzene (1 mmol, 180 mg) and 1200 µL of 2-thienyllithium. White solid obtained after filtration over a silica plug (SiO_2, *n*-pentane), 198 mg, 87% yield.

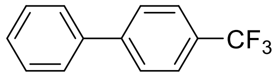

4-(Trifluoromethyl)-1,1'-biphenyl (2s):[18]
CAS Registry Number: 398-36-7
Synthesized using catalytic system A with 1-chloro-4-(trifluoromethyl)benzene (1 mmol, 180 mg) and 798 µL of PhLi. White solid obtained after filtration over a silica plug (SiO_2, *n*-pentane), 187 mg, 84% yield.

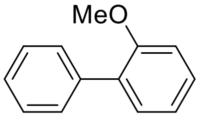

2-(Methoxy)-1,1'-biphenyl (2t):[19]
CAS Registry Number: 86-26-0
Synthesized using catalytic system A with 1-chloro-2-methoxybenzene (1 mmol, 142 mg) and 798 µL of PhLi. White solid obtained after filtration over a silica plug (SiO_2, *n*-pentane), 155 mg, 86% yield.

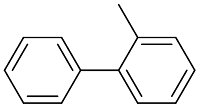

2-(Methyl)-1,1'-biphenyl (2u):[20]
CAS Registry Number: 643-58-3
Synthesized using catalytic system A with 2-chloro-toluene (1 mmol, 126 mg) and 798 µL of PhLi. White solid obtained after filtration over a silica plug (SiO_2, *n*-pentane), 141 mg, 84% yield.

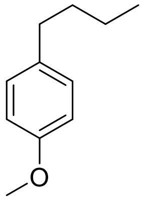

1-Butyl-4-methoxybenzene (2v).[13]
CAS Registry Number: 18272-84-9

Synthesized using catalytic systems C with 1-bromo-4-methoxybenzene (1 mmol, 187 mg) and 750 µL of *n*-butyllithium. Colorless oil obtained after filtration over a silica plug (SiO_2, *n*-pentane), 135 mg, 82% yield.

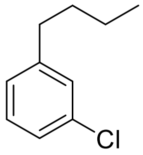

1-Butyl-3-chlorobenzene (2w).[21]
CAS Registry Number: 15499-28-2
Synthesized using catalytic systems C with 1-bromo-3-chlorobenzene (1 mmol, 191 mg) and 750 µL of *n*-butyllithium. Colorless oil obtained after filtration over a silica plug (SiO_2, *n*-pentane), 144 mg, 85% yield.

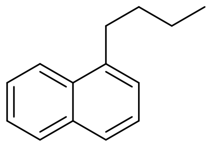

1-Butylnaphthalene (2x).[22]
CAS Registry Number: 1634-09-9.
Synthesized using catalytic systems C with 1-bromo-naphthalene (1 mmol, 207 mg) and 750 µL of *n*-butyllithium. Colorless oil obtained after filtration over a silica plug (SiO_2, *n*-pentane), 173 mg, 94% yield.

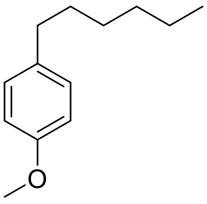

1-Hexyl-4-methoxybenzene (2y).[22]
CAS Registry Number: 81693-80-3.
Synthesized using catalytic systems C with 1-bromo-4-methoxybenzene (1 mmol, 187 mg) and 520 µL of *n*-hexyllithium. Colorless oil obtained after filtration over a silica plug (SiO_2, *n*-pentane), 157 mg, 82% yield.

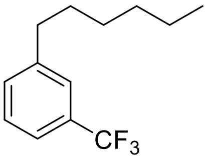

1-Hexyl-3-(trifluoromethyl)benzene (2z).

Synthesized using catalytic systems C with 1-bromo-3-(trifluoromethyl)benzene (1 mmol, 225 mg) and 520 µL of *n*-hexyllithium. Colorless oil obtained after column chromatography (SiO_2, *n*-pentane/EtOAc 100:1), 84% yield. ^1H NMR (400 MHz, $CDCl_3$) δ 7.46-7.32 (m, 4H), 2,66 (t, J = 7.8 Hz, 2H), 1.63 (m, 2H), 1.34 (m, 6H), (t, J = 6.6 Hz, 3H). ^{13}C NMR (100 MHz, $CDCl_3$) δ 187.44, 143.72, 131.74, 128.55, 124.99, 122.40, 35.76, 31.61, 31.23, 28.87, 22.54, 14.03. ^{19}F NMR (376 MHz, $CDCl_3$) δ -62.5. HRMS (APCI+, *m/z*): calculated for $C_{13}H_{18}F_3$ $[M+H^+]$: 231.13606; found: 231.13713.

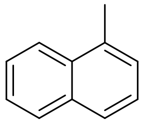

1-Methyl-naphthalene (2aa).[23]
CAS Registry Number: 90-12-0.
Synthesized using catalytic systems C with 1-bromonaphthalene (1 mmol, 207 mg) and 750 µL of methyllithium. Colorless oil obtained after filtration over a silica plug (SiO_2, *n*-pentane), 122 mg, 86% yield.

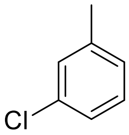

1-Chloro-3-methylbenzene (2ab).[24]
CAS Registry Number: 108-41-8.
Synthesized using catalytic systems C with 1-bromo-3-chlorobenzene (1 mmol, 191 mg) and 750 µL of methyllithium. Colorless oil obtained after filtration over a silica plug (SiO_2, *n*-pentane), 106 mg, 84% yield.

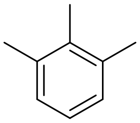

1,2,3-trimethylbenzene (2ac).
CAS Registry Number: 526-73-8.
Synthesized using catalytic systems C with 1,3-dimethyl-2-bromobenzene (1 mmol, 185 mg) and 750 µL of methyllithium. Selectivity based upon GC-results.

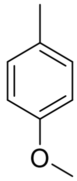

1-Methoxy-4-methylbenzene (2ad).[25]
CAS Registry Number: 104-93-8.

Synthesized using catalytic systems C with 1-bromo-4-methoxybenzene (1 mmol, 187 mg) and 750 µL of methyllithium. Colorless oil obtained after filtration over a silica plug (SiO_2, *n*-pentane), 101 mg, 83% yield.

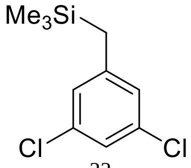

(3,5-Dichlorobenzyl)trimethylsilane (2ae).[22]
CAS Registry Number: 69380-94-5.
Synthesized using catalytic systems C with 1-bromo-3,5-dichlorobenzene (1 mmol, 225 mg) and 1200 µL of (trimethylsilyl)methyllithium. White solid obtained after filtration over a silica plug (SiO_2, *n*-pentane), 196 mg, 84% yield.

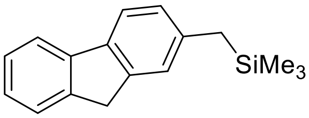

((9*H*-Fluoren-2-yl)methyl)trimethylsilane (2af).[26]
CAS Registry Number: 1694669-89-0
Synthesized using catalytic systems C with 2-bromo-9*H*-fluorene (1 mmol, 245 mg) and 1200 µL of (trimethylsilyl)methyllithium. White solid obtained after filtration over a silica plug (SiO_2, *n*-pentane), 214 mg, 85% yield.

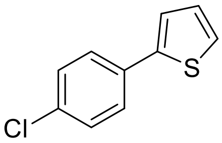

2-(4-chlorophenyl)thiophene (2ag).[27]
CAS Registry Number: 40133-23-1
Synthesized using catalytic systems B with 1-bromo-4-chlorobenzene (1 mmol, 191 mg) and 1200 µL of 2-thienyllithium. Off white solid obtained after filtration over a silica plug (SiO_2, *n*-pentane), 189 mg, 97% yield.

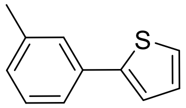

2-(*m*-tolyl)thiophene (2ah).[28]
CAS Registry Number: 85553-43-1
Synthesized using catalytic systems B with 1-bromo-3-methylbenzene (1 mmol, 171 mg) and 1200 µL of 2-thienyllithium. Collorless oil obtained after filtration over a silica plug (SiO_2, *n*-pentane), 146 mg, 84% yield.

Supplementary References

[1] Giannerini, M., Fañanás-Mastral, M., & Feringa, B. L. Direct catalytic cross-coupling of organolithium compounds. *Nature Chem*. **5**, 667–672 (2013).

[2] Giannerini, M., Hornillos, V., Vila, C., Fañanás-Mastral, M. & Feringa, B. L. Hindered aryllithium reagents as partners in palladium-catalyzed cross-coupling: Synthesis of tri- and tetra-*ortho*-substituted biaryls under ambient conditions. *Angew. Chem. Int. Ed*., **52**, 13329–13333 (2013).

[3] Hornillos, V., Giannerini, M., Vila, C., Fañanás-Mastral, M. & Feringa, B. L. Catalytic direct cross-coupling of organolithium compounds with aryl chlorides. *Org. Lett*. **15**, 5114–511 (2013).

[4] Carrera, N., Gutierrez, E., Benavente, R., Villavieja, M. M., Albeniz, A. C. & Espinet, P. Stannylated polynorbornenes as new reagents for a clean Stille reaction. *Chem. Eur. J.* **14**, 10141–10148 (2008).

[5] Lipshutz, B. H., Isley, N. A., Fennewald, J. C & Slack, E. D. On the way towards greener transition-metal-catalyzed processes as quantified by E factors. *Angew. Chem. Int. Ed*. **52**, 10952–10958 (2013).

[6] Hanley, P.S., Ober, M.S., Krasovski, A.L., Whiteker, G.T., Kruper, W.J., Nickel- and Palladium-catalyzed coupling of aryl fluorosulfonates with aryl boronic acids enabled by sulfuryl fluoride. *ACS Catal.***5,** 5041-5046 (2015).

[7] Vuoti, S., Autio, J., Haukka, J. & Pursiainen, D. Effective new palladium catalysts for the Suzuki coupling of various haloxylenes to prepare sterically hindered bi- and triaryls. *Inorg. Chim. Acta*. **362**, 4685–4691 (2009).

[8] Milde, B., Packheiser, R., Hildebrandt, S., Schaarsmidt, D., Rüffer, T. & Lang, H. Imidazole phosphines: Synthesis, reaction chemistry, and their use in Suzuki C,C cross-coupling reactions. *Organometallics*, **31**, 3661−3671 (2012)

[9] Baier, H., Kelling, A. & Holdt, H-J. PEPPSI-Effect on Suzuki–Miyaura reactions using 4,5-dicyano-1,3-dimesitylimidazol-2-ylidene-palladium complexes: A comparison between trans-ligands. *Eur. J. Inorg. Chem*. **11**, 1950−1957 (2015).

[10] Xu, M.; Li, X.; Sun, Z.; Tu, T. Suzuki–Miyaura cross-coupling of bulky anthracenyl carboxylates by using pincer nickel N-heterocyclic carbene complexes: an efficient protocol to access fluorescent anthracene derivatives. *Chem. Commun.*, **49**, 11539−11541 (2013).

[11] Song, C., Ma, Y., Chai, Q., Ma, C., Jiang, W. & Andrus, M. B. Palladium catalyzed Suzuki–Miyaura coupling with aryl chlorides using a bulky phenanthryl *N*-heterocyclic carbene ligand. *Tetrahedron*, **61**, 7438−7446 (2005).

[12] Becht, J-M., Catala, C., Le Drain, C. & Wagner, A. Synthesis of biaryls via decarboxylative Pd-catalyzed cross-coupling reaction. *Org. Lett.* **9**, 1781−1783. (2007).

[13] Vila, C., Hornillos, V., Giannerini, M., Fananas-Mastral, M. & Feringa, B. L. Palladium-catalysed direct cross-coupling of organolithium reagents with aryl and vinyl triflates. *Chem. Eur. J*. **20**, 13078−13083 (2014)

[14] Shi, S. & Zhang, Y. Silica-assisted Suzuki–Miyaura reactions of heteroaryl bromides in aqueous media *Green Chem.* **10**, 868−872 (2008).

[15] Xu, H., Ekoue-Kovi, K. & Wolf, C. Palladium-phosphinous acid-catalyzed cross-coupling of aryl and acyl halides with aryl-, alkyl-, and vinylzinc reagents. *J. Org. Chem*. **73**, 7638−7650 (2008).

[16] Cahiez, G., Duplais, C. & Buendia, J. Manganese-catalyzed oxidative cross-coupling of Grignard reagents with oxygen as an oxidant. *Angew. Chem. Int. Ed.* **48**, 6731−6734 (2009).

[17] Bluemke, T. D., Clegg, W., García-Alvarez, P., Kennedy, A. R., Koszinowski, K., McCall, M. D. Russo, L. & Hevia, E. Structural and reactivity insights in Mg–Zn hybrid chemistry: Zn–I exchange and Pd-catalysed cross-coupling applications of aromatic substrates. *Chem. Sci.*, 5, 3552−3562 (2014).

[18] Liu, Y., Shao, X., Zhang, P., Lu, L. & Shen, Q. Trifluoromethyl-substituted sulfonium ylide: Rh-catalyzed carbenoid addition to trifluoromethylthioether. *Org. lett.* **17**, 2752−2755 (2015).

[19] Liang, Q., Xing, P., Huang, Z., Dong, J., Sharpless, K. B., Li, X. & Jiang, B. Palladium-catalyzed, ligand-free Suzuki reaction in water using aryl fluorosulfates. *Org. lett.* **17**, 1942−1945 (2015).

[20] Hoffmann, I., Blumerröder, B., Thumann, S.O., Dommer, S. & Schatz, J. Suzuki Cross-coupling in Aqueous media, *Green Chemistry*, **17**, 3844−3857 (2015).

[21] Reddy, G. S. & Tam, W. Monoalkylation of dichloroarenes with Grignard reagents catalyzed by a nickel complex. *Organometallics*, **3**, 630−632 (1984).

[22] Giannerini, M., Fananas-Mastral, M. & Feringa, B. L. Direct catalytic cross-coupling of organolithium compounds. *Nature Chem.* **2013**, *5*, 667−672.
[23] Molander, G.A., Yun, C. Ribagorda, M. & Biolatto, M. B-Alkyl Suzuki−Miyaura cross-coupling reactions with air-stable potassium alkyltrifluoroborates. *J. Am. Chem. Soc.* **68**, 5534−5539 (2003).
[24] Larsen, M. A., Wilson, C.V., Hartwig, J. F., Iridium-catalyzed borylation of primary benzylic C-H bonds without a directing group: scope, mechanism and origins of selectivity, *J. Am. Chem. Soc.*, **137**, 8633-8643 (2015)
[25] Nagaki, A., Moriwaki, Y., Haraki, S., Kenmoku, A., Takabayashi, N., Hayashi, A. & Yoshida, J. Cross-coupling of aryllithiums with aryl and vinyl halides in flow microreactors. *Chem. Asian, J.*, **7**, 1061−1068 (2012).
[26] Heijnen, D., Hornillos, V., Corbet, B.P., Giannerini, M. & Feringa, B. L. Palladium-catalyzed C(sp^3)–C(sp^2) cross-coupling of (trimethylsilyl)methyllithium with (hetero)aryl halides. *Org. Lett.* **17**, 2262−2265 (2015).
[27] Ronsheim, M & Araldi, G. Indazole derivates useful as melanin concentrating receptor ligands. PCT patent application WO 2008061109 (A2) (2008).
[28] Maddali, L. N. R., Debasis, B. & Dhanorkar, R. J. Synthesis of functionalized 2-arylthiophenes with triarylbismuths as atom-efficient multicoupling organometallic nucleophiles under palladium catalysis. *Synlett.* **9**, 1324−1330 (2011).
